# soibean: High-Resolution Taxonomic Identification of Ancient Environmental DNA Using Mitochondrial Pangenome Graphs

**DOI:** 10.1093/molbev/msae203

**Published:** 2024-10-03

**Authors:** Nicola Alexandra Vogel, Joshua Daniel Rubin, Anders Gorm Pedersen, Peter Wad Sackett, Mikkel Winther Pedersen, Gabriel Renaud

**Affiliations:** Department of Health Technology, Section for Bioinformatics, Technical University of Denmark, Kongens Lyngby, Denmark; Department of Health Technology, Section for Bioinformatics, Technical University of Denmark, Kongens Lyngby, Denmark; Department of Health Technology, Section for Bioinformatics, Technical University of Denmark, Kongens Lyngby, Denmark; Department of Health Technology, Section for Bioinformatics, Technical University of Denmark, Kongens Lyngby, Denmark; Centre For Ancient Environmental Genomics, Globe Institute, University of Copenhagen, Copenhagen K, Denmark; Department of Health Technology, Section for Bioinformatics, Technical University of Denmark, Kongens Lyngby, Denmark

## Abstract

Ancient environmental DNA (aeDNA) is becoming a powerful tool to gain insights about past ecosystems, overcoming the limitations of conventional fossil records. However, several methodological challenges remain, particularly for classifying the DNA to species level and conducting phylogenetic analysis. Current methods, primarily tailored for modern datasets, fail to capture several idiosyncrasies of aeDNA, including species mixtures from closely related species and ancestral divergence. We introduce soibean, a novel tool that utilizes mitochondrial pangenomic graphs for identifying species from aeDNA reads. It outperforms existing methods in accurately identifying species from multiple closely related sources within a sample, enhancing phylogenetic analysis for aeDNA. soibean employs a damage-aware likelihood model for precise identification at low coverage with a high damage rate. Additionally, we reconstructed ancestral sequences for soibean’s database to handle aeDNA that is highly diverged from modern references. soibean demonstrates effectiveness through simulated data tests and empirical validation. Notably, our method uncovered new empirical results in published datasets, including using porpoise whales as food in a Mesolithic community in Sweden, demonstrating its potential to reveal previously unrecognized findings in aeDNA studies.

## Introduction

Ancient DNA (aDNA) provides a crucial window into identifying eukaryotic species from ancient remains by giving additional insight into archaeological and paleontological findings. However, fossils and other macroscopic remains are merely partial sources of information. Recently, ancient environmental DNA (aeDNA) has changed our understanding of past environments and species compositions in both time and space. Throughout an organism’s lifetime, it leaves genetic traces in the environment, in deposits such as sediment or permafrost (Hofreiter et al. 2003; Willerslev et al. 2003; Lydolph et al. 2005; Willerslev and Cooper 2005; Haile et al. 2007). The extraction and amplification of aeDNA allow for a widely distributed exploration of past ecological environments and populations from chosen sample sites (Jørgensen et al. 2012; Pansu et al. 2015; Graham et al. 2016; Pedersen et al. 2016; Ficetola et al. 2018; Dussex et al. 2021; Zavala et al. 2021). However, analyzing aDNA from the environment and bones poses many challenges. Firstly, aDNA is characterized by being highly fragmented (Pääbo 1989; Hofreiter et al. 2001) and modified by chemical damage such as deamination patterns (resulting in C to T and G to A substitutions) (Briggs et al. 2007; Prüfer et al. 2010). This causes changes in the similarity to the reference genomes used for taxonomic identification (Martiniano et al. 2020; Poullet and Orlando 2020). In addition, aDNA analysis must also consider the evolutionary processes, hence genetic distance between the organism and the reference genome and even the absence of a reference genome (Poinar et al. 2006; Schubert et al. 2012).

aeDNA inherits all the challenges associated with aDNA, while also presenting the added complexity of being a mixture of DNA from various sources. The accurate taxonomic classification of aeDNA fragments is greatly influenced by the relative abundance of DNA from each contributing source. Therefore, taxonomic classification can be either of low specificity (e.g. class, order, family) or high specificity (e.g. species, subspecies). With lower abundance, achieving a high taxonomic specificity is often more challenging due to a lack of unique genetic identifiers (Slon et al. 2022). In mitochondrial aeDNA analysis, results are often summarized at a lower taxonomic specificity (Slon et al. 2017; Kjær et al. 2022) when using standard classification methods like a naive lowest common ancestor (LCA) algorithm (Bender et al. 2005; Huson et al. 2007; Wang et al. 2022). A newer classification tool, euka, also classifies at lower taxonomic resolutions (Vogel et al. 2023). This classification method aids a confident validation of identified taxa via damage pattern estimation (Michelsen et al. 2022) or estimation of breadth and depth of coverage due to an increased amount of aeDNA fragments for a given taxon.

One method for high-resolution taxonomic assignment was proposed with HAYSTAC (Dimopoulos et al. 2022). HAYSTAC provides verification filters (e.g. likelihood filter, coverage evenness filter) for accurate species detection. Its all-versus-all mapping approach with Bowtie2 (Langmead and Salzberg 2012) considers all possible mapping positions, including those within highly conserved regions across species, which are usually ignored due to their inability to discriminate at the species level. For aeDNA analysis, these regions can be useful due to the sparsity of the data. We use HAYSTAC as our baseline model as it allows us to provide a user-built database. However, it does not account for private mutations or place samples within a phylogenetic reference. This limitation makes it challenging to identify the ancestral species.

Another method to more confidently assign classifications to a species or lower is phylogenetic placement, in which a consensus is called from the extracted fragments and placed on a phylogenetic tree based on sequence similarity (Bouckaert et al. 2019; Gelabert et al. 2021; Vernot et al. 2021). However, aeDNA data are often too low coverage to reliably call a consensus and, therefore, unfitted for phylogenetic placement. Furthermore, this problem becomes intractable if multiple species from the same genus (e.g. Arctic, Mountain, and Snowshoe hares) (Wang et al. 2021) or closely related species (Pedersen et al. 2021) exist.

To our knowledge, the only tool for species detection in low-coverage aDNA data is pathPhynder (Martiniano et al. 2022). pathPhynder considers unique SNPs to identify the most likely species. pathPhynder considers all derived and ancestral SNPs on a phylogenetic tree and is, therefore, able to infer a potential ancestral state of a species, making it extremely valuable for aDNA analysis (Kjær et al. 2022). However, pathPhynder is limited to single-source estimations. Multiple sources must be mapped beforehand and analyzed individually (Pedersen et al. 2021), which can adversely affect abundance estimates. Moreover, pathPhynder does not consider insertions or deletions in alignments, potentially discarding useful information.

We introduce soibean, a new subcommand of vgan (https://github.com/grenaud/vgan) for high-resolution taxonomic placement of aeDNA using mitochondrial pangenome graphs in conjunction with Bayesian inference methods. Pangenome graphs are reference data structures that mitigate reference bias by representing multiple genomic sequences simultaneously (Garrison et al. 2018; Martiniano et al. 2020; Sirén et al. 2021). soibean’s input is a FASTQ file consisting of aDNA fragments that have been previously classified to a lower taxonomic specificity, such as family level (e.g. with an LCA tool or euka). soibean then deconvolves reads into each contributing source at the species level and subsequently places them in their phylogenetic context. Our algorithm works as follows: (i) We align the aeDNA fragments to a curated and quality-controlled database of 326 arthropodic and tetrapodic taxa, including reconstructed ancestral states, allowing for variation unseen in modern reference genomes. (ii) soibean then uses Markov Chain Monte Carlo (MCMC) sampling to estimate the most likely placement on a phylogenetic tree branch and the relative abundances of each source, allowing robust identification from as little as 50 fragments. (iii) soibean’s results provide credible intervals and diagnostic metrics for all parameters of each source. Crucially, we can identify ancestral states and visualize confidence in phylogenetic placements. If a source has scarce data, either due to low relative abundance or simply low coverage for the taxon, our algorithm displays the uncertainty as the MCMC chains will sample widely across the tree branch. Runtimes depend on the number of iterations and input reads but ranged from 0.5 to 450 hours on example data we analyzed, memory usage on the same data stayed consistent at 1.5 GB.

This manuscript demonstrates soibean’s specificity and sensitivity on simulated datasets (one to four different sources on five different taxa) before highlighting its consistency with empirical data. Lastly, we showcase soibean’s ability to discover novel results from previously published datasets, including discovering harbor porpoise as a food source for a Mesolithic community in Sweden.

## Results

**Fig. 1.**
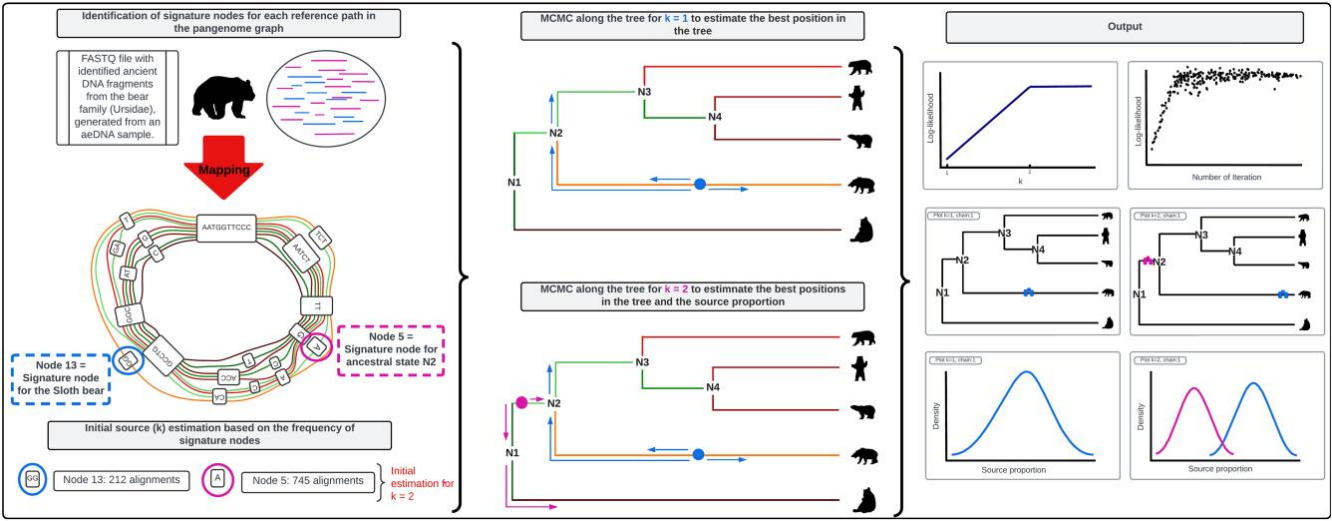
soibean’s main workflow starts by mapping a filtered FASTQ file against the selected taxon graph. The alignment is analyzed, and an initial source estimation based on signature note-set frequencies is calculated. soibean runs an MCMC algorithm from 1 to the number of estimated sources and calculates a source proportion and a branch position for each source based on a maximum likelihood function. The MCMC diagnostics provide statistics about the most likely number of sources, their proportion, and branch position. soibean provides extensive plotting scripts to visualize its results.

To generate our results, we used soibean’s default settings (commands for the simulated datasets in supplementary section 1.4, Supplementary Material online and empirical datasets in supplementary section 1.6, Supplementary Material online) on different pangenome graphs from soibean’s database (construction pipeline illustrated in supplementary fig. S1, Supplementary Material online). soibean’s general workflow starts with an initial estimation of the number of sources present in the sample, followed by the MCMC estimation of each source most likely placement on the phylogenetic tree and their relative abundance, and ends with MCMC diagnostics and output visualization (see Fig. 1). Detailed explanations and methodological descriptions of the various aspects of our algorithm are found in the Methods section.

soibean runtime depends on the size of the dataset and the number of MCMC iterations. An average time consumption chart for users is presented in supplementary fig. S26, Supplementary Material online, with a comprehensive computational analysis available in supplementary section 1.1, Supplementary Material online.

### Simulations

To test soibean, we simulated six different datasets (details can be found in supplementary section 1.2, Supplementary Material online). For the first dataset, we simulated a single source from an ancestral state sequence from the family of bears (Ursidae). From the same family, we simulated another three datasets for two-source samples. The first two-source mixture contains two closely related bear species (∼98.8% genome similarity), the second mixture contains two less closely related bear species (∼93.1%) and lastly, a mix of two divergent bear species (∼83.4%). We simulated a three-source dataset using a family of winged insects (Saturniidae), sampling from two emperor moth species and an ancestral state. The last dataset was to simulate a four-source dataset, where we used the family of earless seals (Phocidae). We simulated reads from four species of the same genus (*Phoca*). Simulations were created with gargammel (Renaud et al. 2017), where each dataset has a fragment length distribution following a log-normal distribution with μ=3.7344 and a σ=0.35 as commonly seen in aDNA studies and deamination rates taken from Günther et al. (2015). We merged the simulated reads with leeHom using ancient parameters (Renaud et al. 2014). All simulated datasets are in our provided test data https://github.com/nicolaavogel/soibeanDatabase.

All simulations were used for benchmarking against HAYSTAC as our baseline model. We additionally compared our single-source simulations with pathPhynder. Details can be found in supplementary section 1.3, Supplementary Material online and the commands used in supplementary section 1.4, Supplementary Material online.

#### Single-source

**Fig. 2.**
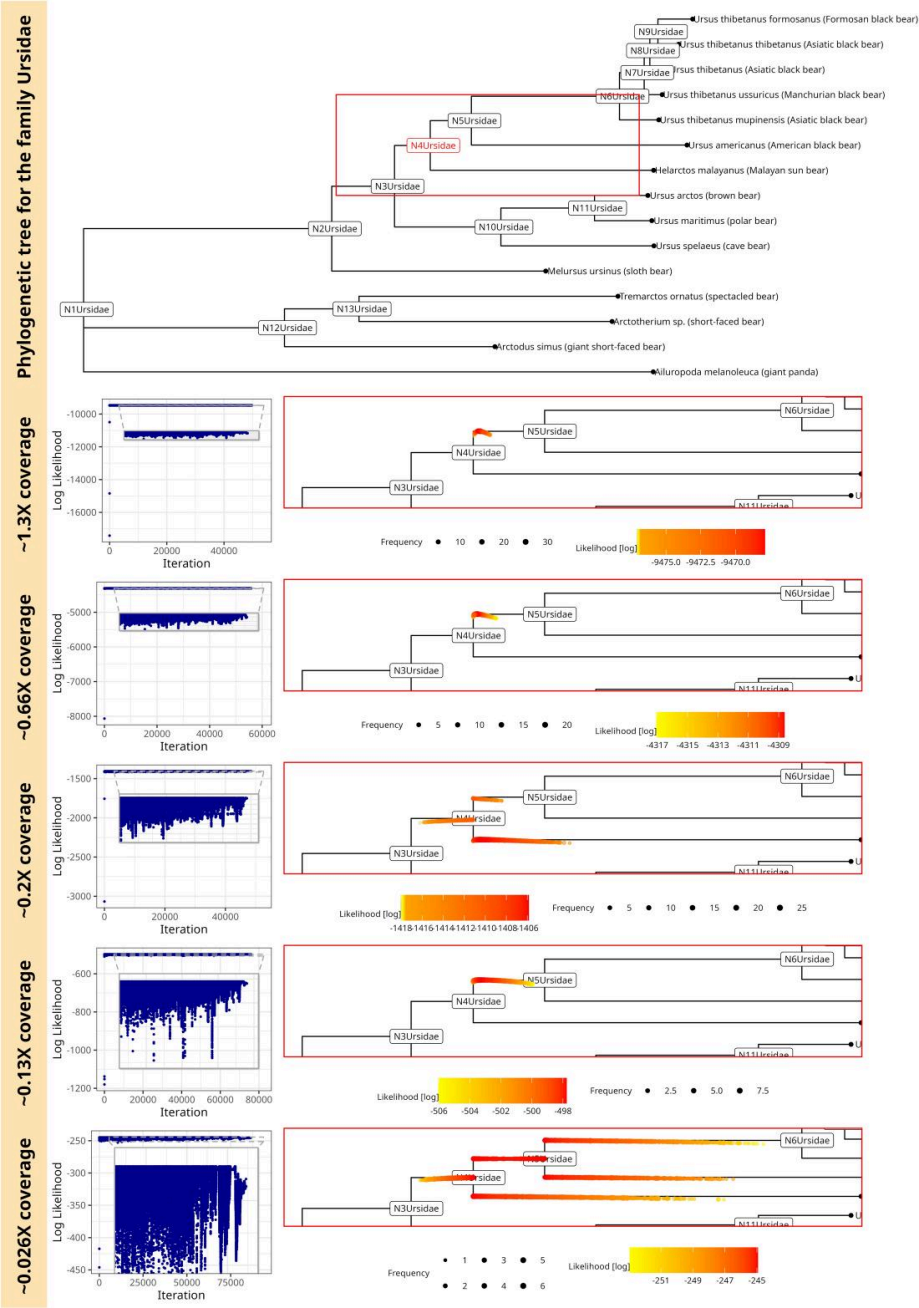
soibean results for simulated ancient fragments using a single source of DNA from the ancestral state N4 of the bear family (Ursidae). The complete phylogenetic tree for the family Ursidae marked in red is the sampled ancestral state; the square shows the embedded zoomed-in portion of the tree in the plots below. The traceplot for the MCMC sampling and the accepted MCMC moves with their log-likelihood on the zoomed-in tree for coverage of ∼1.3X(500fragments),∼0.66X(250fragments),  ∼0.2X(75fragments),  ∼0.13X(50fragments),  ∼0.026X(10fragments). The MCMC sampling is more uncertain with lower coverage, noted by more variable accepted moves visible in the trace plot and the zoomed-in tree plots.

Our single-source sample is simulated from the ancestral state N4 of the bear family (Ursidae). To show soibean’s robustness, we downsampled the data from ∼1.3X coverage to ∼0.026X coverage (see Fig. 2). Figure 2 shows the complete phylogenetic tree for the family of bears on the top. For each downsampled coverage, we show the MCMC’s trace plot on the left side, which has a dot for every proposed move log-likelihood and demonstrates the initial finding of the correct tree branch and the optimal exploration of the parameter space by using independent sampling. The right side shows the zoomed-in portion of the phylogenetic tree, with every accepted MCMC move as a red dot. It can be seen how locations close to the true node are sampled and how the uncertainty about the location increases for lower coverage (red and yellow dots cover a larger area surrounding the true node). Figure 2 demonstrates soibean’s accuracy down to ∼0.13X coverage, corresponding to ∼50 aDNA fragments aligned to the mitochondrial genome. We can see that the certainty of branch positions decreases with lowered coverage as we accept moves across the entire branch. At ∼0.026X coverage, we are unable to define the correct origin of the source. However, all accepted MCMC moves are adjacent to the true node (specifically, within the true nodes’ parent, sibling, and child branches).

Comparing soibean’s results to existing methods, we can observe that pathPhynder can accurately identify the correct ancestral source (tree node N4) down to ∼0.2X coverage with its best path method (supplementary figs. S2–S4, Supplementary Material online). At lower coverage, pathPhynder’s best path method predicts an incorrect source. However, source predictions stay within the targeted nodes’ parent and child nodes (supplementary figs. 5 and 6, Supplementary Material online). Additionally, we tested pathPhynder maximum likelihood method, which performed identical to soibean, correctly identifying the ancestral state N4 to ∼0.13X coverage (supplementary figs. 7–10, Supplementary Material online). Only at the lowest coverage pathPhynder’s maximum likelihood method classifies incorrectly to the child (N6) of the ancestral state N4 (supplementary fig. 11, Supplementary Material online). Overall, soibean shows more robustness to lower coverage than pathPhynder’s best path method and performs identical to its maximum likelihood method for identifying ancestral states. HAYSTAC as our baseline model faces challenges identifying ancestral states despite sequences being provided (supplementary table 1, Supplementary Material online). No ancestral state was identified at any level of coverage, likely because HAYSTAC was not designed to include tree topologies.

#### Two Sources

**Fig. 3.**
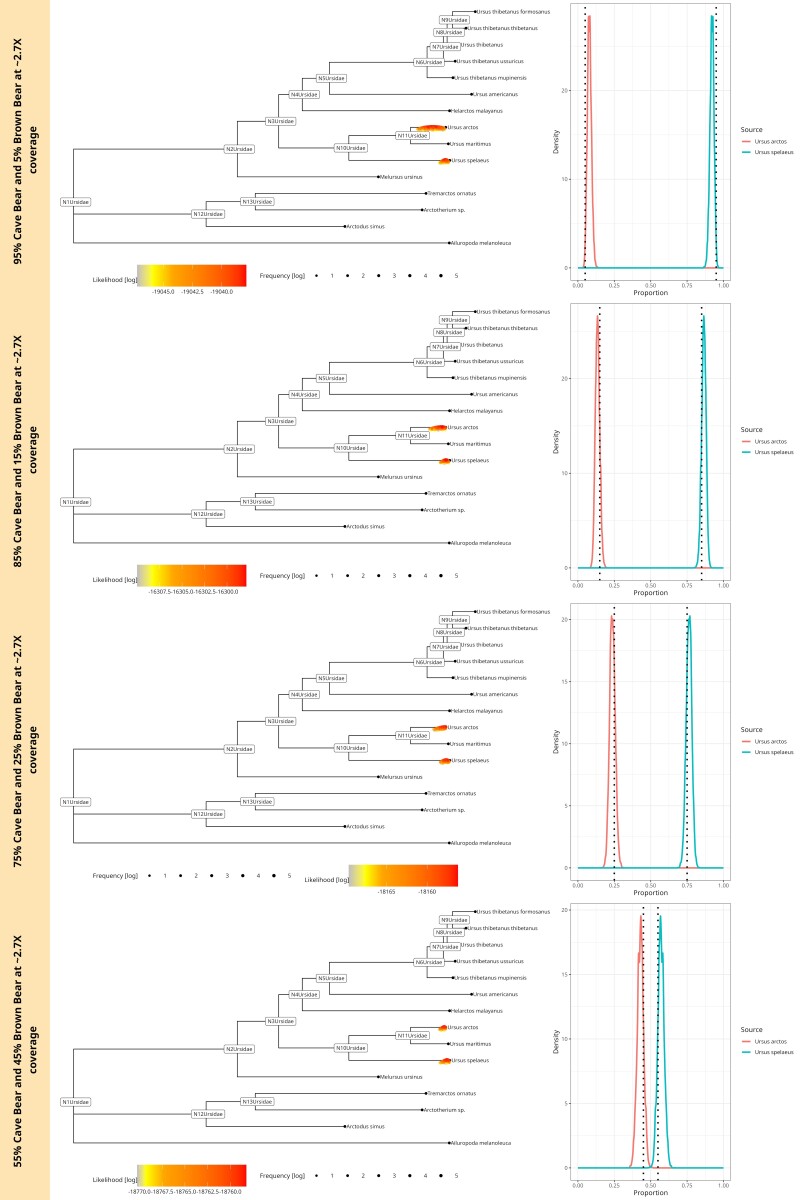
soibean results for simulated ancient fragments using a blend of two sources from two species with a similarity of 93.1% from the family bears (Ursidae) at ∼2X coverage. The plot shows four different mixtures at 55%--45%,75%--25%,85%--15%,and95%--5% of the Cave bear and the Brown bear. The corresponding phylogenetic trees are displayed on the left: we plotted every accepted MCMC move colored by likelihood value on the tree. The accepted moves are positioned above or below the tree, corresponding to a higher or lower likelihood value than the median, respectively. Each neighboring plot shows the posterior proportion distribution, including the simulated proportion with a black dotted line.

For our two-source simulations, we used a mixture of two different bears: a Cave bear and a Brown bear with mitogenome similarity ∼93.1%. We simulated four proportions 95%--5%,85%--15%,75%--25%, and 55%--45% with total average coverage of ∼2.5X (1,000 aDNA fragments). These simulations are similar to the real data seen in Pedersen et al. (2021), which had a total of 740 aDNA fragments mapping for two distinct sources combined in one sample. For the mixture of the Cave and the Brown bear, soibean identifies both sources for every simulated proportion at ∼2.5X coverage (see Fig. 3). We can observe that the lower the proportion of a source (here: the Brown bear), the more uncertain the estimation of the correct position on the branch. This mirrors the observations from our single-source experiments.

To demonstrate soibean’s robustness, we downsampled the mixture of two bears to ∼1.3X,∼0.7X  and∼0.25X coverage. soibean identifies the correct sources for every level of coverage (supplementary figs. 17, 18, and 29, Supplementary Material online) except for the 95%–5% mixture at ∼0.25X coverage (see supplementary fig. 19, Supplementary Material online).

We repeated this experiment with a more dissimilar (83.4% similarity—Giant Panda bear and American Black bear) and a more similar (98.8% similarity—Tibetan and Taiwan Black bear) mixture of two bears for all four coverage levels. Detailed results can be found in supplementary section 1.5, Supplementary Material online with supplementary figs. 12 to 22, Supplementary Material online. Generally, the higher the similarity, the higher the needed coverage for soibean to distinguish between sources successfully.

As a baseline, HAYSTAC performed comparably to soibean across all samples and mixtures, while pathPhynder was not developed to estimate more than one source. To demonstrate, we used the 55%–45% mixture of the cave and the brown bear, where pathPhynder interprets the mixture as a single-source and identifies the mixture’s lowest common ancestor (see supplementary fig. 23, Supplementary Material online). HAYSTAC’s results for the two-source mixtures exhibited more variability at ∼0.25X coverage, as outlined in supplementary table 1, Supplementary Material online. This variability largely arises from a scarcity of uniquely mapped reads to species reference genomes at reduced coverage levels. In contrast, soibean’s pangenomic reference enhances its ability to navigate the challenges of unique identifiers by reducing reference bias, as discussed in Martiniano et al. (2020).

### Three and Four Source Simulations

We simulated a three-source sample from a family of moths (Saturniidae), including the two emperor moths *Gonimbrasia tyrrhea* and *Gonimbrasia belina*. Additionally, we added the ancestral state N7 to the mixture and simulated a total of 1500 aDNA fragments, averaging a coverage of ∼4X. Mixture proportions were 47%--33%--20%. For our simulations of a four-source sample, we used the family of seals (Phocidae); specifically, we sampled 500 aDNA fragments for each of the four earless seals, namely *Phoca largha*, *Phoca vitulina*, *Phoca groenlandica*, and *Pusa hispida* creating a mixture of 25% each and a total coverage of about ∼5.4X. These numbers of simulated fragments were up-scaled from Pedersen et al. (2021) as we could not find an empirical aeDNA study identifying three or more sources from one family.

**Fig. 4.**
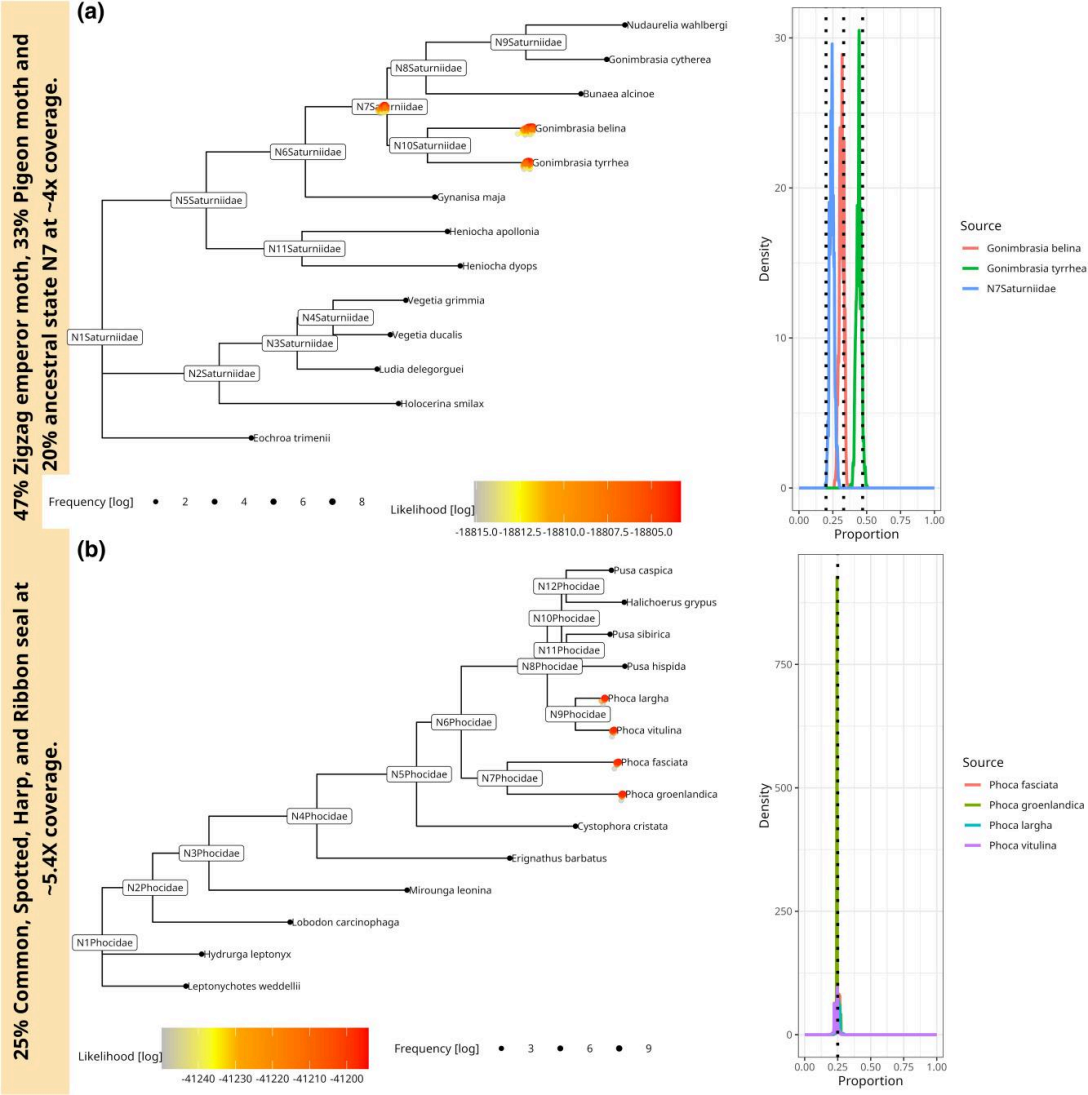
a) 44%--30%--23% mixture of a three-source simulated ancient sample from a family of winged insects (Saturniidae). The mixture contains two emperor moths (*G. tyrrhea* and *G. belina* as well as the ancestral state N7. We sampled to ∼4X coverage. The tree shows every accepted MCMC move, colored and placed by log-likelihood value, where a position above the tree branch represents a better likelihood than the median likelihood and a position underneath the tree branch a worse likelihood (right side). The right side of the plot shows soibean’s proportion estimation with the simulated true proportion represented with a black dotted line. b) 25%--25%--25%--25% mixture of a four-source simulated ancient sample from the family of seals (Phocidae). The mixture contains the earless seal species, namely *P. largha*, *P. vitulina*, *Phoca groenlandica*, and *Phoca fasciata* at ∼5.4X coverage (2,000 aDNA fragments). The tree shows every accepted MCMC move, colored, and placed by log-likelihood value plot, showing soibean’s proportion estimation with the simulated true proportion with a black dotted line.

Figure 4 shows soibean’s results for three a) and four b) simulated sources, clearly identifying the correct placements and proportions for each. A warning is produced in case a chain’s effective sample size (ESS) is below 200. This warning was triggered for the four-source sample. The ESS is essentially the number of independent MCMC samples (accounting for autocorrelation). A low ESS means that the quantiles of the posterior distribution will be poorly estimated (especially quantiles in the tails of the posterior, such as the 5% and 95%) and is an indication that the MCMC should be run for more iterations.

Again, we downsampled both samples to demonstrate soibean’s robustness. The three-source samples can be consistently identified until a coverage of ∼1X. At lower coverage, it becomes more difficult to identify the branch placement for the ancestral state N7 and more branch positions on the surrounding nodes of N7 are accepted (supplementary fig. 24, Supplementary Material online). For the four-source samples, soibean can identify the correct sources for each downsampled coverage if the signature node prediction is used to initiate the starting position on the tree (see supplementary fig. 25, Supplementary Material online). However, if we initialize randomly, soibean does not converge to the correct branch placements. Our diagnostics clearly identify the highest log-likelihood with the correct branch placements in the phylogenetic tree, suggesting that a higher number of iterations is necessary to converge to the true underlying posterior.

Our baseline model identifies the two emperor moths for the three-source sample but does not pinpoint the ancestral state, as seen in the single-source sample (see supplementary table 1, Supplementary Material online) for every simulated coverage. For the four-source sample, the baseline model identifies all four species down to coverage of ∼1.3X (Fig. 4).

### Empirical Data

We demonstrate soibean’s efficacy on empirical data by showcasing its results on four published datasets. First, we reanalyzed the 2-million-year-old sediment samples from Greenland’s Kap Københaven Formation (Kjær et al. 2022). The sample was sequenced using Illumina HiSeq and NovaSeq (ENA: PRJEB55522). The second dataset is an approximately 4,000-year-old sediment sample from Qeqertasussuk, Greenland, sequenced with Illumina HiSeq (ENA: PRJEB13329) (Seersholm et al. 2016). The third is a 25,000-year-old sediment sample from Satsurblia Cave in Georgia, sequenced using Illumina NovaSeq (ENA: PRJEB41420) (Gelabert et al. 2021). Lastly, we reanalyzed the metagenomic data sampled from pitch pieces used by a Mesolithic community in Huseby Klev, Sweden, dated to 9,500 years ago, which was sequenced using Illumina Hiseq X (Bioproject: PRJNA994900) (Kırdök et al. 2023). For each of the four samples, we downloaded the published data, trimmed adapters and merged the reads using leeHom (Renaud et al. 2014), removed PCR duplicates and low-complexity reads using sga (Simpson and Durbin 2012), and then inferred eukaryotic abundance using euka (Vogel et al. 2023).

#### Confirmatory Results

**Fig. 5.**
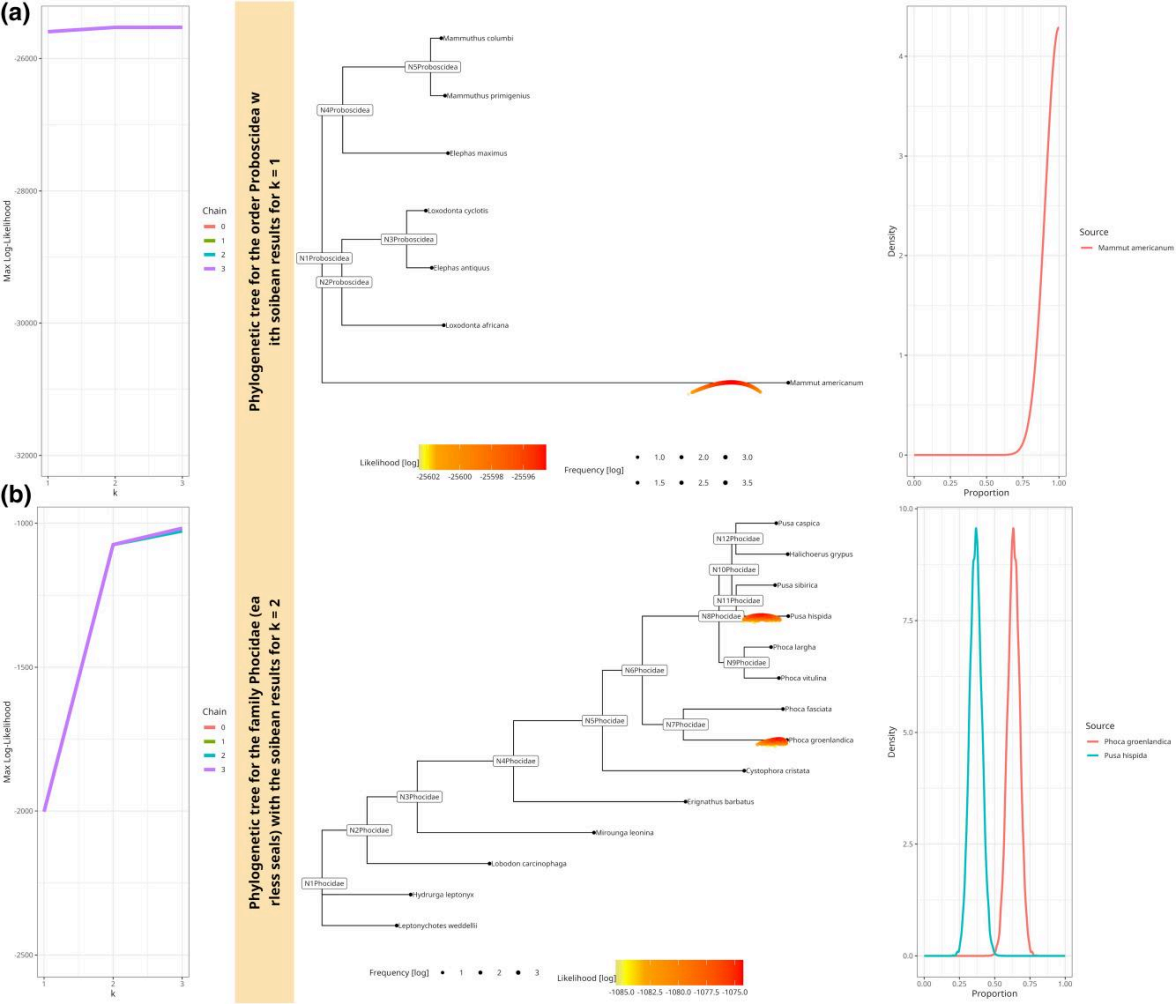
Confirmatory results for two independent studies a) soibean results for the 2-million-year-old empirical sample from Greenland. The filtered aDNA fragments from the order Proboscidea are analyzed using soibean’s standard parameters and a forced k=3. The plotted k-curve shows there to be no more than one source present. The phylogenetic tree has every accepted move plotted along the most likely branch. A move plotted below the branch shows a lower likelihood than the median, and a move plotted above the branch has a higher likelihood. Moves are colored by likelihood and show a clear result for the Mastodon, as seen in the original publication (Kjær et al. 2022). We observe a slight divergence from the reference genome, which could be caused by the high levels of deamination observed in the sample or genetic divergence over time. b) soibean’s results for the 4,000-year-old Greenlandic empirical sediment sample, where we analyzed the filtered aDNA fragments mapping to the family Phocidae. The k-curve on the left side of the plots clearly shows the presence of two contributing sources, which can be identified as the Harp seal *P. groenlandicus* and the Ringed seal *P. hispida*. This also aligns with the original publication. However, with soibean, we are able to add proportion estimates for the seals, which are found to be in a 60%–40% mixture.

The first empirical sample describes different families of mammals, including the family Elephantidae, which was concluded to be a mastodon (*Mammut americanum*) using pathPhynder (Kjær et al. 2022; Martiniano et al. 2022). Figure 5a shows soibean’s identification of the mastodon: first, the k-curve shows that a single source sufficiently explains the data; the likelihood does not show any significant increase for higher *k*s. Secondly, we show the estimated branch position for the mastodon. The higher the log-likelihood, the higher the confidence in the branch position. We visualize this confidence estimation in two ways: by color (as seen in the legend) and by the position of dots (accepted MCMC moves) above or underneath the branch. If a dot is above the tree branch, the log-likelihood is higher than the median, and vice versa. If a point is positioned precisely on the branch, it equals the median log-likelihood of that MCMC chain.

The second sediment sample focused on evidence for the presence of bowhead whales. However, the original publication also identified different species of seals (Seersholm et al. 2016), which we focused on reanalyzing. Figure 5b first shows the k-curve on the right side of the plot, which strongly suggests the presence of two sources in the sample but does not support a third source. When looking at the tree and the branch placement for the two sources, we can re-identify the Harp seal (*P. groenlandica*) and the Ringed seal (*P. hispida*), in a ratio of approximately 60%–40%. The specific proportion often gets lost when mapping due to higher taxonomic classifications of reads. After duplicate removal, 166 aDNA fragments were mapped. The extremely low coverage does not allow us to define an exact position on the branch.

#### Novel Results

For the third empirical cave sediment sample, the original publication focused on retrieving high-coverage mitochondrial genomes for three species (human, bison, and wolf) using shotgun metagenomics. They used phylogenetic placements to estimate the correct position in the species phylogenetic tree. Here, we focus on the reads that euka assigned to the family Bovidae. Supplementary fig. 27, Supplementary Material online shows a clear signature of two distinct Bovidae sources. soibean predicted the first one to be the European Bison (*Bison bonasus*), which is the same as found and analyzed in the original publication. Additionally, soibean picked up on a small signal (5%) from the West Caucasian tur (*Capra caucasica*). The publication describes a signal from the genus *Ovis*. However, no conclusion was reached due to low coverage. Based on the mitochondrial data analyzed with soibean, this second source could be the West Caucasian tur, which is native to Georgia and is believed to have been hunted at around the same time as the sample’s estimated age (Pinhasi et al. 2014; Gelabert et al. 2021). We tried to add a secondary analysis to verify our results, where we concatenated the reference mitogenomes of bison (NCBI accession NC_014044.1) and tur (NCBI accession NC_020683.1), mapped all reads using SHRiMP (Rumble et al. 2009) and extracted 54 reads mapping uniquely to the tur mitogenome. We plotted the deamination patterns for the alignment using bam2prof (Renaud et al. 2019) (commands and parameters can be found in supplementary section 1.6, Supplementary Material online). Due to the extreme sparsity of the data, the damage plot shows a high volume of noise from other substitutions (see supplementary fig. 28, Supplementary Material online). Subsequently, we cannot call a consensus from the tur data to place it phylogenetically for additional confirmation. This demonstrates the significance of soibean in identifying species from sparse data. The identification process opens up possibilities for employing laboratory enrichment methods to gain deeper insights into the ecological history of a sample.

**Fig. 6.**
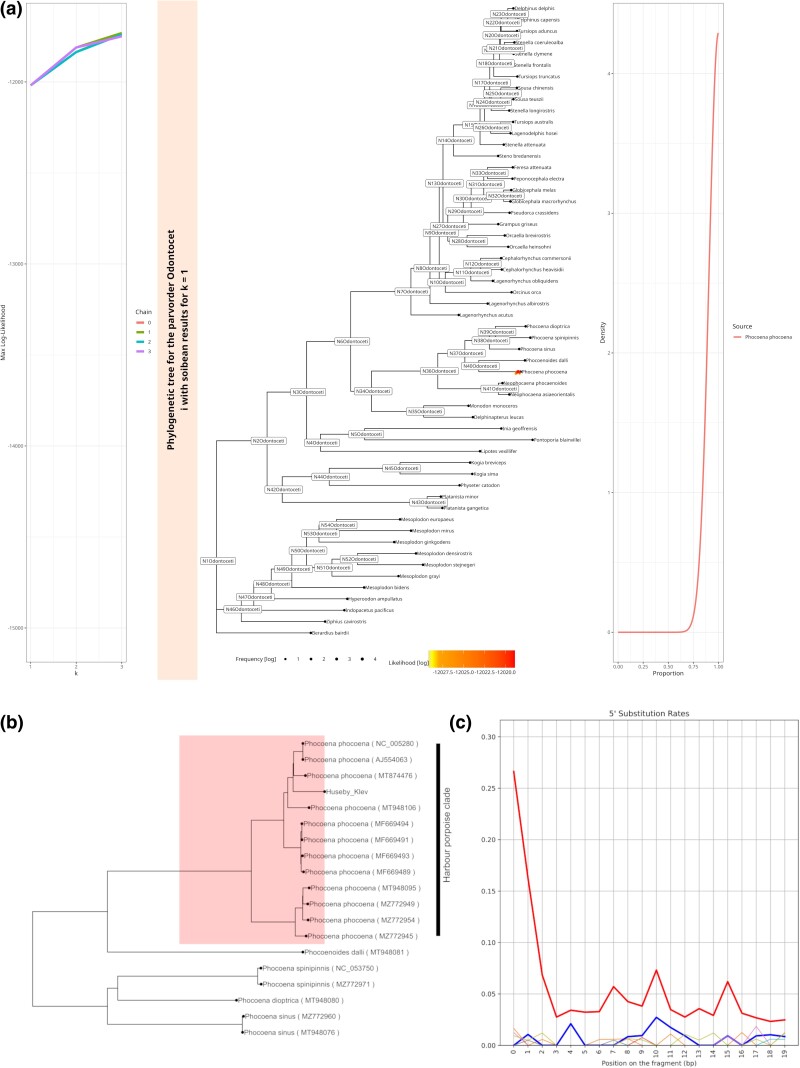
Novel results: a) soibean results for the 9500-year-old empirical metagenomic sample from pitch pieces found in Huseby-Klev on the northwestern coast of Sweden. We detected a previously unidentified taxon of toothed whales (Odontoceti). The filtered aDNA fragments from the parvorder Odontoceti are analyzed using soibean’s standard parameters and a forced k=3. The plotted k-curve indicates a single-source sample. The phylogenetic tree has every accepted move plotted in color and placement regarding their individual log-likelihood, displaying the most likely source to be the harbor porpoise (*P. phocoena*). b) Maximum-likelihood phylogenetic tree plot with all available mitochondrial genomes for the porpoise including the consensus sequence for the Huseby-Klev sample. c) Deamination rates for the 5’ end substitutions of the sample. The 3’ end substitutions show a clearly elevated G to A pattern (see supplementary fig. 31, Supplementary Material online).

The final reanalyzed sample is a metagenomic sample from chewed pitch pieces of a Mesolithic community in Sweden. The original publication focused on the oral microbiome but also identified multiple eukaryotes, including foxes, salmon, deer, mallards, and apples, as potential food sources (Kırdök et al. 2023). We used euka to reanalyze the dataset and detected all taxa of the original publication plus one additional taxon, Odontoceti (toothed whales). We extracted all aDNA fragments for this taxon and filtered for low-complexity and PCR duplicates. soibean estimated the filtered input to be a single-source sample from a harbor porpoise (*Phocoena phocoena*) (see Fig. 6a) k-curve, which shows a slight rise in the log-likelihood from k=1 to k=2, However, the estimated sources for k=2 and k=3 show geographically unlikely species: see supplementary figs. 29 and 30, Supplementary Material online), thus indicating that the data can be explained using a single-source. The harbor porpoise presently inhabits the Baltic Sea, and its bones have been found before at the same location (Huseby-Klev, Sweden) dated to the same period (Hansson et al. 2019; van den Hurk 2020). We used the same input file to confirm our results and mapped it against the harbor porpoise mitochondrial reference genome (NCBI accession number: NC005280.1). We estimated the deamination patterns from the BAM file (see Fig. 6c and supplementary fig. 31, Supplementary Material online) and afterwards used the alignment and damage profiles to estimate a damage-aware consensus sequence using endoCaller from schmutzi (Renaud et al. 2015). The consensus sequence was aligned with other reference genomes from the genus using prank and converted into a phylogenetic tree using RaxML (commands and parameters can be found in supplementary section 1.6, Supplementary Material online). Figure 6b shows the source’s placement within the harbor porpoise clade. This finding suggests that the Mesolithic community in Sweden also used the harbor porpoise as a food source.

## Discussion

soibean enhances aeDNA taxonomic specificity by distinguishing closely related species and estimating abundances with an MCMC algorithm, addressing the challenge of identification in low-abundance samples and advancing deep-time ecosystem insights. First, it is important to emphasize that our methodology, centered exclusively on mitochondrial aeDNA, may constrain the comprehensive analysis of exceedingly ancient samples (McCauley et al. 2024). In tests with simulated data, we confirmed soibean’s reliability for single-source data processing at minimum coverage depth of ∼0.13X. This study aimed to determine the ancestral state in bears, a well-defined taxonomic group, ensuring high accuracy in ancestral state reconstructions. However, soibean’s performance may be less reliable with less-defined or more evolutionary divergent taxa, leading to uncertain ancestral sequence reconstructions. Caution is recommended when applying soibean to highly divergent or low-entropy taxa, often seen within the Arthropoda, due to the ongoing challenge of accurate arthropod classification in aeDNA research and lack of extensive testing in these areas.

In our study, we tested soibean’s ability to identify up to four sources within a genus, noting it theoretically could distinguish more but was limited by practical constraints. We faced challenges with closely related sources, as soibean needed higher coverage for accurate differentiation in taxa with low divergence due to few unique signature nodes. Improving the MCMC algorithm’s solution accuracy involved using signature node estimation for initialization and increasing iterations, particularly vital when identifying over two sources and at lower coverage.

We recognize soibean’s higher computational demand and slower speed compared to other tools. However, its precision and reliability, especially for low-coverage Tetrapod and Arthropod aeDNA/aDNA samples, establish it as a crucial classification tool. Our visualization scripts and diagnostic outputs aid in efficient result interpretation. Given the challenges in species classification due to aeDNA’s peculiarities, accurately quantifying uncertainty is vital. soibean’s use of Bayesian inference provides confidence levels for parameter estimation, proofing highly effective for aeDNA’s complex scenarios.

## Methods

### General Workflow

To describe soibean’s workflow, we will (i) specify the input data, (ii) define how to access the database, and (iii) describe the main function. (i) soibean accepts FASTQ input (single-end, paired-end interleaved, or paired-end separate) where the sequencing adapters have been removed, overlapping portions merged (see recommendations in Lien et al. 2023), and PCR duplicates removed. soibean can take data generated by shotgun-sequencing as well as capture enrichment. However, the FASTQ input should be DNA fragments previously assigned to the same higher taxonomic rank, e.g. all reads mapping to the taxon Ursidae (bears). This prefilter is important as soibean does not have a model of spurious mappings due to bacterial DNA or DNA originating from another taxon. We recommend using euka or an alignment-based mapping + LCA approach to do the initial classification. The classified reads can be subsetted and extracted from the original input FASTQ to be used with soibean. (ii) Once the taxon of interest is defined, the user can extract the pangenomic subgraph of the taxon of interest from the larger database using a provided bash script (https://github.com/grenaud/vgan/tree/main/share/vgan/soibean_dir) that takes the taxon name as input. The subgraph corresponding to this taxon is extracted from the combined pangenome graph by its start and end node IDs. The script automatically produces all index files required by vg giraffe (Sirén et al. 2021). Afterwards, the extracted taxon can be specified with the --dbprefix flag when using soibean. The specification of the database prefix simultaneously accesses the correct corresponding phylogenetic tree. All details about the construction of our database can be found in supplementary section 2.1, Supplementary Material online. (iii) Once the input FASTQ mapping to the pangenomic component is done, we use a Markov Chain Monte Carlo (MCMC) sampling algorithm to estimate the most likely number of distinct contributing sources present in the sample and their respective placements on the (fixed) phylogenetic tree which is described in the subsections below. Test data can be found at https://github.com/nicolaavogel/soibeanDatabase, and examples of the usage for soibean are provided on GitHub https://github.com/grenaud/vgan. During the development of soibean, we utilized the help of ChatGPT-4, a language model developed by OpenAI, for coding and debugging tasks. We carefully evaluated all proposed code before integration into the final software.

### soibean Likelihood Function

Our model uses a maximum-likelihood framework to estimate the most likely number (denoted *k*) of contributing sources, their proportion θ (a length-k vector summing to one), and their most likely placements on branches β (a length-k vector of locations on the reference phylogenetic tree). Placements can be any position on the branches of the tree—not only nodes. This defines our model as M=(β,θ). For instance, if we have two equally contributing sources, then k=2 and θ=(0.5,0.5). The phylogenetic placement $\beta =(\beta_{1},\beta_{2})$ would then represent the placements on branches of the tree for each of the two sources.

We here use a uniform prior over the phylogenetic placement and abundance vectors. Both the prior and marginal probability are, therefore, constants, and according to Bayes’ Theorem, the posterior probability over the model parameters is consequently proportional to the likelihood (the probability of the data given the model parameters):


P(θ,β|D)∝P(D|θ,β),

where the data, *D*, is the set of aligned reads. Briefly, we seek θ,β that maximize P(D|θ,β). As we do not know the provenance of each read, we marginalize over each read for every source:


$$\begin{array}{rl} P(D\;|\;\theta ,\beta ) & =\prod_{\;fr\in D}\sum_{i=1}^{k}P(\theta_{i},\beta_{i})P(fr\;|\;\theta_{i},\beta_{i})\\ & =\prod_{\;fr\in D}\sum_{i=1}^{k}P(fr\;|\;\theta_{i},\beta_{i}) \end{array}.$$

Specifically, a fragment *fr* can come from one of the *k* possible sources. The prior probability that a fragment is from source *i* and having placement $\beta_{i}$ is $P(\theta_{i},\beta_{i})$. The probability that a fragment *fr* has a specific sequence is $P(fr\;|\;\beta_{i},\theta_{i})$. Since we do not know which of the *k* sources any given fragment is from, we compute the likelihood of a fragment by summing over these *k* possibilities. The overall likelihood for all fragments is then computed by multiplying their individual likelihoods, thus treating each DNA fragment as an independent observation and assuming duplicate fragments have been removed. The prior $P(\theta_{i},\beta_{i})$ can be omitted from the calculation because we use a flat (and hence constant) prior on both these parameters.

To calculate the likelihood of a branch placement on the tree, we make the simplifying assumption of treating each base *b* of fragment *fr* as independent, allowing us to multiply the probabilities of each nucleotide observation for each fragment:


$$P(fr\;|\;\beta_{i})=\prod_{b\in fr}P(b\;|\;\beta_{i})$$

To compute the probability of a given nucleotide observation, we calculate the probability of observing the base given the placement. Any placement on the branch of a tree can viewed as a position between two nodes (not to be confused with nodes in the pangenome graph, which represent sequences), a derived node $N_{D}$, which is closer to the leaves of the tree and an ancestral node $N_{A}$ which is closer to the root of the tree. Each tree node has a single reference path in the graph. For the nodes in the pangenomic graph, there are a certain number of reference paths that go through them. For our base *b*, there are two possibilities for any given tree node (i) the base was aligned to graph nodes associated with the tree node or (ii) the base missed the alignment to all or certain graph nodes associated with the tree node. We discuss both cases.

#### *b* is missing certain graph nodes for a given reference path

Due to the nature of the pangenome graph structure, it may be the case that the base in question is on a node untraversed by the putative reference path. We term such nodes “unsupported” by the path. In these cases, we treat $\frac{6}{7}$ of all bases as a sequencing error with a probability of $\frac{\epsilon }{3}$ and $\frac{1}{7}$ of all bases as match with probability 1−ϵ. ϵ is directly derived from the base quality reported from the sequencer. This is a slight update of the model for unsupported bases, which was used in HaploCart, another vgan subcommand (Rubin et al. 2023), which we find to be more accurate empirically as the taxa used in the soibean database have a higher genetic divergence than human mitochondrial haplotypes.

#### *b* aligns to graph nodes for a given reference path

If the base *b* is aligned to a node associated with a path that corresponds to either tree node along the tree branch (namely either $N_{D}$ or $N_{A}$), we compute the probability of either a match, a mismatch, a deletion, an insertion, an unresolved base or a softclip (an unaligned portion flanking an aligned fragment). For an aligned base, deletions and insertions have a probability of 0.02 based on an empirical study of human mitogenomes (Laricchia et al. 2022). Unresolved bases, as well as softclips, are treated as sequencing errors with a probability of $\frac{\epsilon }{3}$.

We are left estimating the probability of an aligned base *b* being a match or a mismatch. Three events could change a nucleotide: a mutation occurring with a probability *μ*, ancient damage with a probability *δ*, or a sequencing error occurring with probability ϵ. A match would be the absence of all these events. However, it is also possible, but less likely, that a match occurred due to a mutation followed by damage, which reverted the base to the original one. We compute the probability of all these scenarios. This means we want to compute the probability of $P(b\;|\;b_{g})$, where $b_{g}$ is the reference base.

We first look at the probability of a mutation given a position on our tree branch *t* under an HKY model (Hasegawa et al. 1985). A detailed explanation of how we calculate *μ* and the resulting probabilities for a match, transition or transversion can be found in supplementary section 2.2, Supplementary Material online. The principal calculation is as follows: the further we move from $N_{D}$ towards its ancestor $N_{A}$ (the higher the value for *t*), the higher the probability of a mismatch caused by mutation. This follows the evolutionary model for a given taxon, allowing us to represent diverse and conserved taxa equally well with one algorithm. If a taxon has higher mitodiversity and longer branch lengths, the model is more lenient towards substitutions. Conversely, substitutions incur a lower likelihood of a taxon’s mitogenome being highly conserved. After considering the probability of a mutation, we denote $\sum P(b_{s}\;|\;b_{g})$, where $b_{s}$ is our graph base after marginalizing over every possibility of a mutation.

Following mutation, a mismatch can be explained by a deaminated base (C→U, read by the sequencer as T, or G→A) in the fragment. The probability of observing a mismatch explained by a deamination event is given by:


$$P(b_{d}\;|\;b_{s})=\left\{ \begin{array}{cc} 1-\delta & b_{d}=b_{s},\\ \delta & (b_{d}=T\;\text{and}\;b_{s}=C,\;b_{d}=A\;\text{and}\;b_{s}=G), \end{array}\right.$$

where $b_{d}$ is our graph base after the marginalization of all possible cases of damage. *δ* depends on the base position within the fragment. The probability of a deamination event is higher at the 5’ end of the fragment for C→T substitutions and the 3’ end for G→A substitutions. We allow the user to provide damage rate matrices for their data to reflect the level of damage in their sample. The probabilities of deamination and sequencing error are independent of the tree placement. This allows us to precompute them for every alignment in the data at runtime. Again, the probability of observing either of the four bases following deamination is computed.

Following mutations and damage, we compute the probability of a sequencing error ϵ derived from the base quality reported by the sequencer and denoted by:


$$P(b\;|\;b_{d})=\left\{ \begin{array}{cc} 1-\epsilon & b=b_{d},\\ \frac{\epsilon }{3} & (b\neq b_{r}). \end{array}\right.$$

A marginalization over each of the four bases following a potential sequencing error is performed to obtain our likelihood model and the probability of *b*.

Finally, we calculate the probability of the base for these two possibilities: (i) the source is $N_{A}$ and (ii) the source is $N_{D}$. The length of the branch from $N_{A}$ to $N_{D}$ is *t*, and the relative branch placement is $\beta_{i}$, where $0\leq \beta_{i}\leq 1$. A value of $\beta_{i}=1$ would imply that we believe that the source was equal to $N_{D}$, while $\beta_{i}=0$ would mean that the source was $N_{A}$. The distance from the source to $N_{D}$ is $t_{D}=(1-\beta_{i})t$, while the distance from the source to $N_{A}$ is $t_{A}=\beta_{i}t$. We compute the product of $P(b\;|\;N_{A},t_{A})$ and $P(b\;|\;N_{D},t_{D})$ and calculate their weighted average across the entire aligned DNA fragment *r* consisting of *j* aligned bases denoted $b_{i}$:


$$P(r\;|\;\beta_{i})=(1-\beta_{i})\prod_{i=1}^{j}P(b_{i}\;|\;N_{A},t_{A})+\beta_{i}\prod_{i=1}^{j}P(b_{i}\;|\;N_{D},t_{D}).$$

The product of all reads gives us the final likelihood of a single source. The case for multiple sources is found on page 17.

### Signature Node Detection

soibean first maps the input FASTQ file to the subgraph corresponding to the taxon of interest. We count the number of aDNA fragments that align to the different signature node sets in the pangenome graph. We use the term “signature nodes” in analogy with the concept of “signature genes” in metagenomics to denote nodes in the graph only supported by one unique reference path. A signature node represents one or multiple bases in a position of the mitochondrial genome, which is unique to one species (reference genome) in the graph. We term the set of all signature nodes for a given reference sequence the “signature node set.”

Based on the total number of aligned aDNA fragments to a signature node set in the pangenome graph, we can estimate an initial number of distinct sources present in the sample. A signature node set must have a total frequency of more than 1% of the total alignments to the entire subgraph. This is implemented to reduce signature node predictions from noisy data. We set our first estimate of *k* as our maximum *k* value and run our MCMC sampling algorithm for every whole number from 0 to *k*. For a visual representation of the workflow, see Fig. 1. A detailed description of our MCMC sampling scheme as well as its diagnostics, can be found in supplementary section 2.3, Supplementary Material online.

## Declarations

### Software Versions

We used PRANK version v.170427, RAxML version 8.2.12, and FastML version 3.11. Our vg version was 1.44.0—“Solara”, SPIMAP version 1.2 (Rasmussen and Kellis 2011) and vgan version 3.0.0—Fagiolo. We used HAYSTAC version v0.4.8 and pathPhynder version 1a with BWA version 0.7.17. Our simulated data was created using gargammel version 1.1.2, ART version 2.5.8, and leeHom version 1.2.15. Additional analysis was done using SHRiMP version 2.2.2., bam2prof version 1.5.4 and schmutzi’s endoCaller version 1.5.6. All plots were produced using R version 4.3.1—“Beagle Scouts.”

## Supplementary Material

msae203_Supplementary_Data

## Data Availability

vgan can be built from source or downloaded as a static binary from https://github.com/grenaud/vgan as well as Zenodo https://doi.org/10.5281/zenodo.7875929 (Rubin et al. 2023). It is also available on BioConda https://bioconda.github.io/recipes/vgan/README.html. Database construction scripts, as well as all simulated data, are available at https://github.com/nicolaavogel/soibeanDatabase or from Zenodo https://zenodo.org/records/10828227 (Vogel 2024).
