# Supplementary material for: soibean: High-Resolution Taxonomic Identification of Ancient Environmental DNA Using Mitochondrial Pangenome Graphs
Source: Mol Biol Evol. 2024 Oct 3;41(10):msae203. doi: 10.1093/molbev/msae203 (PMC11488136; doi:10.1093/molbev/msae203)
Supplement: msae203_Supplementary_Data [file msae203_supplementary_data.pdf]

Supplementary material for “soibean: High-Resolution Taxonomic Identification of Ancient Environmental DNA Using Mitochondrial Pangenome Graphs”

Nicola Alexandra Vogel[1*], Joshua Daniel Rubin[1], Anders Gorm Pedersen[1], Peter Wad Sackett[1], Mikkel Winther Pedersen[2], Gabriel Renaud[1*]

1 Department of Health Technology, Section for Bioinformatics, Technical University of Denmark, Kongens Lyngby, Denmark

2 Centre For Ancient Environmental Genomics, Globe Institute, University of Copenhagen, Copenhagen K, Denmark.

* navo@dtu.dk, gabriel.reno@gmail.com

Contents

1 Supplementary Results **4**

1.1 Computational Performance 4

1.2 Simulation Creation 5

1.3 Benchmark 7

1.4 Benchmarking Commands 8

1.4.1 `soibean` 8

1.4.2 `HAYSTAC` 8

1.4.3 `pathPhynder - best path method` 8

1.4.4 `pathPhynder - maximum likelihood method` 9

1.5 Two Source Simulation 10

1.6 Empirical Data: Commands 11

1.6.1 `soibean` 11

1.6.2 Phylogenetic Placement 11

2 Supplementary Methods **12**

2.1 Database and Tree Construction 12

2.2 HKY Model 14

2.3 MCMC Sampling 16

2.3.1 MCMC Diagnostics 19

3 Supplementary Table **24**

4 Supplementary Figures **33**

1 Supplementary Results

1.1 Computational Performance

soibean's computational performance highly depends on the used reference graph, the size of the input FASTQ and the number of iterations specified for the MCMC. Supplementary Figure 26 shows a comparison of user time in hours for three different sizes of FASTQ input and three different numbers of iterations set for the MCMC. Each run multithreaded to 20 cores. soibean assesses the log-likelihood for the FASTQ-input in each iteration of the MCMC to take a new placement on the branch into account. Given our HKY model (see Method Section 2.2, our model needs to recompute the log-likelihood for every base of every fragment associated with the source. This is time-intensive; however, it provides us with extremely accurate estimations. Additionally, soibean can be multi-threaded, improving time performance.

We have observed that soibean's memory consumption depends primarily on the reference graph used. On average, soibean uses about 1.5Gb of memory. This varies depending on the size of the input graph.

1.2 Simulation Creation

We created six simulated samples to test `soibean`. The first sample was simulated to be a single source from an ancestral state sequence (N4) from the family of bears (Ursidae). Our ancestral states were simulated using `FastML` (Ashkenazy et al. 2012) (please find details about our database, including the ancestral state reconstruction, in the Supplementary Method Section **??**). The ancestral state we sampled from for our first simulated data set is the most recent common ancestor (MRCA) to all Black bears (*Ursus thibetanus formosanus* - NCBI accession number NC_009331.1, *Ursus thibetanus thibetanus* - NCBI accession number NC_011118.1, *Ursus thibetanus* - NCBI accession number NC_009971.1, *Ursus thibetanus ussuricus* - NCBI accession number NC_011117.1, *Ursus thibetanus mupinensis* - NCBI accession number NC_008753.1 and *Ursus americanus* - NCBI accession number NC_003426.1), including the Sun bear (*Helarctos malayanus* - NCBI accession number NC_009968.1). We sampled the N4 sequence for five different levels of coverage (500, 250, 75, 50 and 10 reads). For our two-source mixture samples, we sampled the closely related bear species ($\sim$ 98.8% genome similarity) from two Asian Black bears, namely *Ursus thibetanus formosanus* - NCBI accession number NC_009331.1 and *Ursus thibetanus thibetanus* - NCBI accession number NC_011118.1. The less closely related mixture samples ($\sim$ 93.1% genome similarity) were simulated from the Cave bear and the Brown bear mitogenome, *Ursus spelaeus* - NCBI accession number NC_011112.1 and *Ursus arctos* - NCBI accession number NC_003427.1. The distantly related two-source mixture sample ($\sim$ 83.4% genome similarity) was sim-

ulated from the American Black bear (*Ursus americanus* - NCBI accession number NC_003426.1) and the Giant Panda bear (*Ailuropoda melanoleuca* - NCBI accession number NC_009492.1). Our three-source sample was simulated from the family Saturniidae (winged insects), where we sampled from two emperor moths *Gonimbrasia belina* - NCBI accession number NC_046032.1 and *Gonimbrasia tyrrhea* - NCBI accession number NC_061326.1. The third source was sampled from the ancestral state N7 of said family. This ancestral state is the MRCA of five African moth species, namely *Nudaurelia wahlbergi* - NCBI accession number NC_061330.1, *Gonimbrasia cytherea* - NCBI accession number NC_061331.1, *Bunaea alcinoe* - NCBI accession number NC_061295.1 and the two previously named emperor moths. The last sample was simulated to contain four different sources from the family Phocidae. We simulated reads from the species *Phoca vitulia* - NCBI accession number NC_001325.1, *Phoca fasciata* - NCBI accession number NC_008428.1, *Phoca groenlandica* - NCBI accession number NC_008429.1 and *Phoca largha* - NCBI accession number NC_008430.1.

All simulated datasets were created using `gargammel`. We used the `fragSim` option to generate the wanted number of reads with a fragment length distribution following a log-normal distribution with $\mu = 3.7344$ and a $\sigma = 0.35$ as commonly seen in aDNA studies. We used `deamSim` with deamination rates taken from Günther et al. (2015) to simulate ancient damage and finally used `adptSim` to simulate adapters. We used `ART` (Huang et al. 2012) to simulate Illumina sequencing and finally used `leeHom` (Renaud et al. 2014) with ancient parameters to trim

adapters. Our mixture samples were downsampled using the `seqtk` (Shen et al. 2016) subsampling function. All simulated datasets are in our provided test data https://github.com/nicolaavogel/soibeanDatabase.

1.3 Benchmark

We benchmarked against `pathPhynder` with standard parameters (Martiniano et al. 2022) for our single-source simulations. We followed the tool's recommended guidelines and the approach taken in Kjær et al. (2022). We used `BWA ALN` (Li and Durbin 2009) for mapping to the consensus sequence. The `pathPhynder` best path results for our single-source samples are visualised in Supplementary Figures 2, 3, 4, 5, and 6. `pathPhynder`'s maximum likelihood results for the single-source samples are visualised in Supplementary Figures 7, 8, 9, 10, and 11.

To the best of our knowledge, no existing tool estimates more than one source on a phylogenetic tree. Therefore, we used `HAYSTAC` as a baseline model. The tool specialises in classifying highly similar samples (Dimopoulos et al. 2022). We used `HAYSTAC`'s standard parameters and recommended thresholds for their coverage evenness filter; additionally, we specified that a source needs at least five uniquely mapped reads to be considered present. All results are summarised in Supplementary Table 1.

1.4 Benchmarking Commands

1.4.1 soibean

```
./make_graph_files.sh [database name]

vgan soibean -fq1 [simulation.fq.gz] -t 20 --dbprefix [database name]
-o [output name]
```

1.4.2 HAYSTAC

```
haystac sample --output [sample_dir] --fastq [simulation.fq.gz]
--core 20 --trim-adapter False

haystac analyse --mode abundances --database [database]
--sample [sample_dir] --output [ouput_dir] --bowtie2-threads 20
--aDNA --cores 20
```

1.4.3 pathPhynder - best path method

```
bwa aln -l 1024 -n 0.001 -t 10 [consensus.fasta] [simulation.fq.gz]
| bwa samse [consensus.fasta] - [simulation.fq.gz] | samtools view
-F 4 -q 25 -@ 10 -uS - | samtools sort -@ 10 -o [output.sort.bam]

phynder -B -o[output.snp] [tree.newick] [input.vcf]
```

```
pathPhynder -s prepare -i [tree.newick] -p [taxa_pathphynder_tree]
-f [output.snp] -r [consensus.fasta]

pathPhynder -s all -t 10 -i [tree.newick]
-p [taxa_pathphynder_tree] -b [output.sort.bam] -r [consensus.fasta]
```

1.4.4 pathPhynder - maximum likelihood method

```
bwa aln -l 1024 -n 0.001 -t 10 [consensus.fasta] [simulation.fq.gz]
| bwa samse [consensus.fasta] - [simulation.fq.gz] | samtools view
-F 4 -q 25 -@ 10 -uS - | samtools sort -@ 10 -o [output.sort.bam]

phynder -B -o[output.snp] [tree.newick] [input.vcf]

pathPhynder -s prepare -i [tree.newick] -p [taxa_pathphynder_tree]
-f [output.snp]

Rscript R/make_vcf.R intree_folder2/ 1 [sample.vcf]

./phynder/phynder -q [output.vcf] -p 0.01 -o [query.output]
[tree.newick] [input.vcf]
```

1.5 Two Source Simulation

Additionally, we repeated this experiment with a distantly related mix of a Giant Panda bear and an American Black bear (83.4% similarity), as well as a very closely related mix of the Tibetan and Taiwan Black bear (98.8% similarity). Both mixtures were simulated in the same four proportions: $95\% - 5\%, 85\% - 15\%, 75\% - 25\%$ and $55\% - 45\%$ with an average coverage of $\sim 2.5X$ (1000 aDNA fragments) as in Pedersen et al. (2021). At this coverage, we obtain correct identification for the Giant Panda and American Black bear mixture at any proportion (Supplementary Figure 12), while we misidentify the closely related sample for the $95\% - 5\%$ and the $85\% - 15\%$ mixtures of the Asian Black bears (Supplementary Figure 13).

We repeat `soibean`'s robustness test by downsampling both mixtures to $\sim 1.3X$, $\sim 0.7X$ and $\sim 0.25X$ coverage. `soibean` can identify the mixture of the Giant Panda and the American Black bear for every mixture percentage until the lowest coverage (see Supplementary Figures 14, 15,16). For the most similar sample of Asian Black bears, we can observe that the $95\% - 5\%$ can never be identified, while the rest can be placed on the correct tree branch (see Supplementary Figures 20, 21 and 22), when downsampling.

1.6 Empirical Data: Commands

1.6.1 soibean

```
./make_graph_files.sh [database name]

vgan soibean -fq1 [input.fastq] --dbprefix [database name] -t 20
-o [output name] -k 3
```

1.6.2 Phylogenetic Placement

```
SHRiMP_2_2_2/bin/gmapper -N 4 -o 1 --single-best-mapping --sam-unaligned
--fastq --sam --no-qv-check  --qv-offset 33  [input.fastq] [reference.fa]
| samtools view -bS  -F4 /dev/stdin | samtools fillmd -b  /dev/stdin
[reference.fa] | samtools sort /dev/stdin > [output.bam]
samtools index  [output.bam]

bam2prof -double  -5p [output_5p.prof] -3p [output_3p.prof] [output.bam]

endoCaller -seq [output.fa] -log [output.log] -name [name] -deam5p [output_5p.pro[illegible]
-deam3p [output_3p.prof] [reference.fa] [output.bam]

cat [output.fa] [phylogeneticSeqs.fa] > [output_all.fa]

prank -d=[output_all.fa] -o=[output_all.prank] -showall -DNA
```

```
RAxML-8.2.12/raxmlHPC-PTHREADS -s [output_all.prank].best.fas --HKY85
-m GTRGAMMA -n [output_prefix] -w [output_dir] -p 76 -T 40 -d
```

2 Supplementary Methods

2.1 Database and Tree Construction

The database for `soibean` uses the same taxa introduced in the database for `euka` (Vogel et al. 2023), which comprise 335 different taxa of tetrapodic and arthropodic eukaryotes (the taxon Galloanserae has been resorted since `euka`'s latest release). We used the same reference genomes as input to construct a multiple sequence alignment using `PRANK` version v.170427 (Löytynoja 2014), from which we constructed a phylogenetic tree using `RAxML` version 8.2.12 with the Hasegawa Kishino Yano (HKY) substitution model (Hasegawa et al. 1985) (`--HKY85`) and estimated base frequencies (Stamatakis 2014). The phylogenetic tree is used to infer the ancestral states for each taxon using `FastML` version 3.11 with the `-mr` flag for mitochondrial genomes and the `-mh` flag to use the HKY model (Ashkenazy et al. 2012). We use the HKY substitution model for our guide tree as it allows for both nucleotide frequencies and the transition/transversion ratio to vary (Hasegawa et al. 1985). These features are crucial for the estimation of our guide tree and fitting our model because the transition/transversion ratio is much higher in mtDNA than in the autosomes (Tamura 1992) and because mtDNA is guanine-enriched on the heavy

strand to aid in stabilising RNA transcription (Wallace 1994; Levinstein Hallak et al. 2018). The HKY model allowed us to represent the variety of taxa used accurately. `FastML` outputs a multiple sequence alignment (MSA), including reconstructed ancestral states and the maximum-likelihood phylogenetic tree. We build a pangenome graph for each taxon from the new MSA with the added ancestral state sequences via the `vg construct -M` subcommand from the `vg` toolkit version 1.44.0 (Garrison et al. 2018) (Supplementary Figure 1), resulting in 326 subgraphs (i.e. connected components), each subgraph corresponding to a different taxon for `euka/soibean`. A pangenome graph is defined as $G = (N, E, H)$, where N are the nodes, E the edges, and H the graph's embedded reference paths. Each node has a unique numerical identifier called node ID, which is internally assigned by `vg`. For a detailed overview of pangenome graphs applied to animal mitogenomes, see the Supplementary Material from Vogel et al. (2023). Each path in the graph corresponds to a mitogenome in the MSA and, therefore, a node in the phylogenetic tree. Inferred ancestral genomes correspond to internal nodes, whereas the mitogenomes used as input to the MSA are leaves in the phylogenetic tree. We merged connected components for each pangenomic graph into a single set of multiple independent graphs using the `vg ids` subcommand (Garrison et al. 2018). This resulted in a combined graph with $|N| = 6889846$, $|E| = 10880449$ and $|H| = 10992$. We updated each taxon's start and end node ID for our graph index and additionally empirically computed the base frequencies for each taxon to be used for our HKY-based likelihood model. A complete overview of the database construction and all scripts used can be found https://github.com/nicolaavogel/soibeanDatabase.

2.2 HKY Model

We are interested in finding the most likely placement of ancient species on a pre-computed mitochondrial phylogenetic tree. To achieve this we use the HKY substitution model to compute the probability of an observed, ancient DNA sequence, arising by substitutions occurring along a branch of the tree, some distance from one of the existing nodes.

The instantaneous rate matrix for the HKY model can be written as (Hillis et al. 1996; Xia 2017):

$$\boldsymbol{Q} = \mu \begin{bmatrix} - & \pi_C & \kappa\pi_G & \pi_T \\ \pi_A & - & \pi_G & \kappa\pi_T \\ \kappa\pi_A & \pi_C & - & \pi_T \\ \pi_A & \kappa\pi_C & \pi_G & - \end{bmatrix}$$

The rows and columns of $\boldsymbol{Q}$ correspond to the nucleotides A, C, G, and T in that order. An element, Q_{ij}, gives the rate at which nucleotide i changes to nucleotide j, and values are in units of substitutions per site per time unit. Elements on the diagonal (not shown here) are set such that all rows sum to zero: $Q_{ii} = -\sum_{j \neq i} Q_{ij}$. In the expressions above κ is the ratio between the rates of transition-type and transversion-type substitutions, while π_A, π_C, π_G, and π_T are the equilibrium nucleotide frequencies. The multiplicative factor μ will, in part, depend on the units of time used in the matrix (e.g., rates measured per century will be 100 times larger

than rates measured per year).

Given the rate matrix $\boldsymbol{Q}$ it is possible to compute the transition probability matrix $\boldsymbol{P}(t)$ for a given branch length t: $\boldsymbol{P}(t) = e^{\boldsymbol{Q}t}$. In this expression, t has to be expressed in the same time units as $\boldsymbol{Q}$. Since branch lengths in the pre-computed phylogeny are expressed as the expected change per site (and not in some unit of time), we first normalise $\boldsymbol{Q}$ correspondingly. Specifically, this is done by setting μ such that the expected rate of change, ρ, is 1: If $\rho = 1$ then the expected amount of change on a branch of length t will be: $\nu = \rho t = t$. This is equivalent to having chosen a unit of time such that the expected change on a branch (ν, measured in substitutions per site) is numerically identical to the branch length (t, measured in those time units).

The expected rate of change for the HKY model is (Hillis et al. 1996):

$$\begin{aligned}\rho &= \sum_i \pi_i \sum_{j \neq i} Q_{ij} \\ &= 2(\kappa\pi_A\pi_G + \kappa\pi_C\pi_T + \pi_A\pi_C + \pi_A\pi_T + \pi_C\pi_G + \pi_G\pi_T)\mu\end{aligned}$$

Setting this equal to 1, and isolating μ, we find:

$$\mu = \frac{1}{2(\kappa\pi_A\pi_G + \kappa\pi_C\pi_T + \pi_A\pi_C + \pi_A\pi_T + \pi_G\pi_C + \pi_G\pi_T)}$$

Using $\boldsymbol{P}(t) = e^{\boldsymbol{Q}t}$ one can derive the following expressions for the probability of observing nucleotide b, in an ancient taxon, given the presence of the nucleotide r, at a distance of t, in a node on the tree:

$$\begin{cases} \pi_r + \pi_r(\frac{1}{\Pi_r} - 1)e^{-\mu t} + (\frac{\Pi_r - \pi_r}{\Pi_r})e^{\mu t A} & (b = r), \\ \pi_r + \pi_r(\frac{1}{\Pi_r} - 1)e^{-\mu t} - (\frac{\pi_r}{\Pi_r})e^{-\mu t A} & (b \neq r, \text{transition}), \\ \pi_r(1 - e^{-\mu t}) & (b \neq r, \text{transversion}) \end{cases}$$

Here $A = 1 + \Pi_r(\kappa - 1)$, with $\Pi_r = \pi_A + \pi_G$ (if r is A or G) and $\Pi_r = \pi_C + \pi_T$ (if r is C or T). The branch length t is the distance between the ancient taxon and a nearby node on the tree and is expressed in units of expected change as explained above. Based on previous analyses of human mitochondrial evolution, we set κ to 22 (Levinstein Hallak et al. 2018). The equilibrium nucleotide frequencies are set to their empirical values in the data set at hand. The value of μ is computed from κ and $(\pi_A, \pi_C, \pi_G, \pi_T)$ according to the expression above.

2.3 MCMC Sampling

The MCMC process is run k times, corresponding to the number of significant signature node paths identified (or alternatively, the number specified by the user if one is provided). For each MCMC run, four chains are deployed: the first chain is initialized at the identified signature nodes, whereas the subsequent three incorporate random nodes from the tree as starting points. The initial run of the MCMC ($k = 1$) initializes placement from the most prevalent signature node path. Subsequent runs incrementally include the next most frequent paths as starting points, continuing this

pattern until k equals the total count of significant signature node paths. The initial proportion of each source is set at $1/k$, and the initial branch position is sampled uniformly from $[0, 1]$, where 0 signifies the ancestral state, and 1 is the derived state of the branch.

We use a Metropolis-Hastings algorithm (Metropolis et al. 1953; Hastings 1970) for our MCMC sampling. Throughout the MCMC runs, new branch placements and source proportions are sampled. The branches are normalised to unit length t, with the N_A marked as 0 and the N_D as 1. Proposed distances d to new placements $\boldsymbol{\beta}^*$ are drawn from a normal distribution, with a mean of 0 and the standard deviation linearly decreasing from 3 to 0.1 throughout the burn-in phase, then from 0.1 to $1e^{-10}$ in subsequent iterations. Should a proposed distance be negative, the transition is towards the root; conversely, a positive distance indicates a movement towards the leaves.

If the sum of our current position on the tree branch and the newly sampled distance is smaller than one ($d + \beta_i < 1$) or the difference between the current position and the sampled distance is higher than 0 ($d - \beta_i > 0$), we update our current position on the branch to the updated position on the branch ($\beta_{i \pm d}$). If the sum of our current position on the tree branch and the newly sampled distance is larger than 1 ($d + \beta_i > 1$), we move to either of the two child branches of N_D equiprobably. In case N_D is a leaf node, we sample a new move. When the difference between the current position on the tree branch and the sampled distance is negative ($d - \beta_i > 0$)

we move to either the parent branch or the sibling branch of N_A equiprobably. If N_A is the root node, we sample a new move (see Figure **??**).

If $k \neq 1$, we simultaneously sample a new proportion vector $\boldsymbol{\theta}^*$. Every proportion θ_i^* is sampled from $N(\theta_i, 0.1)$ and $\theta_i^* \in [0, 1]$. We use the sum of $\boldsymbol{\theta}^*$ to normalise each θ_i^* so that $\sum \boldsymbol{\theta}^* = 1$.

We calculate the new likelihood for $P(D|\boldsymbol{\beta}^*, \boldsymbol{\theta}^*)$. If $P(D|\boldsymbol{\beta}^*, \boldsymbol{\theta}^*) > P(D|\boldsymbol{\beta}, \boldsymbol{\theta})$ we accept the move, append the new parameter values to the sample file, and continue to the next iteration. If $P(D|\boldsymbol{\beta}^*, \boldsymbol{\theta}^*) < P(D|\boldsymbol{\beta}, \boldsymbol{\theta})$ we accept the move with probability $\frac{P(D|\boldsymbol{\beta}^*,\boldsymbol{\theta}^*)}{P(D|\boldsymbol{\beta},\boldsymbol{\theta})} \frac{q(\boldsymbol{\beta},\boldsymbol{\theta}|\boldsymbol{\beta}^*,\boldsymbol{\theta}^*)}{q(\boldsymbol{\beta}^*,\boldsymbol{\theta}^*|\boldsymbol{\beta},\boldsymbol{\theta})}$. Here q is the proposal distribution for moves in parameter space, and $q(X|Y)$ is the probability of proposing X when the current parameter values are Y. If this distribution is symmetric, i.e. $q(X|Y) = q(Y|X)$, the q-terms cancel.

We present a quick argument that the proposal sampling distribution is symmetric. In other words, we aim to argue that for Markov states X and Y in our parameter space, sampling Y from state X has a probability equal to that of sampling X from state Y. To see this, recall that in our sampling scheme, we first fix a distance to move and a direction (ancestral or derived) in which to move that distance. Note also that for a given proposal move, we do not allow any self-intersecting paths on the tree. Therefore, given the bifurcating nature of tree topologies, there will always only be one possible way to sample Y from X for any Markov states X, Y. Along

this path, there will be some number of bifurcations in which one of the two possible forks will be followed. The probability of taking any given fork is $\frac{1}{2}$. Since there are the same number of forks on the unique path from Y to X as there are from X to Y, it clearly follows that the probability of sampling X from Y is identical to that of sampling Y from X.

By default, we perform 500,000 MCMC iterations and discard the first 75,000 iterations as burn-in. These values can be overridden by the user via command-line options. Notably, lower values of k should require a lower number of iterations for convergence.

2.3.1 MCMC Diagnostics

After inference is complete, it is important to assess whether the chains managed to converge to the true underlying posterior distribution. This can be assessed individually for each parameter of the model. To assess the convergence of phylogenetic placement, we keep track of the patristic distance from the accepted position to all the leaves on the tree (the patristic distance is the distance along the tree between two nodes, i.e., the sum of lengths of the branches on the path between those nodes (Fourment and Gibbs 2006)). Specifically, we compute a vector where the elements are the patristic distances from the accepted position to each of the leaf nodes. We also compute a vector giving the patristic distances from the *root node* to each of the leaves. This is done for every one hundred iterations after the burn-in period. We

then compute the Euclidean distance between these two vectors of patristic distances. If a trace plot of this Euclidean distance against MCMC iteration shows any upward or downward trend, then that would indicate a lack of convergence. If phylogenetic placement *has* converged then the trace plot should move around a constant value. We also provide the user with the chain statistics, including the mean, median, 95% credible intervals, variance, autocorrelation, and effective sample size (ESS) for each chain and each estimated parameter. We warn the user if the ESS for a chain is below 200 (Martino et al. 2017), as it could mean the estimates are insufficiently precise.

Another important and widely-used metric for assessing convergence is called $\hat{R}$. We compute $\hat{R}$ for both $\boldsymbol{\theta}$ and $\boldsymbol{\beta}$ across chains. The measure essentially compares the within-chain variance with the between-chain variance; a value close to 1 indicates convergence (Gelman and Rubin 1992; Brooks and Gelman 1998). Values larger than 1.05 (Vehtari et al. 2021) may indicate non-converging chains.

References

Ashkenazy, H. et al. (2012). "FastML: a web server for probabilistic reconstruction of ancestral sequences". In: *Nucleic acids research* 40.W1, W580–W584.

Brooks, S. P. and Gelman, A. (1998). "General methods for monitoring convergence of iterative simulations". In: *Journal of computational and graphical statistics* 7.4, pp. 434–455.

Dimopoulos, E. A. et al. (2022). "HAYSTAC: A Bayesian framework for robust and rapid species identification in high-throughput sequencing data". In: *PLOS Computational Biology* 18.9, e1010493.

Fourment, M. and Gibbs, M. J. (2006). "PATRISTIC: a program for calculating patristic distances and graphically comparing the components of genetic change". In: *BMC evolutionary biology* 6, pp. 1–5.

Garrison, E. et al. (2018). "Variation graph toolkit improves read mapping by representing genetic variation in the reference". In: *Nature biotechnology* 36.9, pp. 875–879.

Gelman, A. and Rubin, D. B. (1992). "Inference from iterative simulation using multiple sequences". In: *Statistical science* 7.4, pp. 457–472.

Günther, T. et al. (2015). "Ancient genomes link early farmers from Atapuerca in Spain to modern-day Basques". In: *Proceedings of the National Academy of Sciences* 112.38, pp. 11917–11922.

Hasegawa, M., Kishino, H., and Yano, T.-a. (1985). "Dating of the human-ape splitting by a molecular clock of mitochondrial DNA". In: *Journal of molecular evolution* 22, pp. 160–174.

Hastings, W. K. (1970). "Monte Carlo sampling methods using Markov chains and their applications". In: *Biometrika*.

Hillis, D. M., Moritz, C., and Mable, B. K. (1996). *Molecular systematics*. Vol. 23. Sinauer.

Huang, W. et al. (2012). "ART: a next-generation sequencing read simulator". In: *Bioinformatics* 28.4, pp. 593–594.

Kjær, K. H. et al. (2022). "A 2-million-year-old ecosystem in Greenland uncovered by environmental DNA". In: *Nature* 612.7939, pp. 283–291.

Levinstein Hallak, K., Tzur, S., and Rosset, S. (2018). "Big data analysis of human mitochondrial DNA substitution models: a regression approach". In: *BMC genomics* 19.1, pp. 1–13.

Li, H. and Durbin, R. (2009). "Fast and accurate short read alignment with Burrows–Wheeler transform". In: *bioinformatics* 25.14, pp. 1754–1760.

Löytynoja, A. (2014). "Phylogeny-aware alignment with PRANK". In: *Multiple sequence alignment methods*, pp. 155–170.

Martiniano, R. et al. (2022). "Placing ancient DNA sequences into reference phylogenies". In: *Molecular biology and evolution* 39.2, msac017.

Martino, L., Elvira, V., and Louzada, F. (2017). "Effective sample size for importance sampling based on discrepancy measures". In: *Signal Processing* 131, pp. 386–401.

Metropolis, N. et al. (1953). “Equation of state calculations by fast computing machines”. In: *The journal of chemical physics* 21.6, pp. 1087–1092.

Pedersen, M. W. et al. (2021). “Environmental genomics of Late Pleistocene black bears and giant short-faced bears”. In: *Current Biology* 31.12, pp. 2728–2736.

Renaud, G., Stenzel, U., and Kelso, J. (2014). “leeHom: adaptor trimming and merging for Illumina sequencing reads”. In: *Nucleic acids research* 42.18, e141–e141.

Shen, W. et al. (2016). “SeqKit: a cross-platform and ultrafast toolkit for FASTA/Q file manipulation”. In: *PloS one* 11.10, e0163962.

Stamatakis, A. (2014). “RAxML version 8: a tool for phylogenetic analysis and post-analysis of large phylogenies”. In: *Bioinformatics* 30.9, pp. 1312–1313.

Tamura, K. (1992). “The rate and pattern of nucleotide substitution in Drosophila mitochondrial DNA.” In: *Molecular biology and evolution* 9.5, pp. 814–825.

Vehtari, A. et al. (2021). “Rank-normalization, folding, and localization: An improved $\hat{R}$ for assessing convergence of MCMC (with discussion)”. In: *Bayesian analysis* 16.2, pp. 667–718.

Vogel, N. A. et al. (2023). “euka: Robust tetrapodic and arthropodic taxa detection from modern and ancient environmental DNA using pangenomic reference graphs”. In: *Methods in Ecology and Evolution.*

Wallace, D. C. (1994). “Mitochondrial DNA mutations in diseases of energy metabolism”. In: *Journal of bioenergetics and biomembranes* 26, pp. 241–250.

Xia, X. (2017). “Deriving transition probabilities and evolutionary distances from substitution rate matrix by probability reasoning”. In: *J Genet Genome Res* 3.031.

3 Supplementary Table

Reads	Average coverage	Proportion	Simulated Source(s)	Predicted Source(s)
500	1.3X	1	Ancestral state *N4*	*U. americanus*<br>*M. ursinus*<br>*N13*<br>*U. spelaeus*<br>*U. arctos*
250	0.66X	1	Ancestral state *N4*	*H. malayanus*<br>*U. americanus*<br>*M. ursinus*
75	0.2X	1	Ancestral state *N4*	*U. americanus*
50	0.13X	1	Ancestral state *N4*	None
10	0.026X	1	Ancestral state *N4*	None
1000	2.6X	55-45	*U. thibetanus formosanus*<br>*U. thibetanus thibetanus*	*U. thibetanus formosanus*<br>*U. thibetanus thibetanus*
500	1.3X	55-45	*U. thibetanus formosanus*<br>*U. thibetanus thibetanus*	*U. thibetanus formosanus*<br>*U. thibetanus thibetanus*
250	0.66X	55-45	*U. thibetanus formosanus*<br>*U. thibetanus thibetanus*	*U. thibetanus formosanus*<br>*U. thibetanus thibetanus*

100	0.26X	55-45	*U. thibetanus formosanus* *U. thibetanus thibetanus*	*U. thibetanus thibetanus*
1000	2.6X	55-45	*U. spelaeus* *U. arctos*	*U. spelaeus* *U. arctos*
500	1.3X	55-45	*U. spelaeus* *U. arctos*	*U. spelaeus* *U. arctos*
250	0.66X	55-45	*U. spelaeus* *U. arctos*	*U. spelaeus* *U. arctos*
100	0.26X	55-45	*U. spelaeus* *U. arctos*	*U. spelaeus* *U. arctos*
1000	2.6X	55-45	*A. melanoleuca* *U. americanus*	*A. melanoleuca* *U. americanus*
500	1.3X	55-45	*A. melanoleuca* *U. americanus*	*A. melanoleuca* *U. americanus*
250	0.66X	55-45	*A. melanoleuca* *U. americanus*	*A. melanoleuca* *U. americanus*
100	0.26X	55-45	*A. melanoleuca* *U. americanus*	*A. melanoleuca* *U. americanus*

1000	2.6X	75-25	*U. thibetanus formosanus U. thibetanus thibetanus*	*U. thibetanus formosanus U. thibetanus thibetanus*
500	1.3X	75-25	*U. thibetanus formosanus U. thibetanus thibetanus*	*U. thibetanus formosanus U. thibetanus thibetanus*
250	0.66X	75-25	*U. thibetanus formosanus U. thibetanus thibetanus*	*U. thibetanus formosanus U. thibetanus thibetanus*
100	0.26X	75-25	*U. thibetanus formosanus U. thibetanus thibetanus*	*U. thibetanus formosanus*
1000	2.6X	75-25	*U. spelaeus U. arctos*	*U. spelaeus U. arctos*
500	1.3X	75-25	*U. spelaeus U. arctos*	*U. spelaeus U. arctos*
250	0.66X	75-25	*U. spelaeus U. arctos*	*U. spelaeus U. arctos*
100	0.26X	75-25	*U. spelaeus U. arctos*	*U. spelaeus U. arctos*
1000	2.6X	75-25	*A. melanoleuca U. americanus*	*A. melanoleuca U. americanus*

500	1.3X	75-25	*A. melanoleuca U. americanus*	*A. melanoleuca U. americanus*
250	0.66X	75-25	*A. melanoleuca U. americanus*	*A. melanoleuca U. americanus*
100	0.26X	75-25	*A. melanoleuca U. americanus*	*A. melanoleuca U. americanus*
1000	2.6X	85-15	*U. thibetanus formosanus U. thibetanus thibetanus*	*U. thibetanus formosanus U. thibetanus thibetanus*
500	1.3X	85-15	*U. thibetanus formosanus U. thibetanus thibetanus*	*U. thibetanus formosanus U. thibetanus thibetanus*
250	0.66X	85-15	*U. thibetanus formosanus U. thibetanus thibetanus*	*U. thibetanus formosanus*
100	0.26X	85-15	*U. thibetanus formosanus U. thibetanus thibetanus*	*U. thibetanus formosanus*

1000	2.6X	85-15	*U. spelaeus* *U. arctos*	*U. spelaeus* *U. arctos*
500	1.3X	85-15	*U. spelaeus* *U. arctos*	*U. spelaeus* *U. arctos*
250	0.66X	85-15	*U. spelaeus* *U. arctos*	*U. spelaeus*
100	0.26X	85-15	*U. spelaeus* *U. arctos*	*U. spelaeus*
1000	2.6X	85-15	*A. melanoleuca* *U. americanus*	*A. melanoleuca* *U. americanus*
500	1.3X	85-15	*A. melanoleuca* *U. americanus*	*A. melanoleuca* *U. americanus*
250	0.66X	85-15	*A. melanoleuca* *U. americanus*	*A. melanoleuca*
100	0.26X	85-15	*A. melanoleuca* *U. americanus*	*A. melanoleuca* *U. americanus*

1000	2.6X	95-5	*U. thibetanus formosanus*<br>*U. thibetanus thibetanus*<br>*M. ursinus*<br>*A. melanoleuca*	*U. thibetanus formosanus*<br>*U. thibetanus thibetanus*<br>*M. ursinus*<br>*A. melanoleuca*
500	1.3X	95-5	*U. thibetanus formosanus*<br>*U. thibetanus thibetanus*	*U. thibetanus formosanus*
250	0.66X	95-5	*U. thibetanus formosanus*<br>*U. thibetanus thibetanus*	*U. thibetanus formosanus*
100	0.26X	95-5	*U. thibetanus formosanus*<br>*U. thibetanus thibetanus*	*U. thibetanus formosanus*
1000	2.6X	95-5	*U. spelaeus*<br>*U. arctos*	*U. spelaeus*<br>*U. arctos*
500	1.3X	95-5	*U. spelaeus*<br>*U. arctos*	*U. spelaeus*<br>*U. arctos*
250	0.66X	95-5	*U. spelaeus*<br>*U. arctos*	*U. spelaeus*
100	0.26X	95-5	*U. spelaeus*<br>*U. arctos*	*U. spelaeus*

1000	2.6X	95-5	*A. melanoleuca U. americanus*	*A. melanoleuca U. americanus*
500	1.3X	95-5	*A. melanoleuca U. americanus*	*A. melanoleuca U. americanus*
250	0.66X	95-5	*A. melanoleuca U. americanus*	*A. melanoleuca U. americanus*
100	0.26X	95-5	*A. melanoleuca U. americanus*	*U. americanus*
1500	4X	47-33-20	*G. tyrrhea G. belina N7*	*G. tyrrhea G. belina G. maja B. alcinoe G. cytherea N. wahlbergi*
750	2X	47-33-20	*G. tyrrhea G. belina N7*	*G. tyrrhea G. belina B. alcinoe G. maja G. cytherea*

375	1X	47-33-20	*G. tyrrhea*<br>*G. belina*<br>*N7*	*G. tyrrhea*<br>*G. belina*<br>*B. alcinoe*
150	0.4X	47-33-20	*G. tyrrhea*<br>*G. belina*<br>*N7*	*G. tyrrhea*<br>*G. belina*
2000	5.2X	25-25-25-25	*P. fasciata*<br>*P. groen-landica*<br>*P. vitulina*<br>*P. largha*	*P. fasciata*<br>*P. groen-landica*<br>*P. vitulina*<br>*P. largha*
1000	2.6X	25-25-25-25	*P. fasciata*<br>*P. groen-landica*<br>*P. vitulina*<br>*P. largha*	*P. fasciata*<br>*P. groen-landica*<br>*P. vitulina*<br>*P. largha*
500	1.3X	25-25-25-25	*P. fasciata*<br>*P. groen-landica*<br>*P. vitulina*<br>*P. largha*	*P. fasciata*<br>*P. groen-landica*<br>*P. vitulina*<br>*P. largha*
200	0.55X	25-25-25-25	*P. fasciata*<br>*P. groen-landica*<br>*P. vitulina*<br>*P. largha*	*P. fasciata*<br>*P. groen-landica*<br>*P. vitulina*

Table 1: Results table for the baseline model `HAYSTAC` for every simulated test.

4 Supplementary Figures

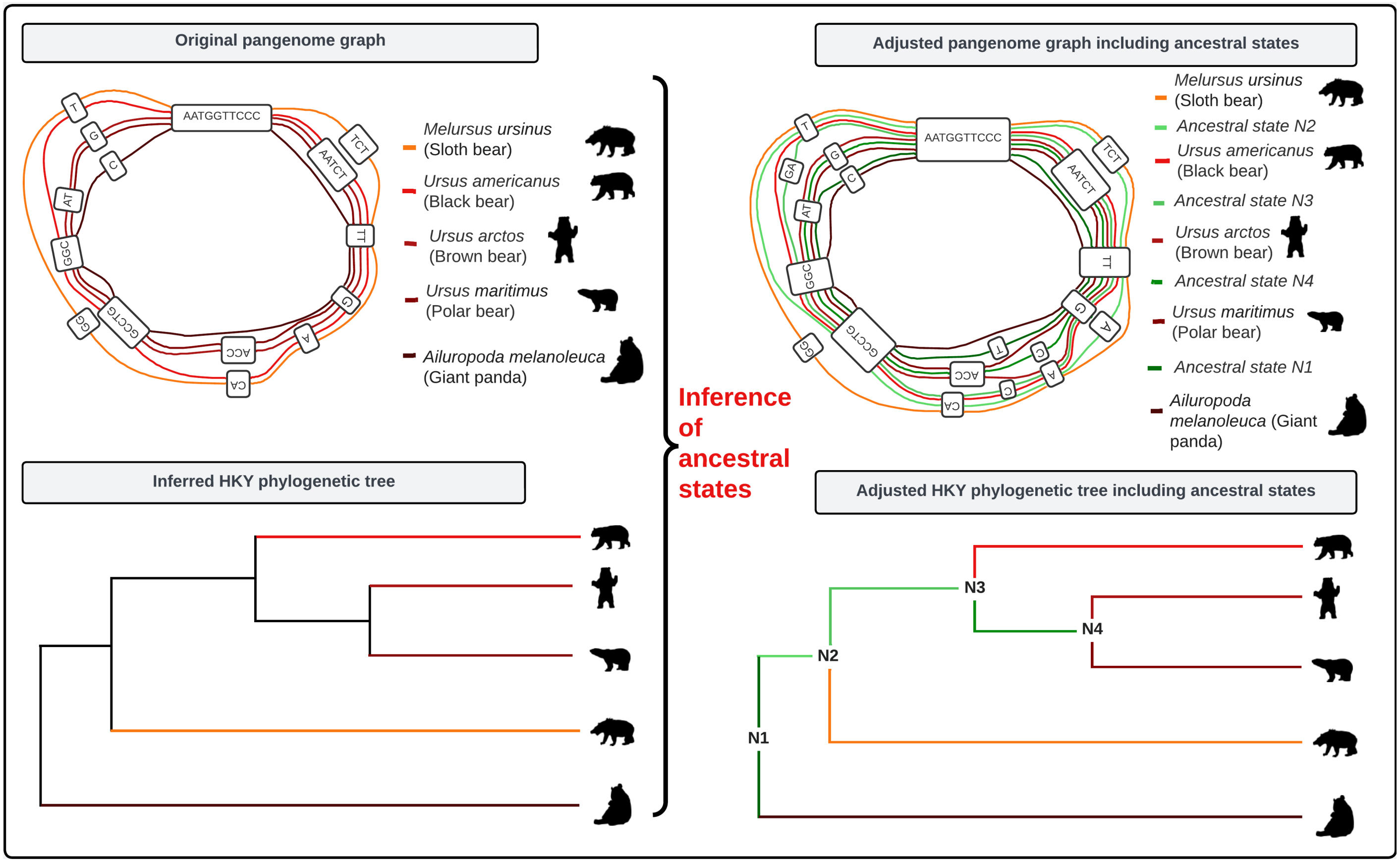

Figure 1: Workflow of a pangenome graph including ancestral states. The figure shows the original pangenome graph, including their phylogenetic tree, and follows with an adjusted pangenome graph, where ancestral state sequences are included and the corresponding phylogenetic tree.

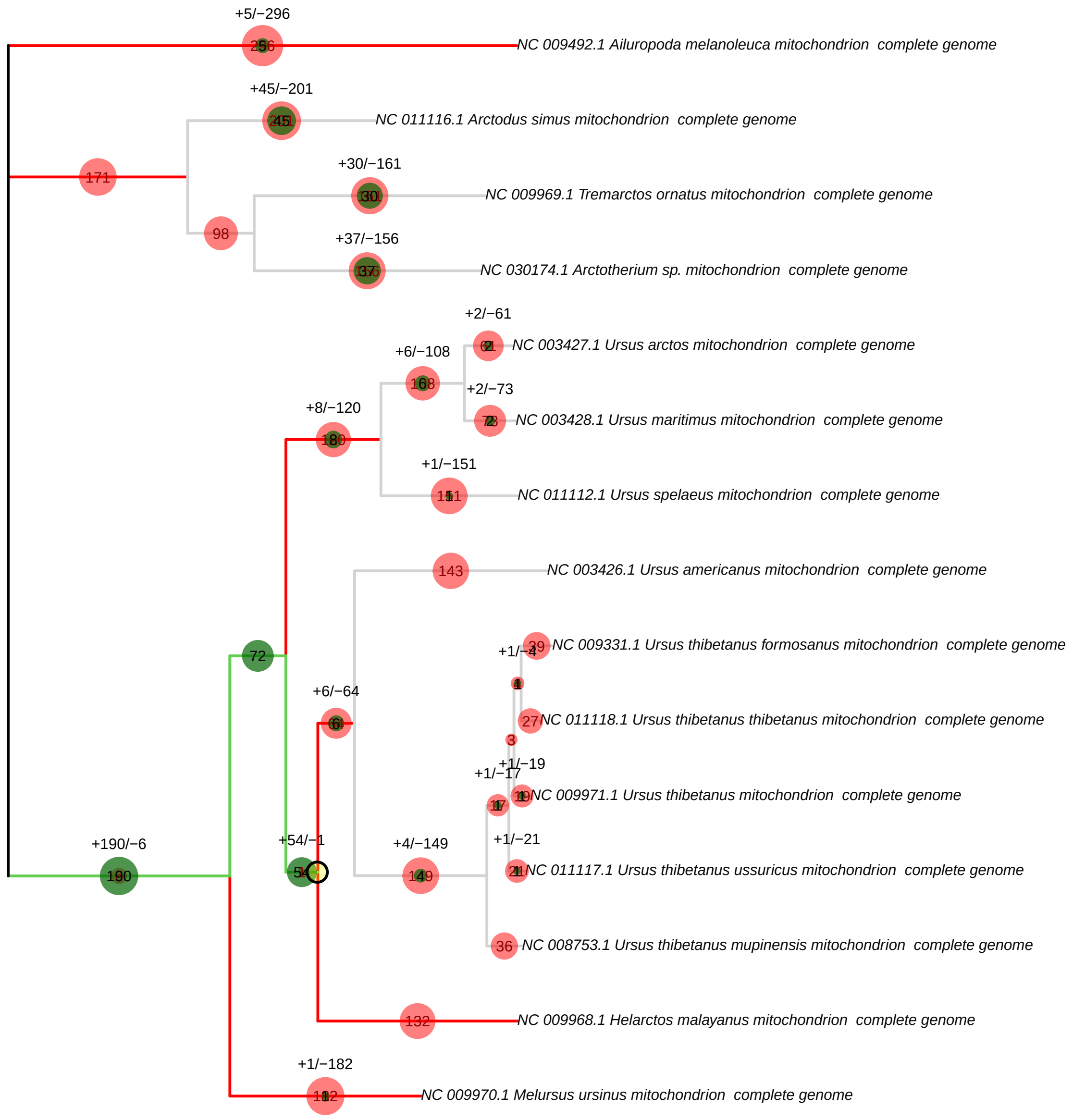

Figure 2: `pathPhynder` best path method results for the simulated ancient single-source sample N4 at $\sim 1.3x$ coverage. `pathPhynder` shows the best path for the given sample, which ends at the correct ancestral node N4.

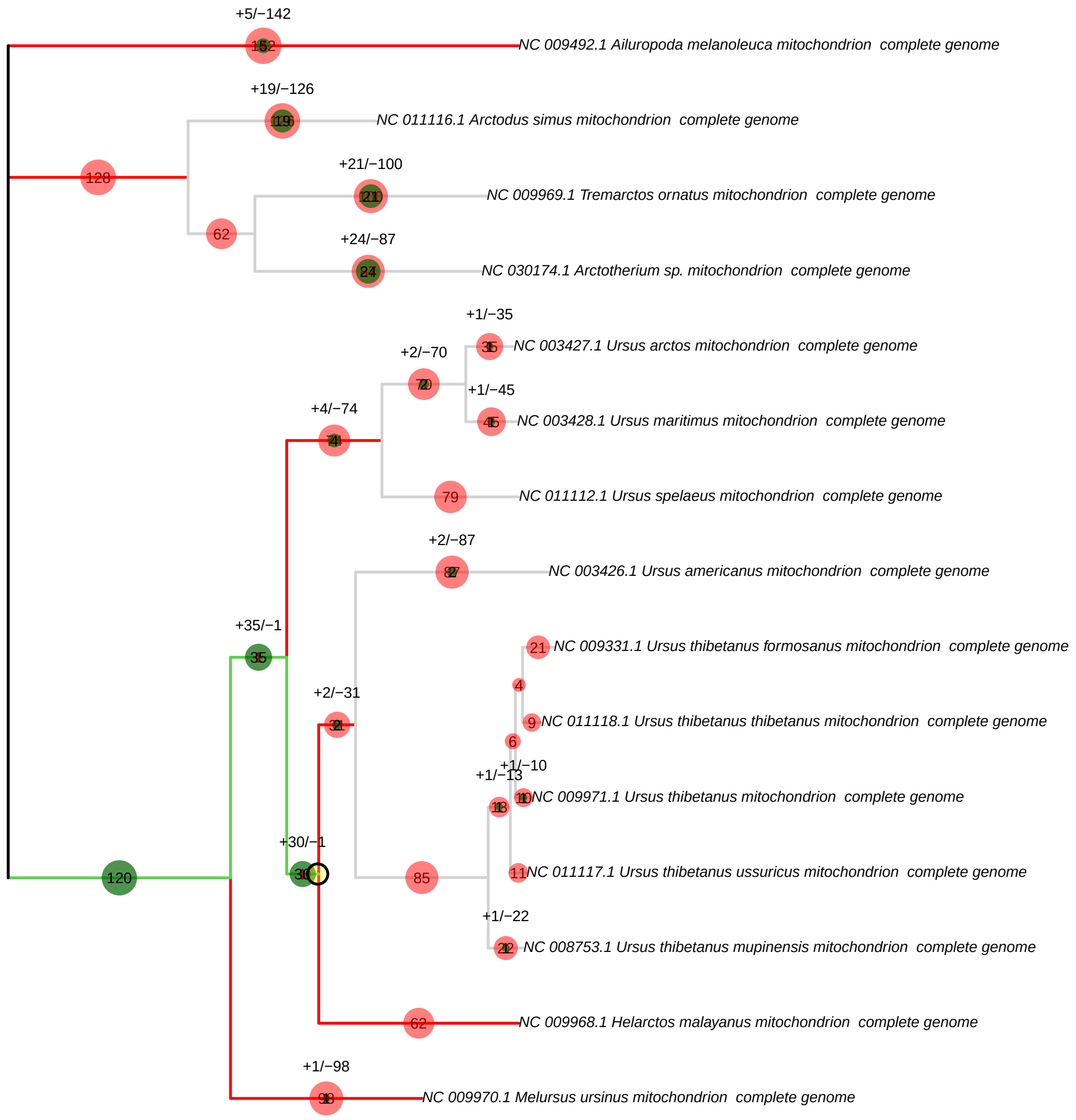

Figure 3: `pathPhynder` best path method results for the simulated ancient single-source sample N4 at $\sim 0.66X$ coverage. `pathPhynder` shows the best path for the given sample, which ends at the correct ancestral node N4.

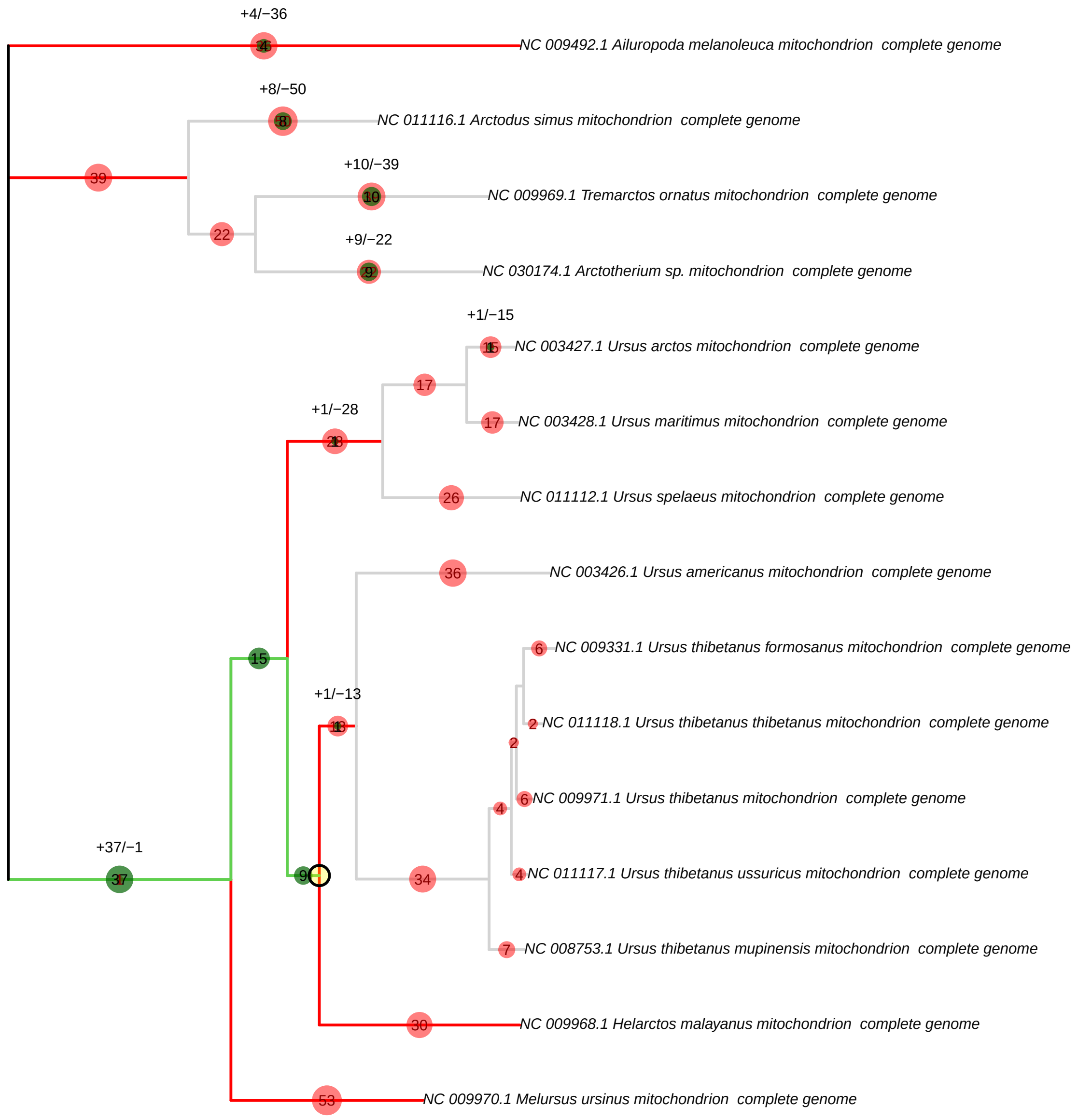

Figure 4: `pathPhynder` best path method results for the simulated ancient single-source sample N4 at $\sim 0.2X$ coverage. `pathPhynder` shows the best path for the given sample, which ends at the correct ancestral node N4.

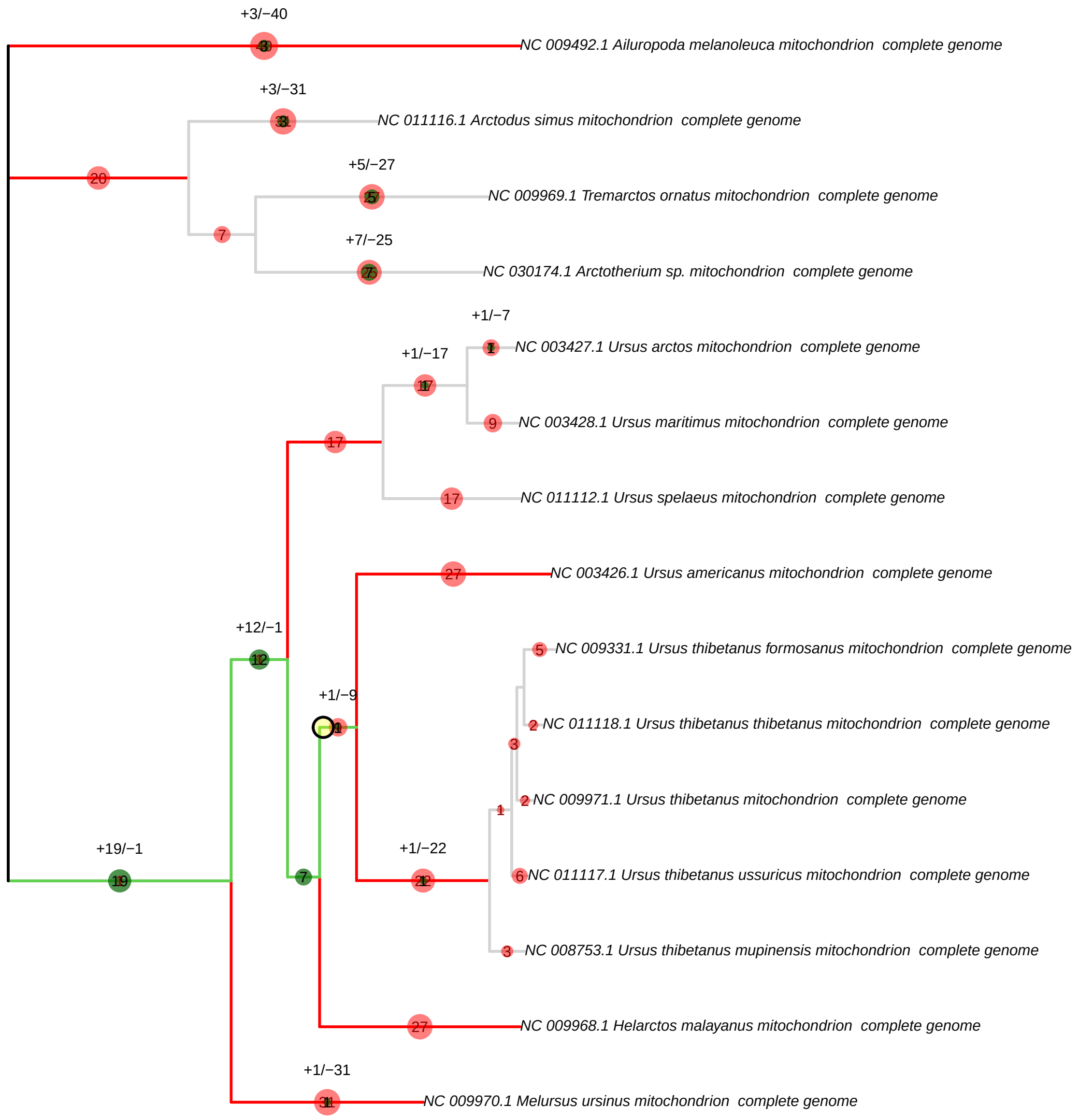

Figure 5: `pathPhynder` best path method results for the simulated ancient single-source sample N4 at $\sim 0.13X$ coverage. `pathPhynder` shows the best path for the given sample, which ends at one ancestral node after the target one N4 at N5.

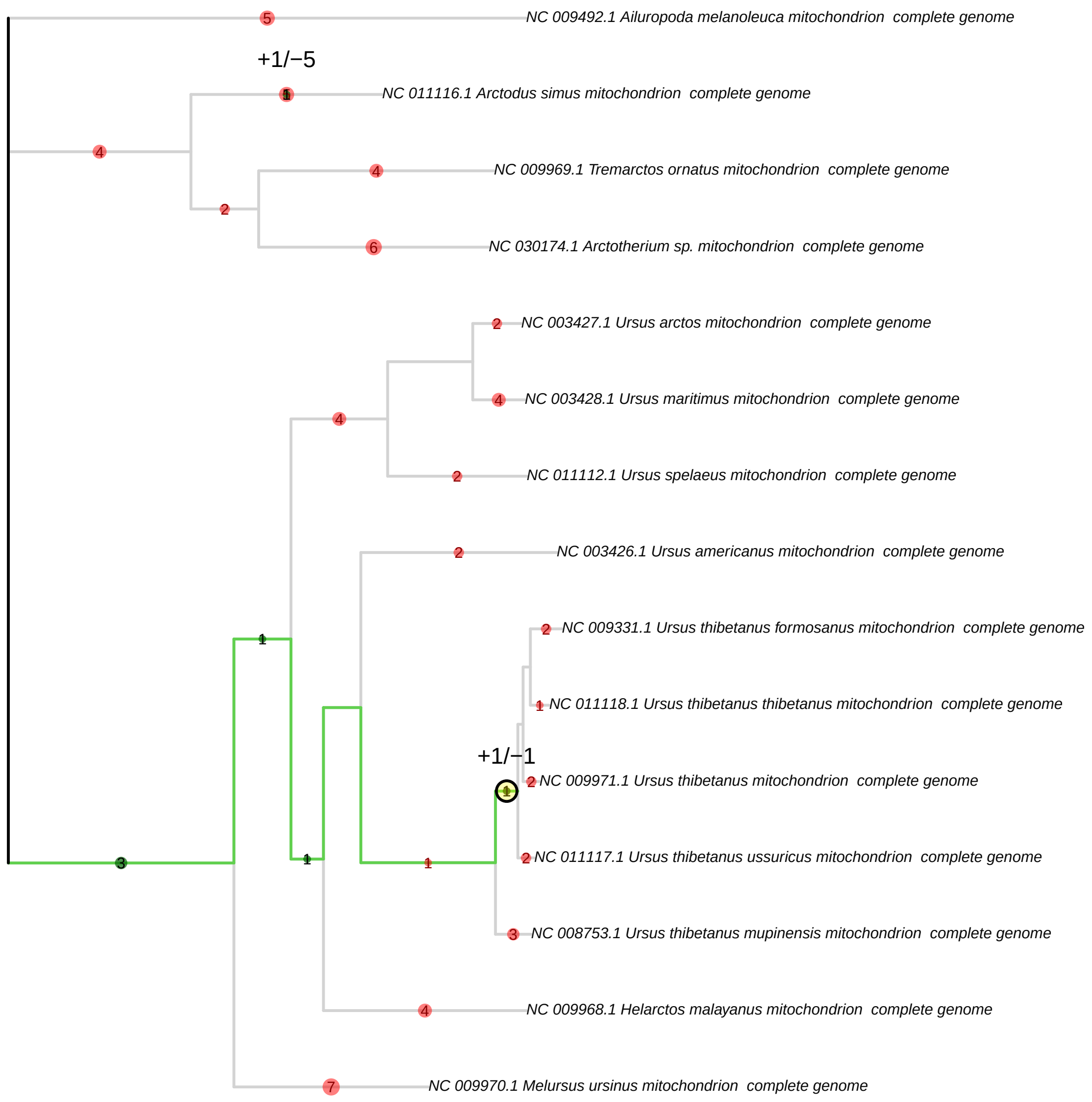

Figure 6: `pathPhynder` best path method results for the simulated ancient single-source sample N4 at $\sim 0.026X$ coverage. `pathPhynder` shows the best path for the given sample, which ends at the ancestral node N6.

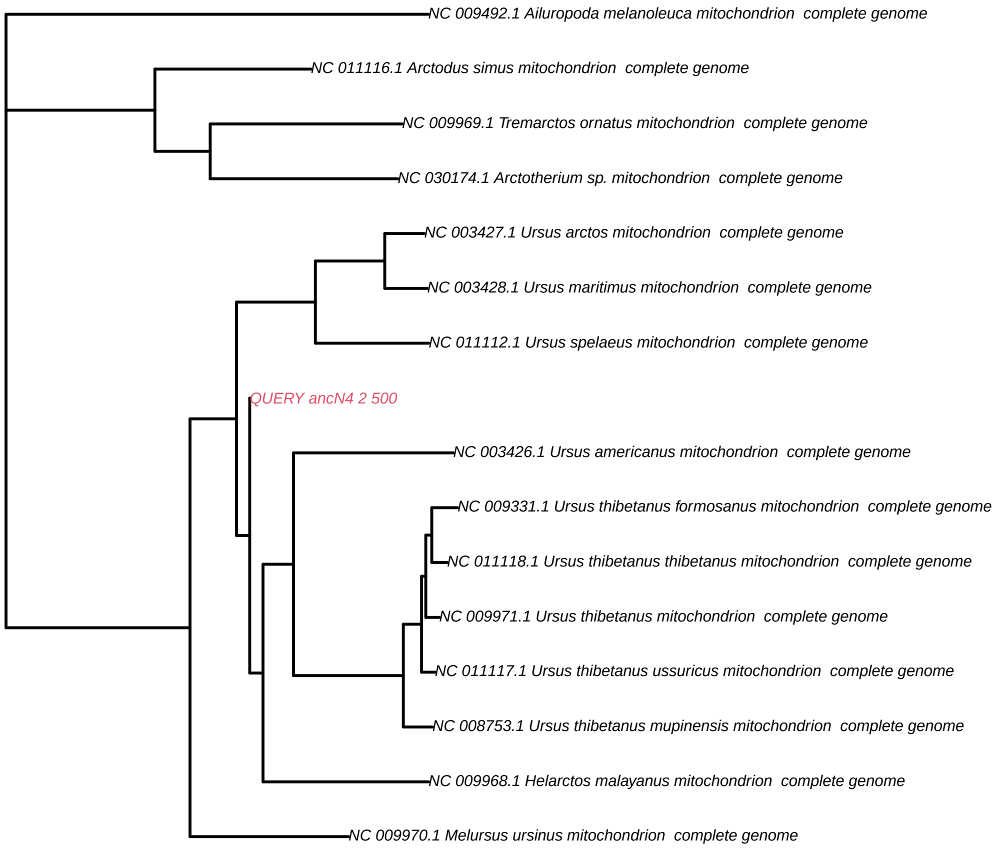

Figure 7: `pathPhynder` maximum likelihood method results for the simulated ancient single-source sample N4 at $\sim 1.3x$ coverage. `pathPhynder` shows the best path for the given sample, which ends at the correct ancestral node N4.

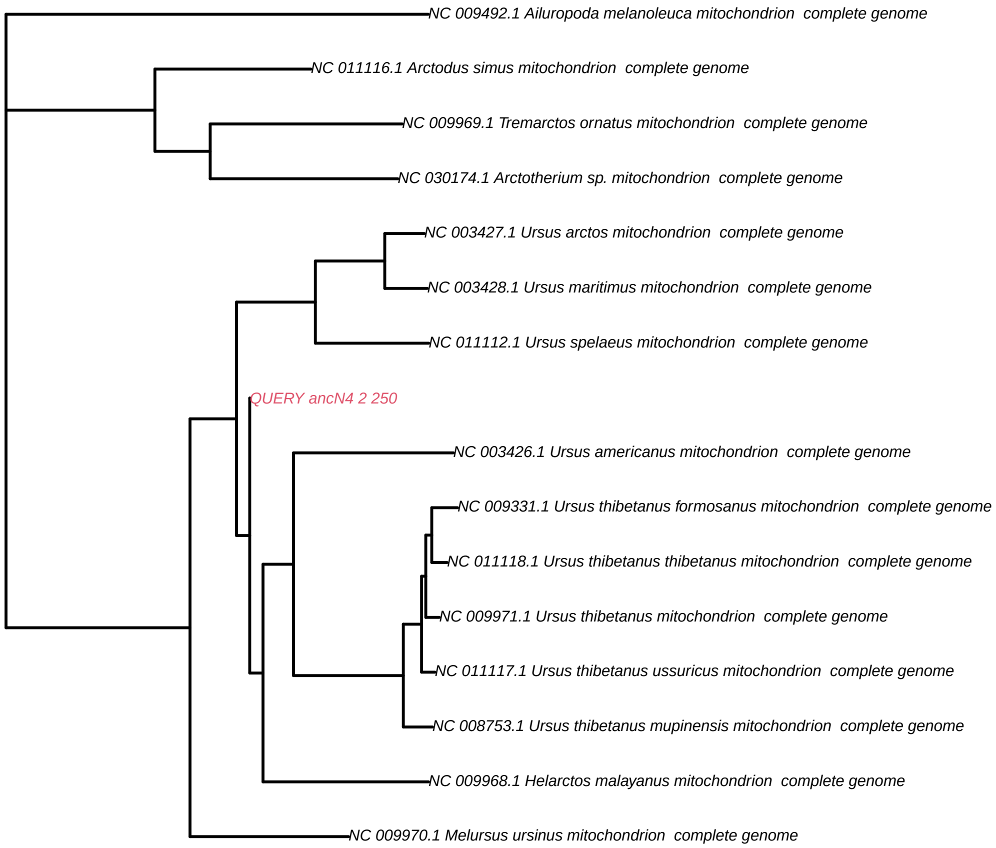

Figure 8: `pathPhynder` maximum likelihood method results for the simulated ancient single-source sample N4 at $\sim 0.66X$ coverage. `pathPhynder` shows the best path for the given sample, which ends at the correct ancestral node N4.

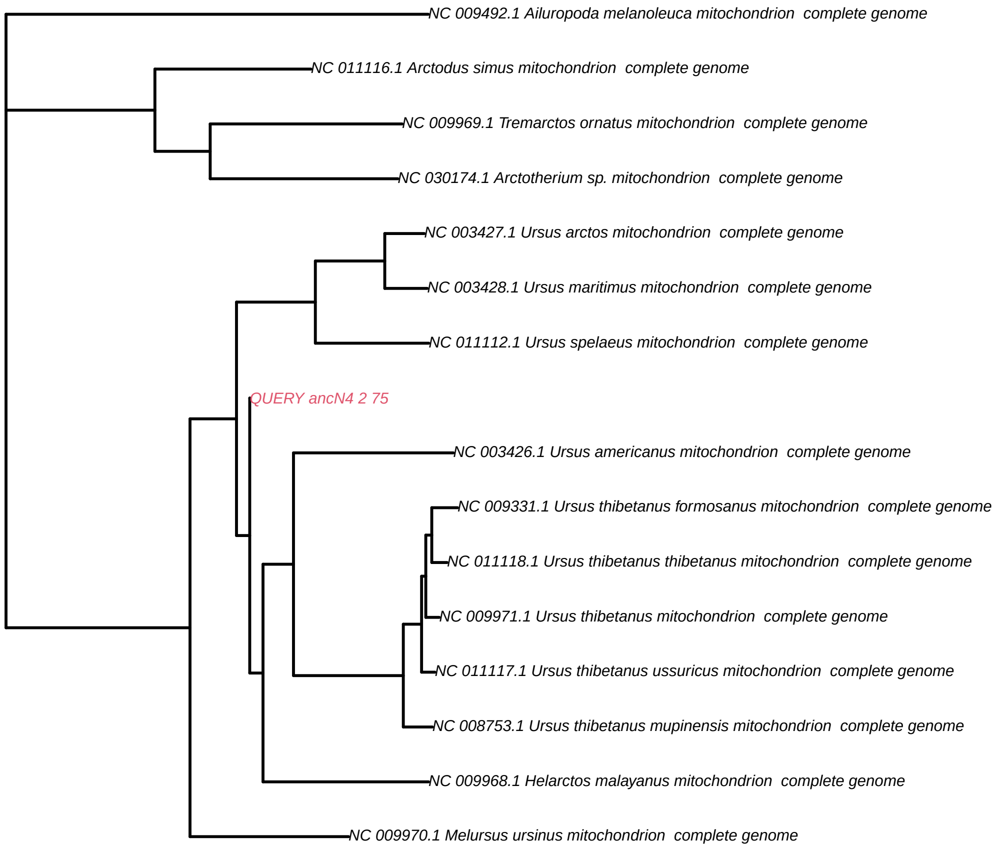

Figure 9: `pathPhynder` maximum likelihood method results for the simulated ancient single-source sample N4 at $\sim 0.2X$ coverage. `pathPhynder` shows the best path for the given sample, which ends at the correct ancestral node N4.

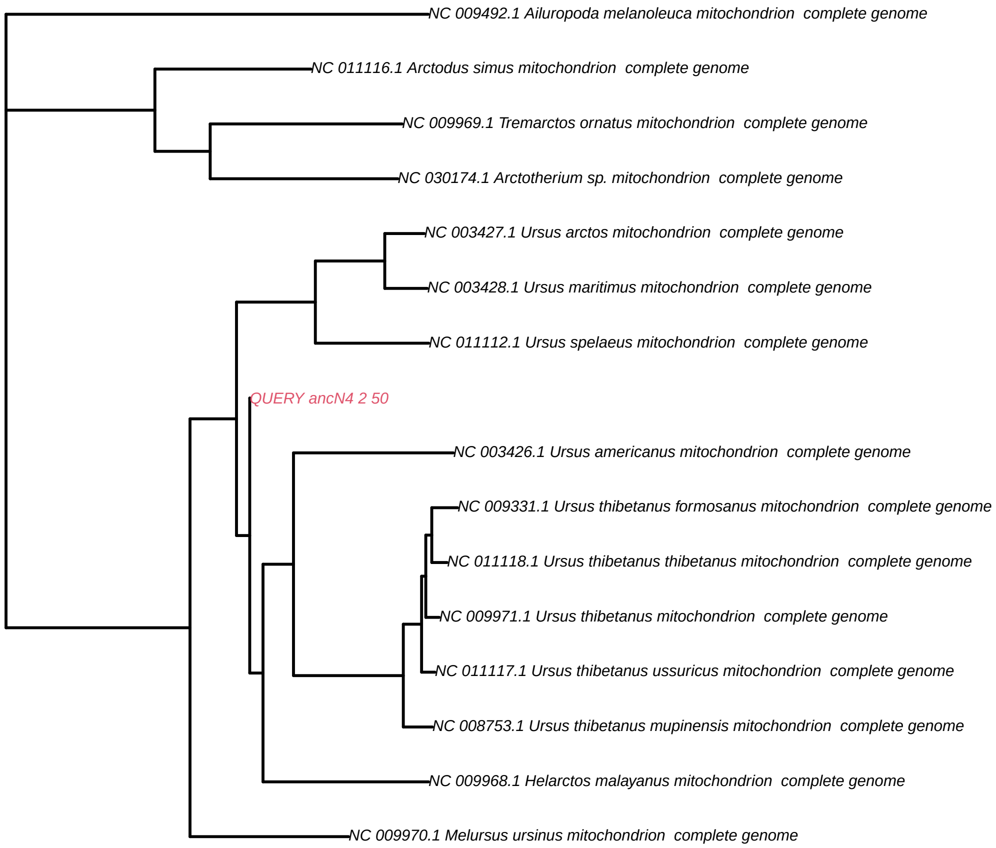

Figure 10: `pathPhynder` maximum likelihood method results for the simulated ancient single-source sample N4 at $\sim 0.13X$ coverage. `pathPhynder` shows the best path for the given sample, which ends at the correct ancestral node N4.

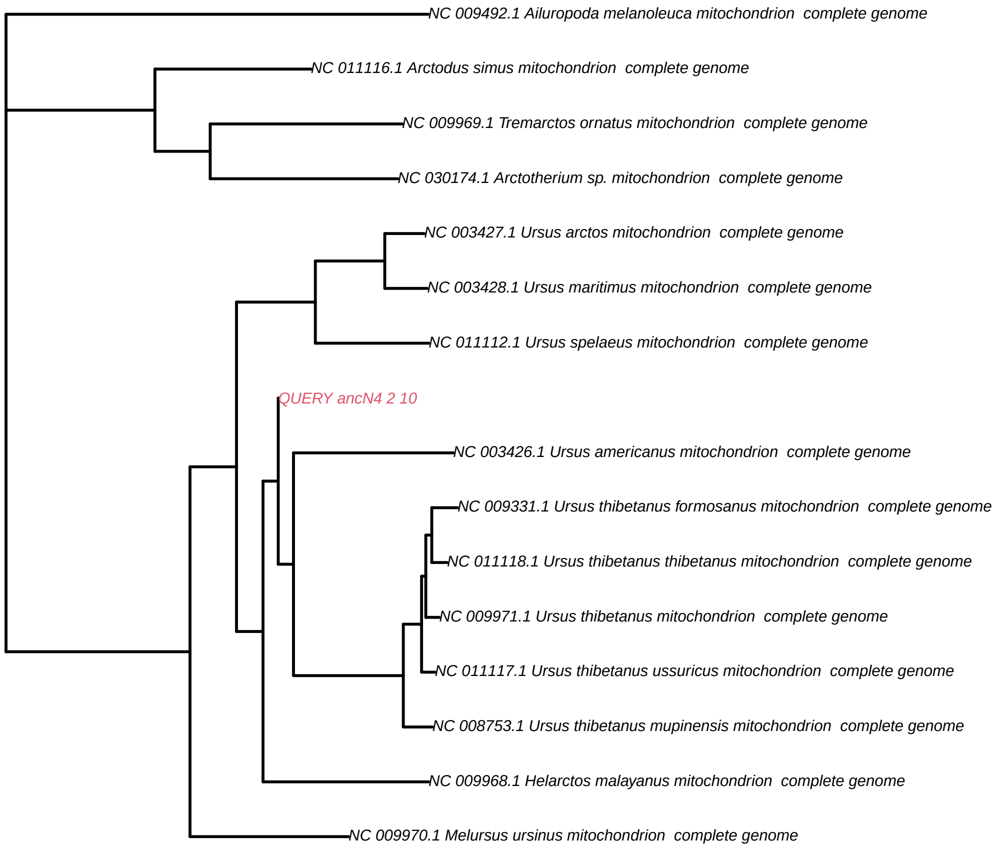

Figure 11: `pathPhynder` maximum likelihood method results for the simulated ancient single-source sample N4 at $\sim 0.026X$ coverage. `pathPhynder` shows the best path for the given sample, which ends at the ancestral node N6.

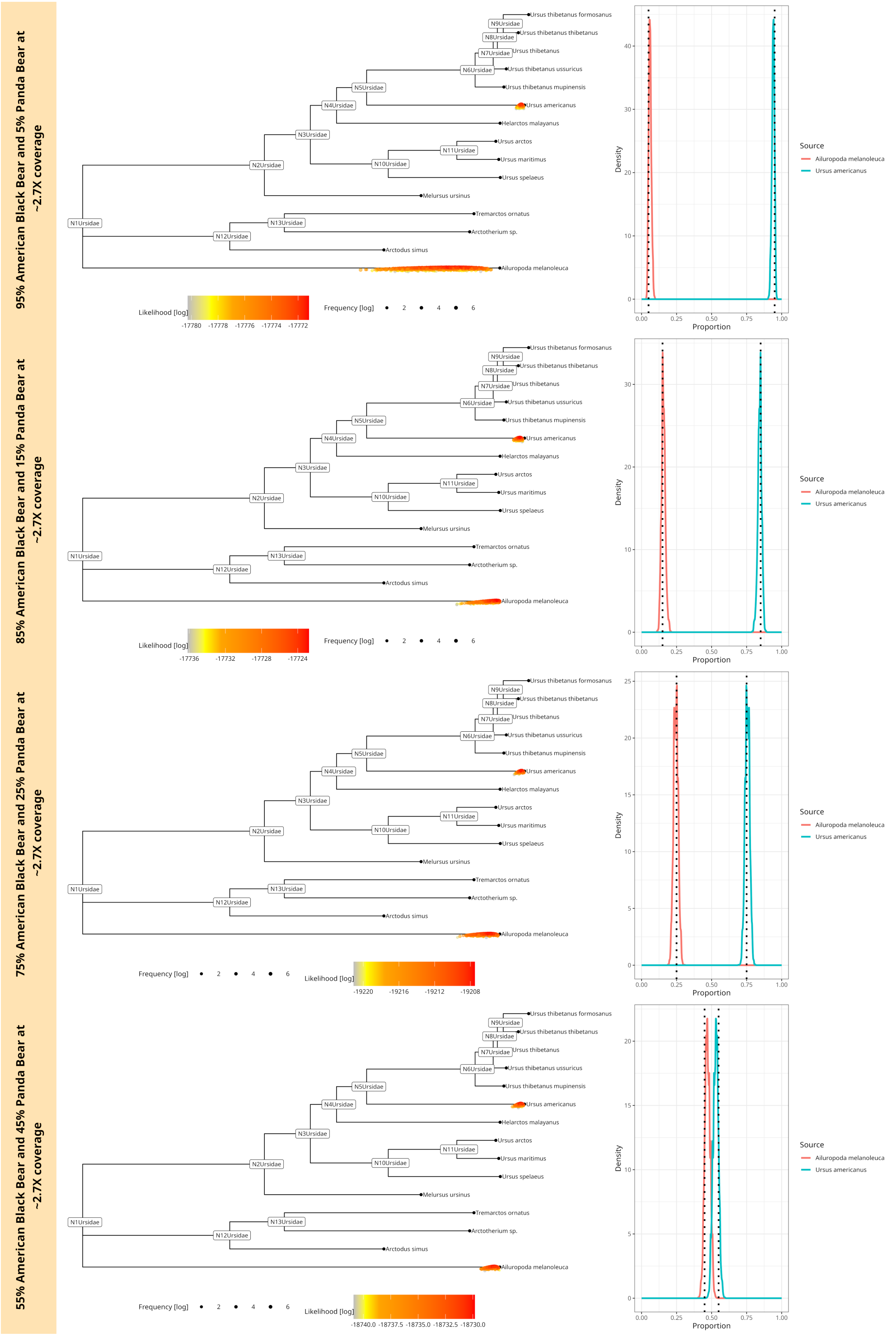

Figure 12: `soibean` results for simulated ancient fragments of two diverged species (83.4% similarity) from the family bears (Ursidae) at $\sim 2.7X$ coverage. The plot shows four different mixtures at $55\% - 45\%, 75\% - 25\%, 85\% - 15\%$ and $95\% - 5\%$ of the American Black bear and Giant Panda bear. The corresponding phylogenetic trees are displayed on the left: we plotted every accepted MCMC move coloured by likelihood value on the tree. The accepted moves are positioned above or below the tree, corresponding to a higher or lower likelihood value than the median, respectively. Each neighbouring plot shows the posterior proportion distribution, including the simulated proportion with a black dotted line.

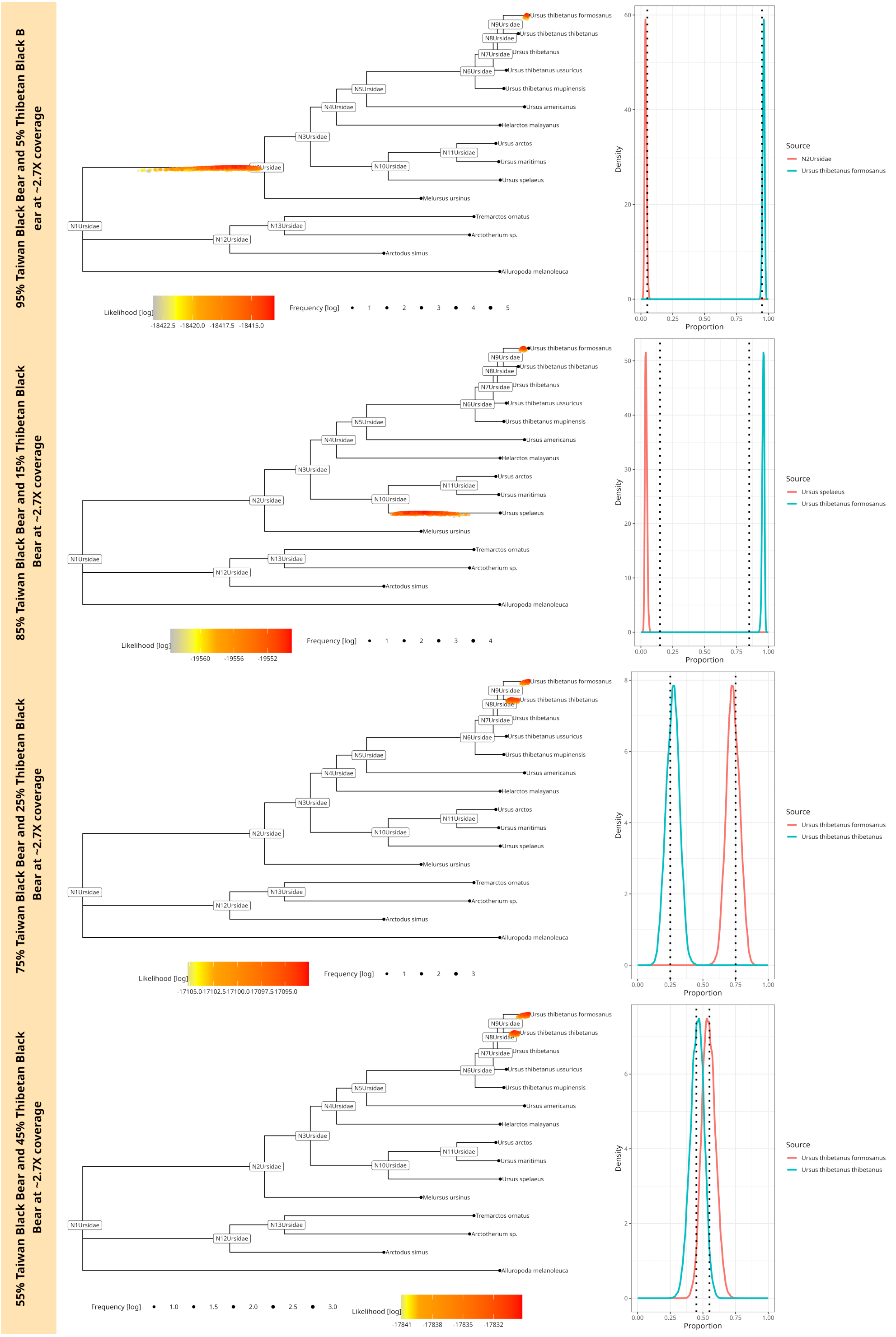

Figure 13: `soibean` results for simulated ancient fragments of two closely related species (98.8% similarity) from the family bears (Ursidae) at $\sim 2.7X$ coverage. The plot shows four different mixtures at $55\% - 45\%, 75\% - 25\%, 85\% - 15\%$ and $95\% - 5\%$ of the Taiwan and Tibetan Black bear. The corresponding phylogenetic trees are displayed on the left: we plotted every accepted MCMC move coloured by likelihood value on the tree. The accepted moves are positioned above or below the tree, corresponding to a higher or lower likelihood value than the median, respectively. Each neighbouring plot shows the posterior proportion distribution, including the simulated proportion with a black dotted line

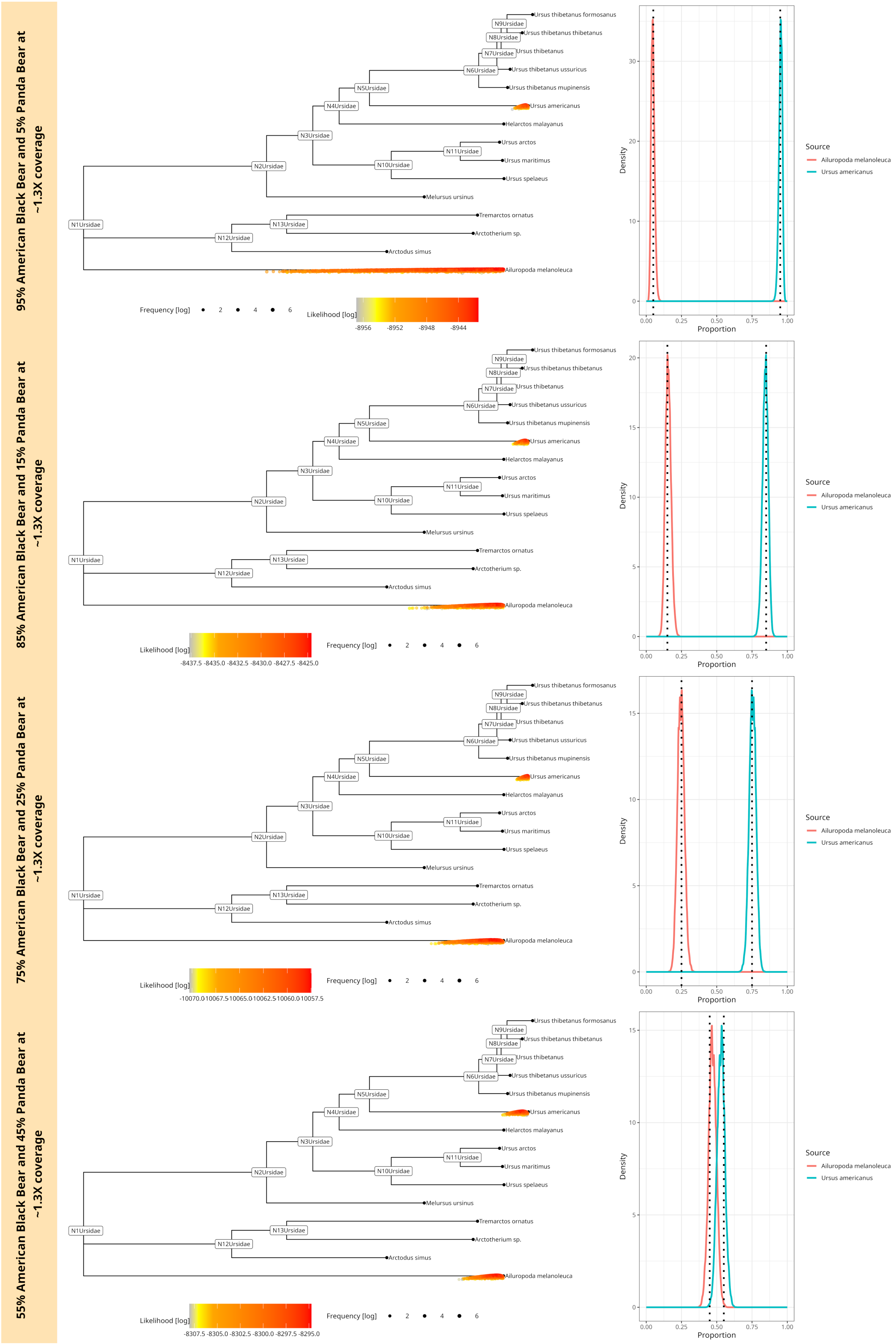

Figure 14: Mixtures of an American Black bear and a Giant Panda bear downsampled to $\sim 1.3X$ coverage (500 aDNA fragments). The plot shows four different mixtures at $55\% - 45\%, 75\% - 25\%, 85\% - 15\%$ and $95\% - 5\%$. The phylogenetic tree has a coloured point for each accepted MCMC move. The colour corresponds to the log-likelihood value. The posterior proportion distribution, including the simulated true proportion (black dotted line), is plotted on the right.

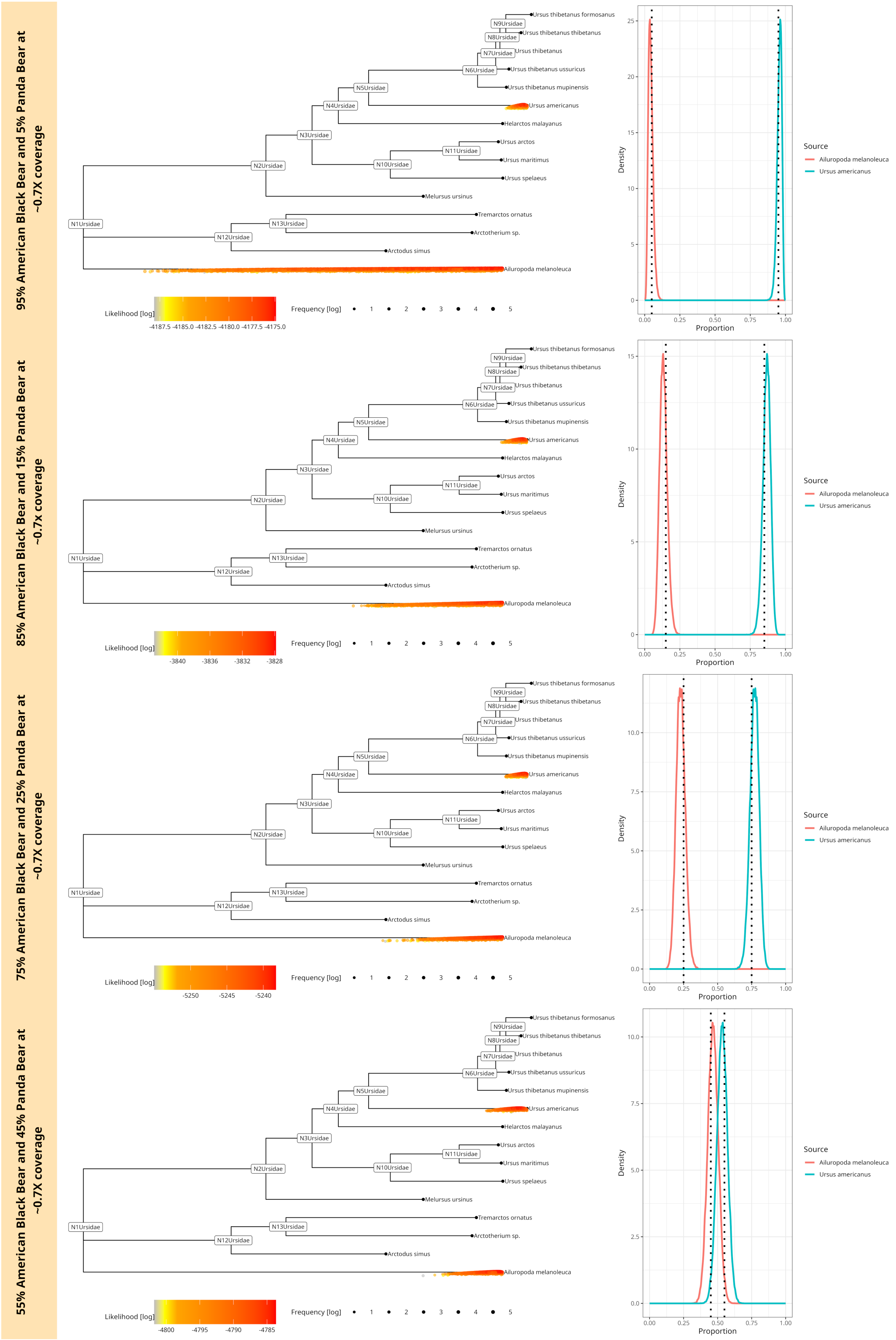

Figure 15: Mixtures of an American Black bear and a Giant Panda bear downsampled to $\sim 0.7x$ coverage (250 aDNA fragments). The plot shows four different mixtures at $55\% - 45\%, 75\% - 25\%, 85\% - 15\%$ and $95\% - 5\%$. The phylogenetic tree has a coloured point for each accepted MCMC move. The colour corresponds to the log-likelihood value. The posterior proportion distribution, including the simulated true proportion (black dotted line), is plotted on the right.

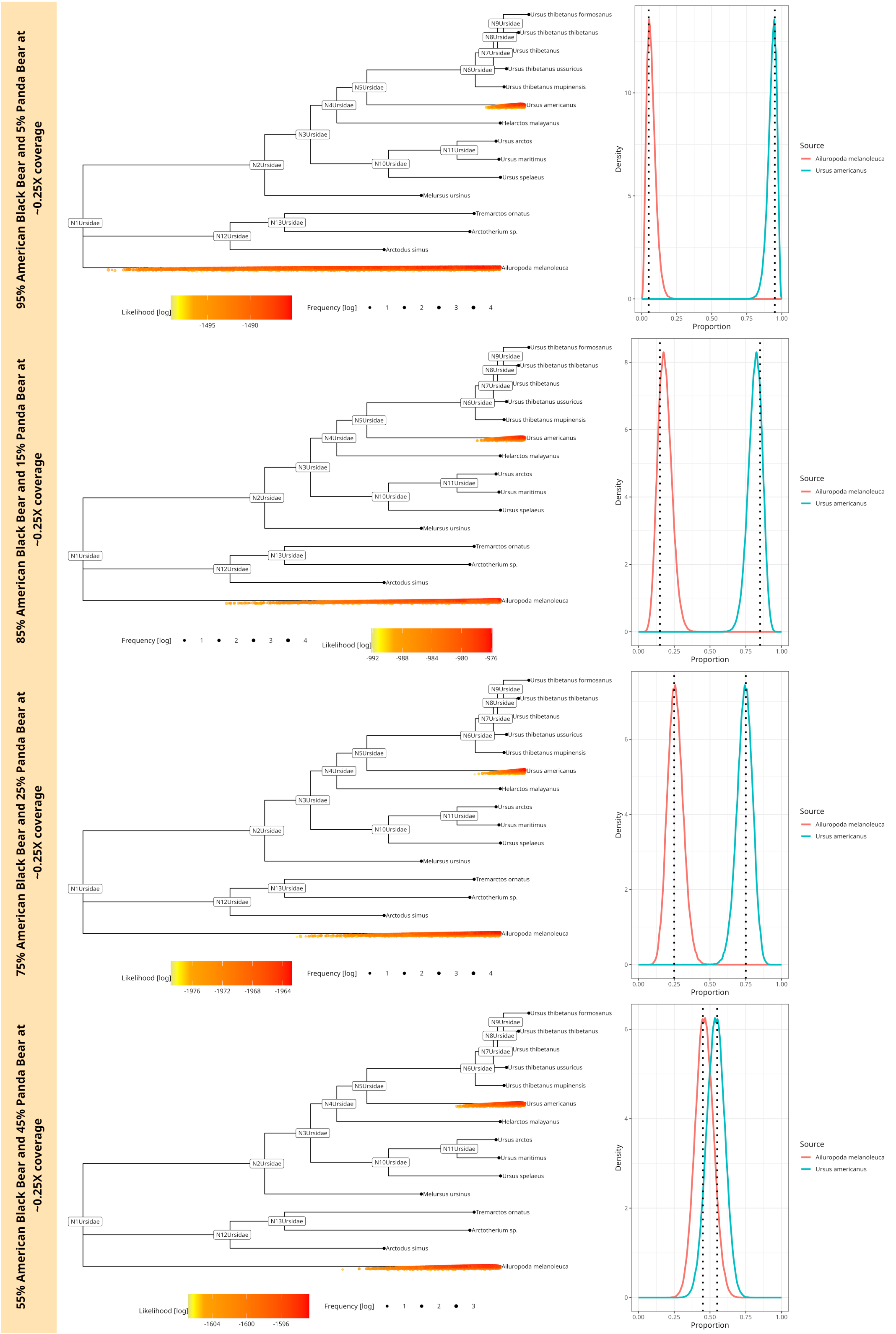

Figure 16: Mixtures of an American Black bear and a Giant Panda bear downsampled to $\sim 0.25x$ coverage (100 aDNA fragments). The plot shows four different mixtures at $55\% - 45\%, 75\% - 25\%, 85\% - 15\%$ and $95\% - 5\%$. The phylogenetic tree has a coloured point for each accepted MCMC move. The colour corresponds to the log-likelihood value. The posterior proportion distribution, including the simulated true proportion (black dotted line), is plotted on the right.

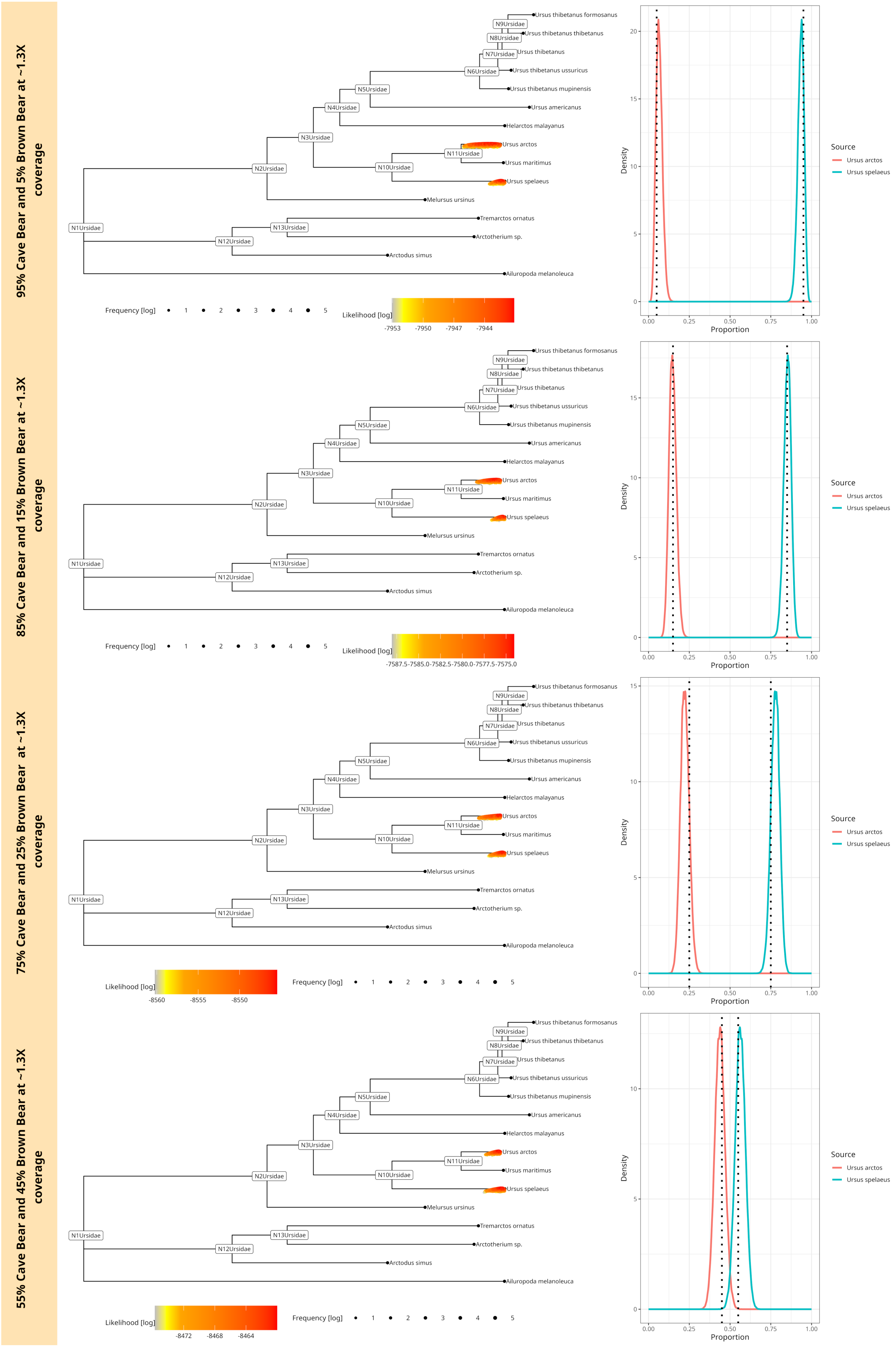

Figure 17: Cave bear and Brown bear mixture downsampled to $\sim 1.3X$ coverage (500 aDNA fragments). The plot shows four different mixtures at $55\% - 45\%, 75\% - 25\%, 85\% - 15\%$ and $95\% - 5\%$. The phylogenetic tree has a coloured point for each accepted MCMC move. The colour corresponds to the log-likelihood value. The posterior proportion distribution, including the simulated true proportion (black dotted line), is plotted on the right.

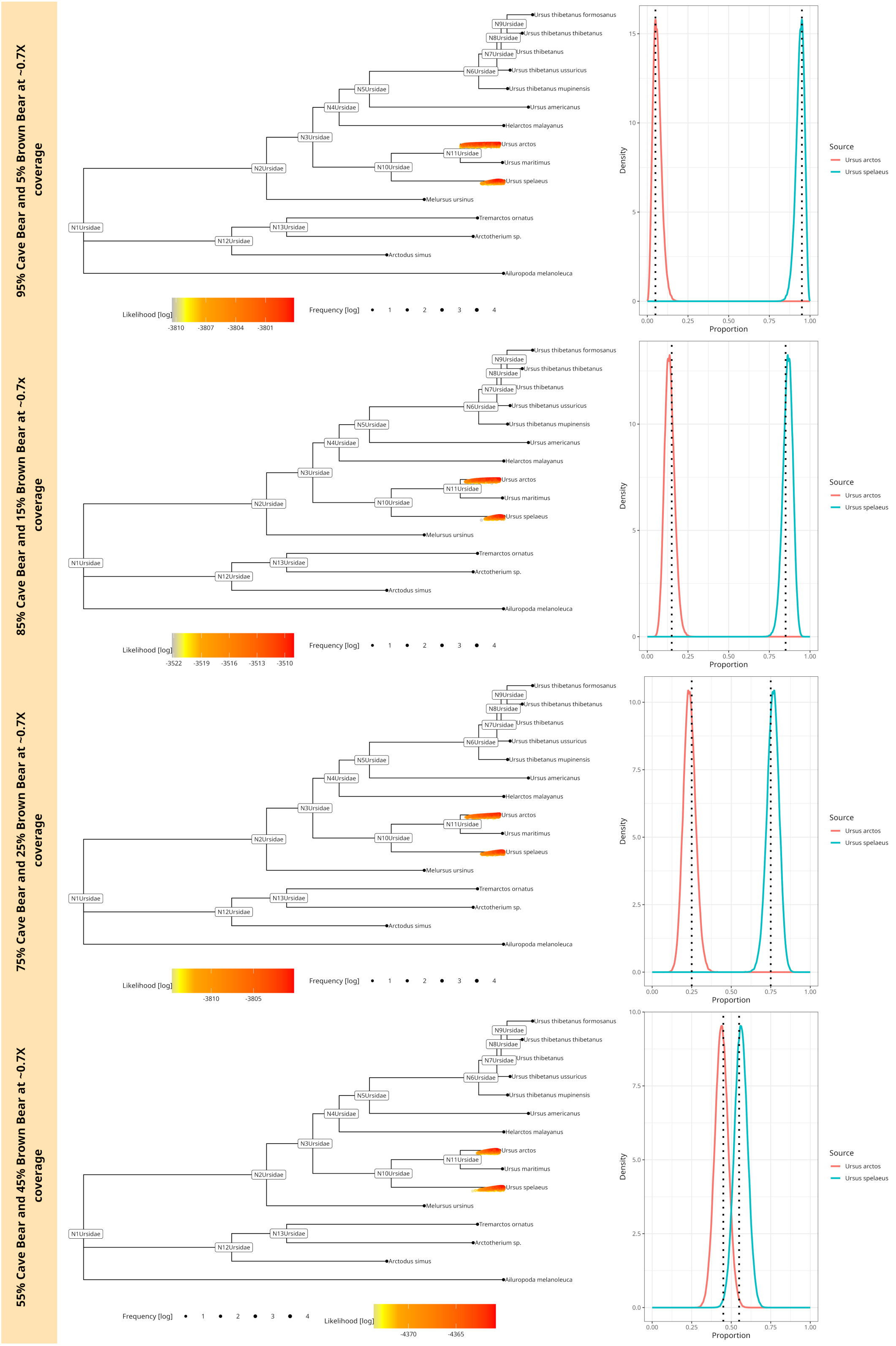

Figure 18: Cave bear and Brown bear mixture downsampled to $\sim 0.7X$ coverage (250 aDNA fragments). The plot shows four different mixtures at $55\% - 45\%, 75\% - 25\%, 85\% - 15\%$ and $95\% - 5\%$. The phylogenetic tree has a coloured point for each accepted MCMC move. The colour corresponds to the log-likelihood value. The posterior proportion distribution, including the simulated true proportion (black dotted line), is plotted on the right.

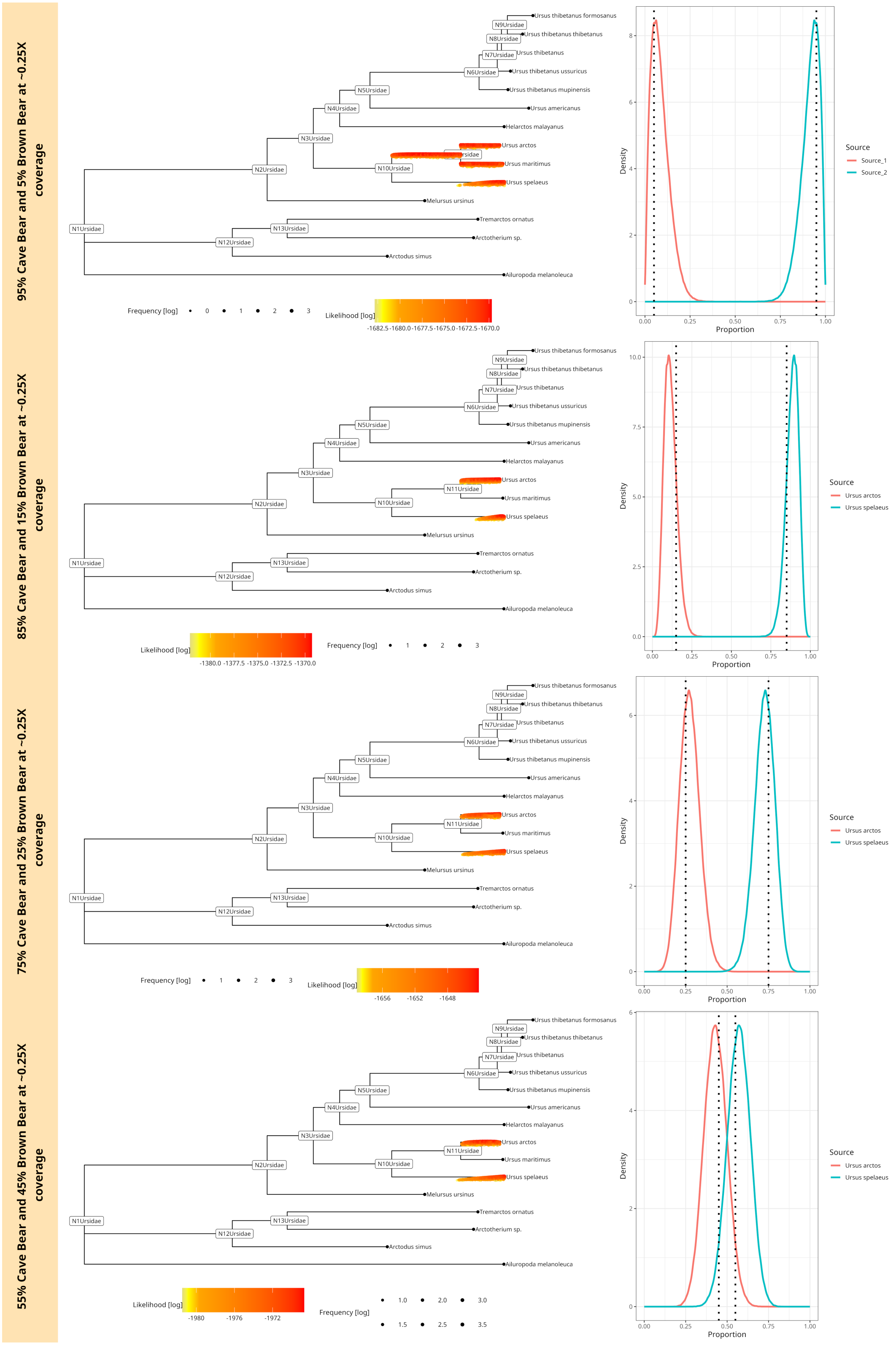

Figure 19: Mixtures of a Cave bear and a Brown bear downsampled to $\sim 0.25X$ coverage (100 aDNA fragments). The plot shows four different mixtures at $55\% - 45\%, 75\% - 25\%, 85\% - 15\%$ and $95\% - 5\%$. The phylogenetic tree has a coloured point for each accepted MCMC move. The colour corresponds to the log-likelihood value. The posterior proportion distribution, including the simulated true proportion (black dotted line), is plotted on the right.

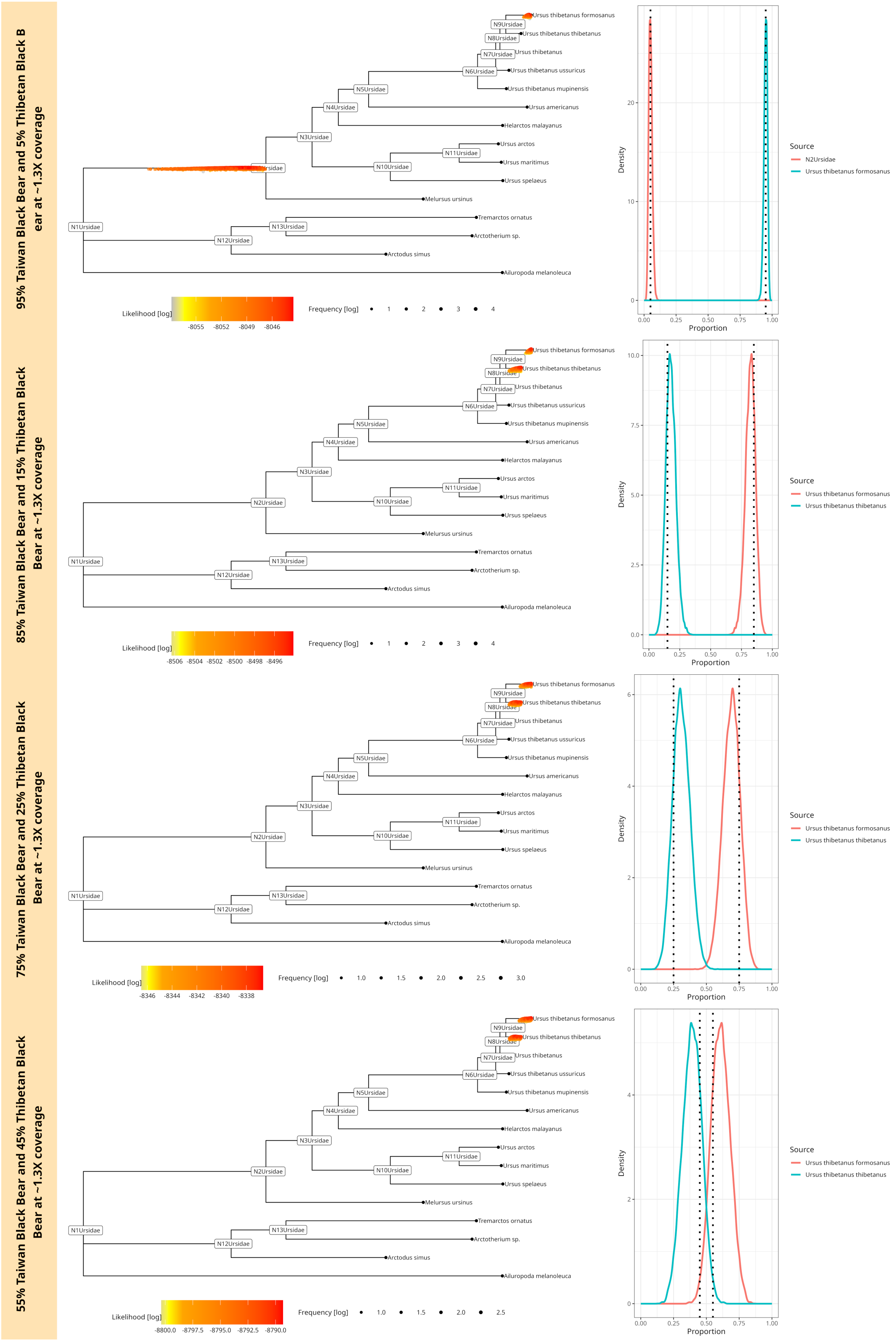

Figure 20: Mixtures of a Taiwan and Tibetan Black bear downsampled to $\sim 1.3X$ coverage (500 aDNA fragments). The plot shows four different mixtures at $55\% - 45\%, 75\% - 25\%, 85\% - 15\%$ and $95\% - 5\%$. The phylogenetic tree has a coloured point for each accepted MCMC move. The colour corresponds to the log-likelihood value. The posterior proportion distribution, including the simulated true proportion (black dotted line), is plotted on the right.

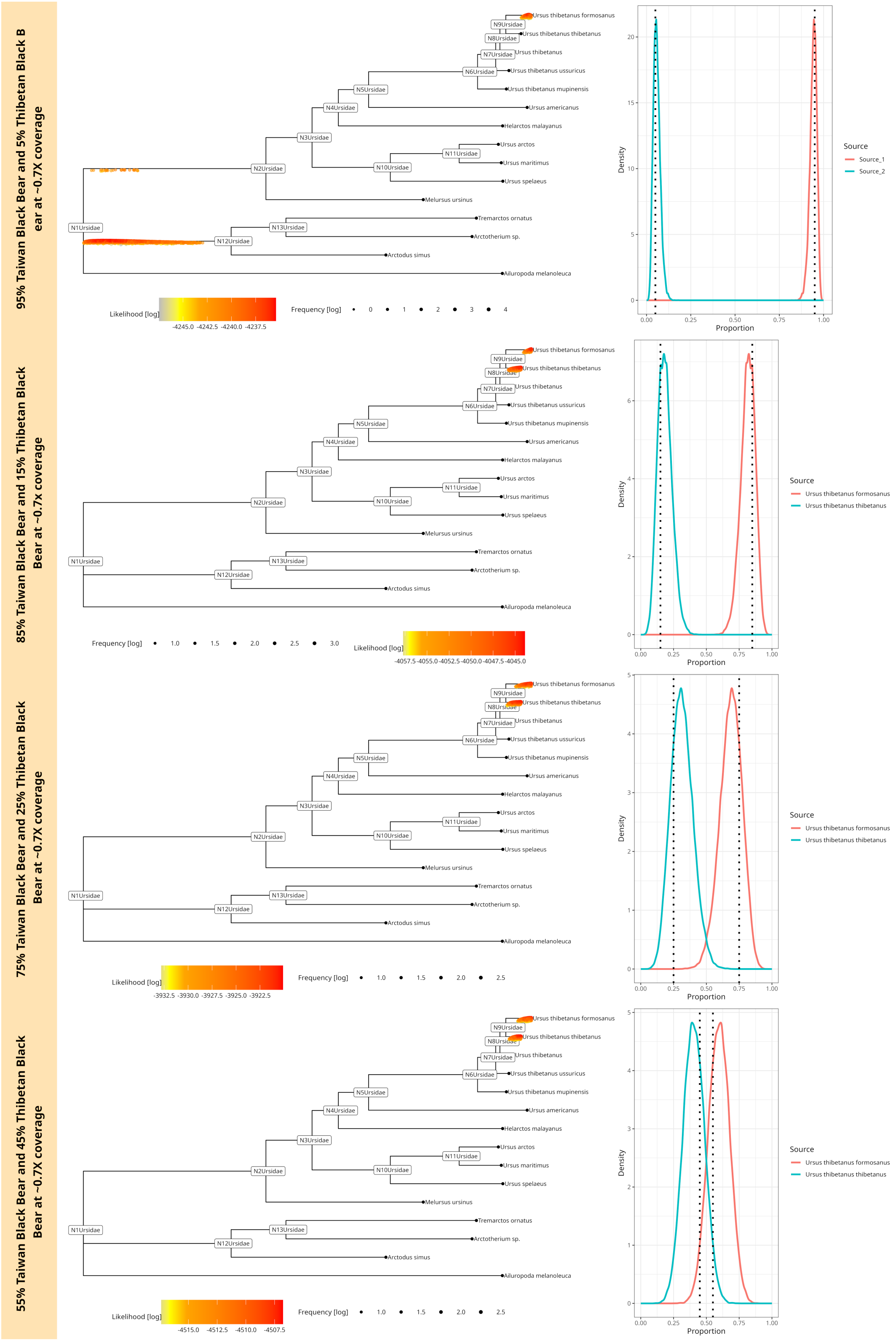

Figure 21: Mixtures of a Taiwan and Tibetan Black bear downsampled to $\sim 0.7X$ coverage (250 aDNA fragments). The plot shows four different mixtures at $55\% - 45\%, 75\% - 25\%, 85\% - 15\%$ and $95\% - 5\%$. The phylogenetic tree has a coloured point for each accepted MCMC move. The colour corresponds to the log-likelihood value. The posterior proportion distribution, including the simulated true proportion (black dotted line), is plotted on the right.

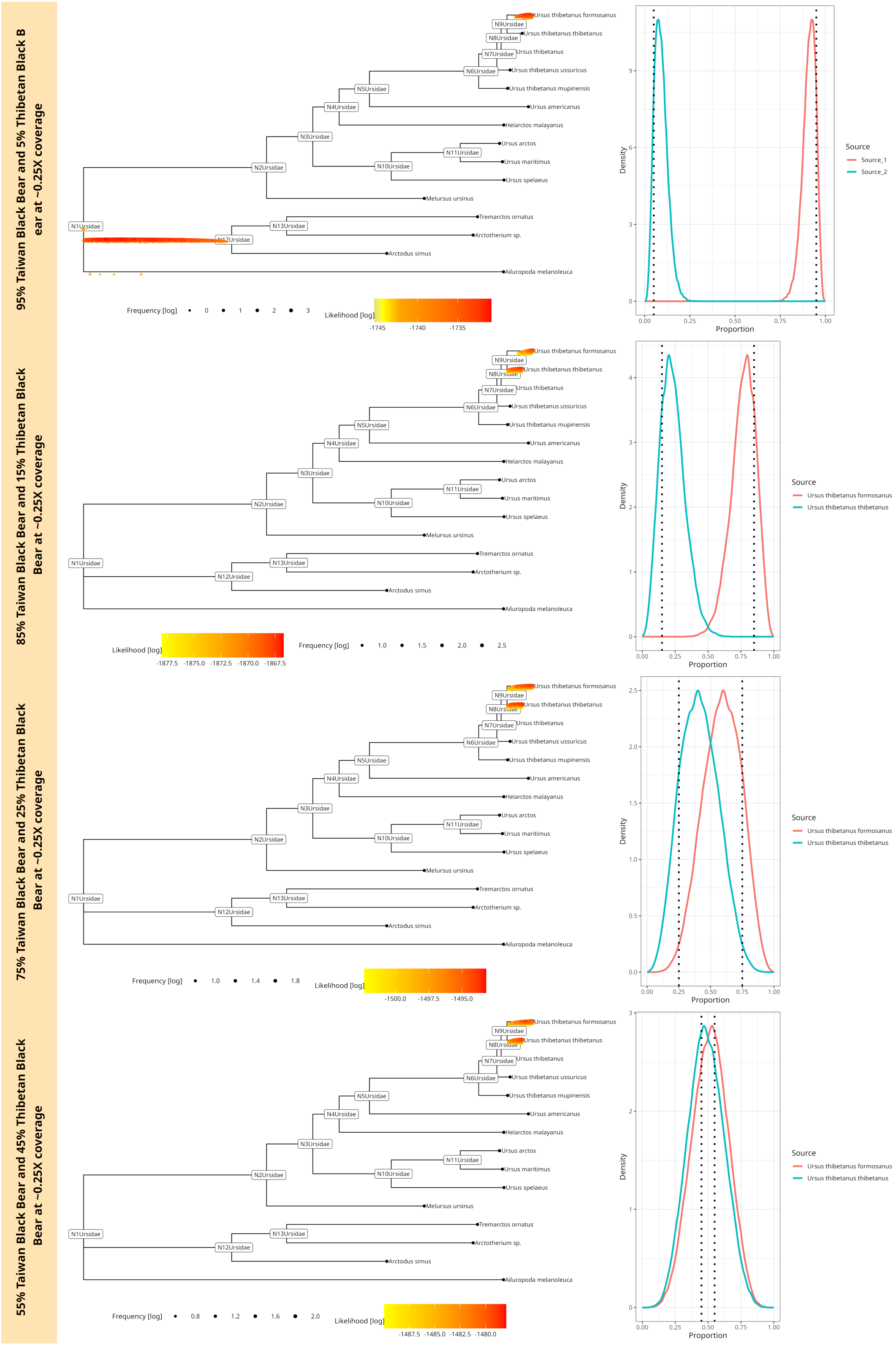

Figure 22: Mixtures of a Taiwan and Tibetan Black bear downsampled to $\sim 0.25X$ coverage (100 aDNA fragments). The plot shows four different mixtures at $55\% - 45\%, 75\% - 25\%, 85\% - 15\%$ and $95\% - 5\%$. The phylogenetic tree has a coloured point for each accepted MCMC move. The colour corresponds to the log-likelihood value. The posterior proportion distribution, including the simulated true proportion (black dotted line), is plotted on the right.

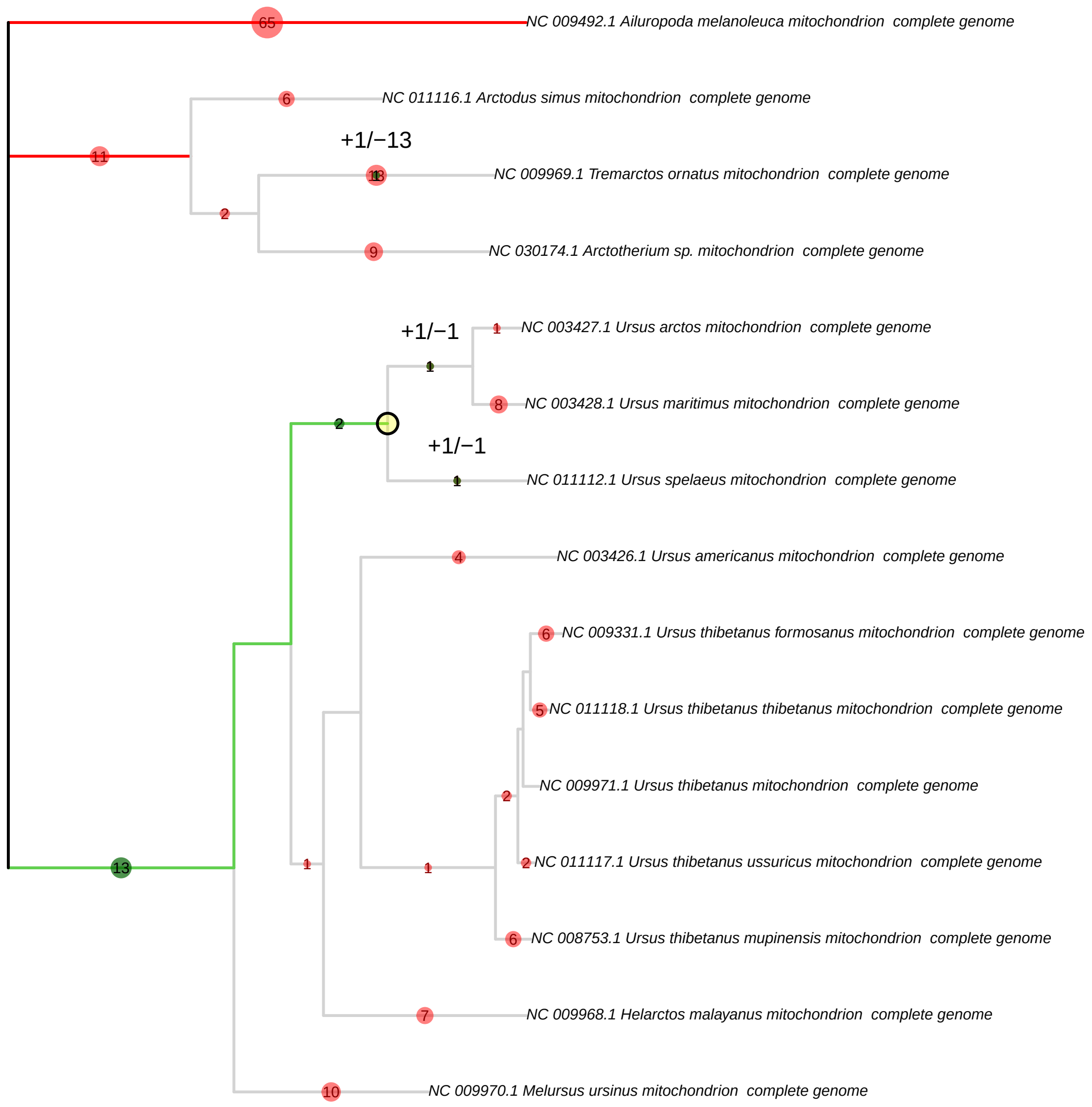

Figure 23: `pathPhynder` results for the simulated ancient mixture of a cave bear (*Ursus spelaeus*) 55% and the brown bear (*Ursus arctos*) 45% at $\sim 2.7X$ coverage. `pathPhynder` shows the best path for the given sample, which ends at the lowest common ancestor for the given mixture.

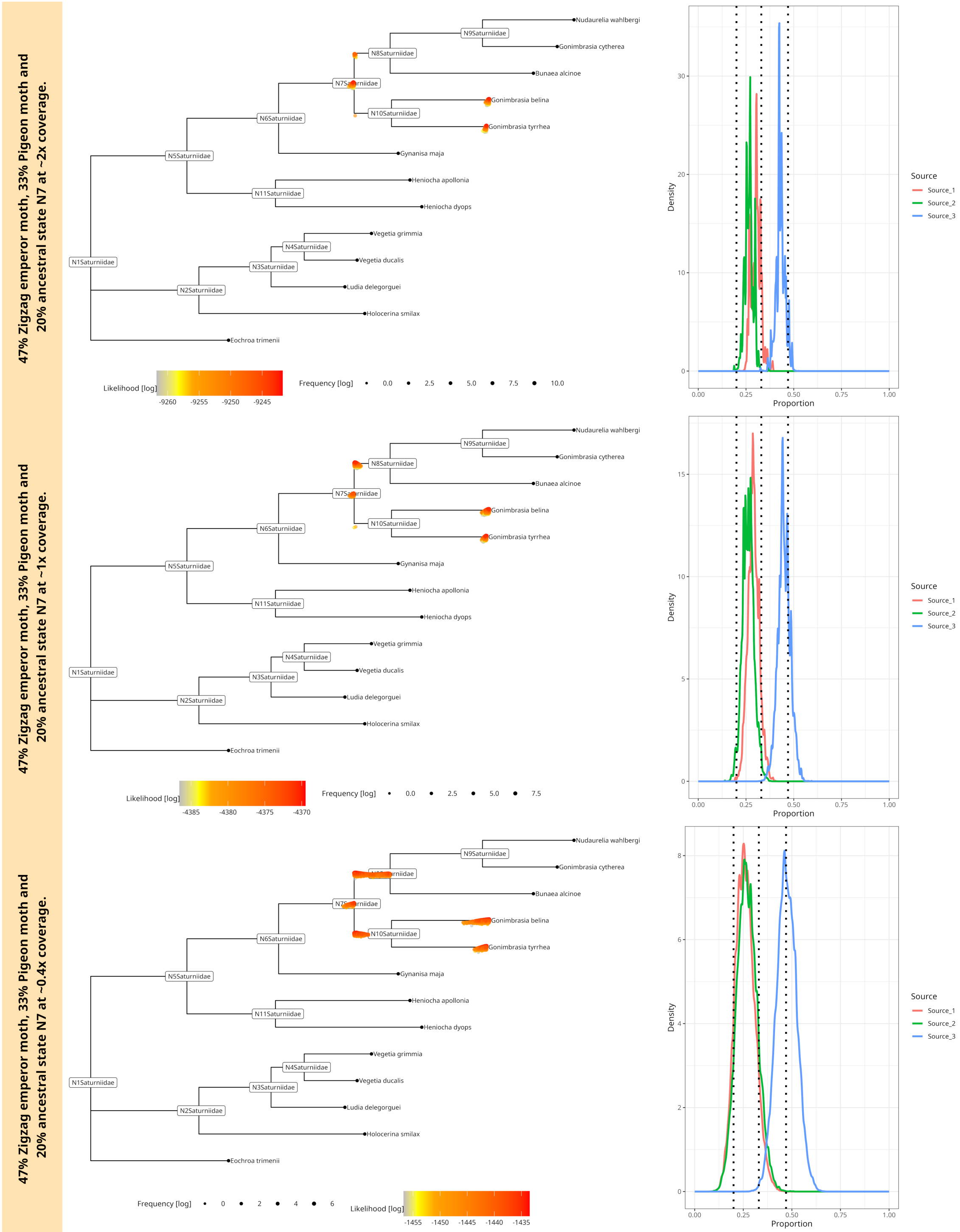

Figure 24: $44\% - 30\% - 23\%$ mixture of a three-source simulated ancient sample from a family of winged insects (Saturniidae). The mixture contains two emperor moths (*Gonimbrasia tyrrhea* and *Gonimbrasia belina* as well as the ancestral state N7 and is downsampled to $\sim 2X$ coverage (750 aDNA fragments), $\sim 1X$ coverage (375 aDNA fragments) and $\sim 0.4x$ coverage (150 aDNA fragments). The trees show every accepted MCMC move, coloured and placed by log-likelihood value (left side) and `soibean`'s proportion estimations on the left side, where the black dotted line shows the simulated proportion.

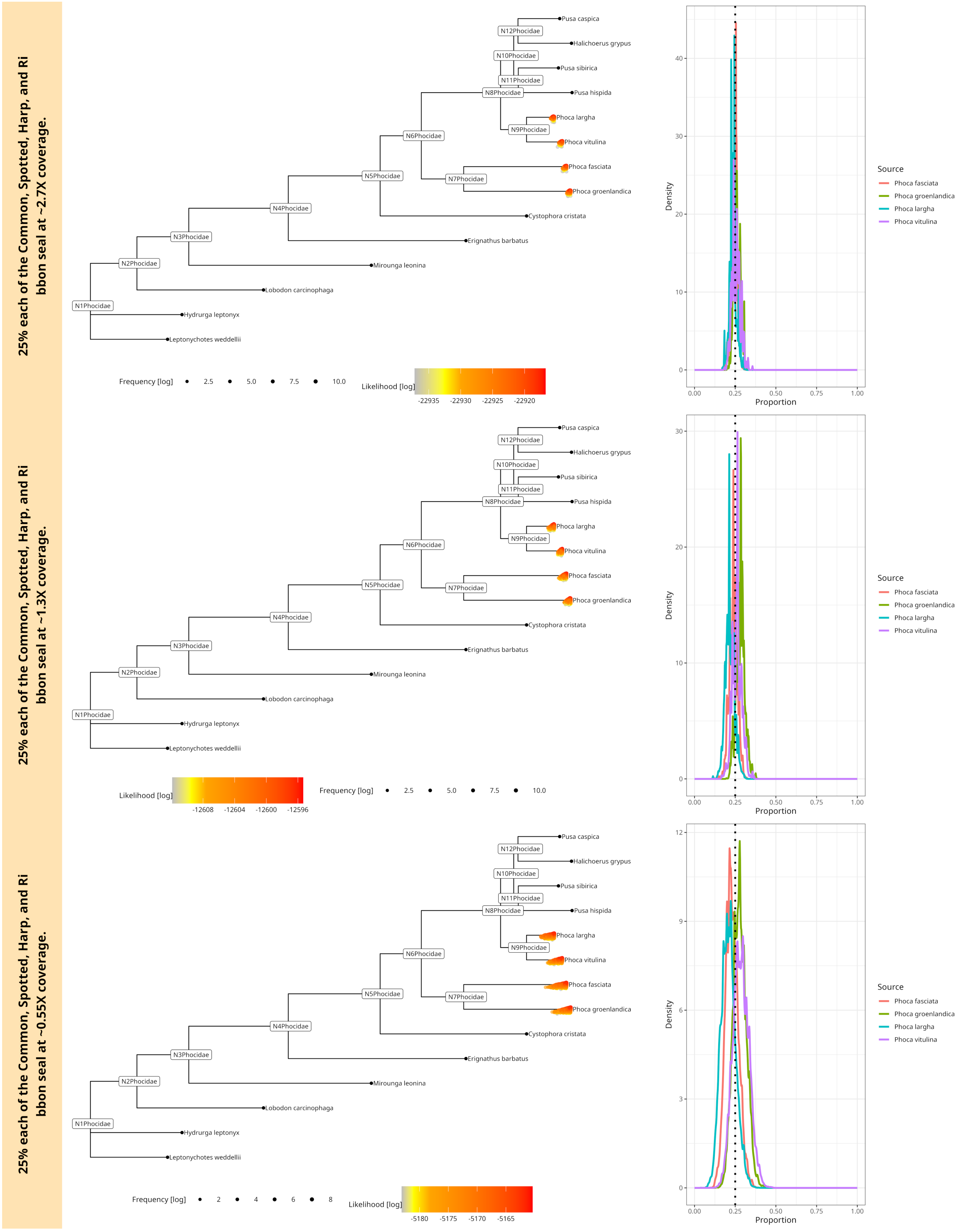

Figure 25: 25% – 25% – 25% – 25% mixture of a four-source simulated ancient sample from the family of seals (Phocidae). The mixture contains the earless seal species, namely *Phoca largha*, *Phoca vitulina*, *Phoca groenlandica* and *Pusa hispida*; it is downsampled to $\sim 2.7X$ coverage (1000 aDNA fragments), $\sim 1.3X$ coverage (500 aDNA fragments) and $\sim 0.55X$ coverage (200 aDNA fragments). The trees show every accepted MCMC move, coloured and placed by log-likelihood value (left side) and `soibean`'s proportion estimations on the left side, where the black dotted line shows the simulated proportion.

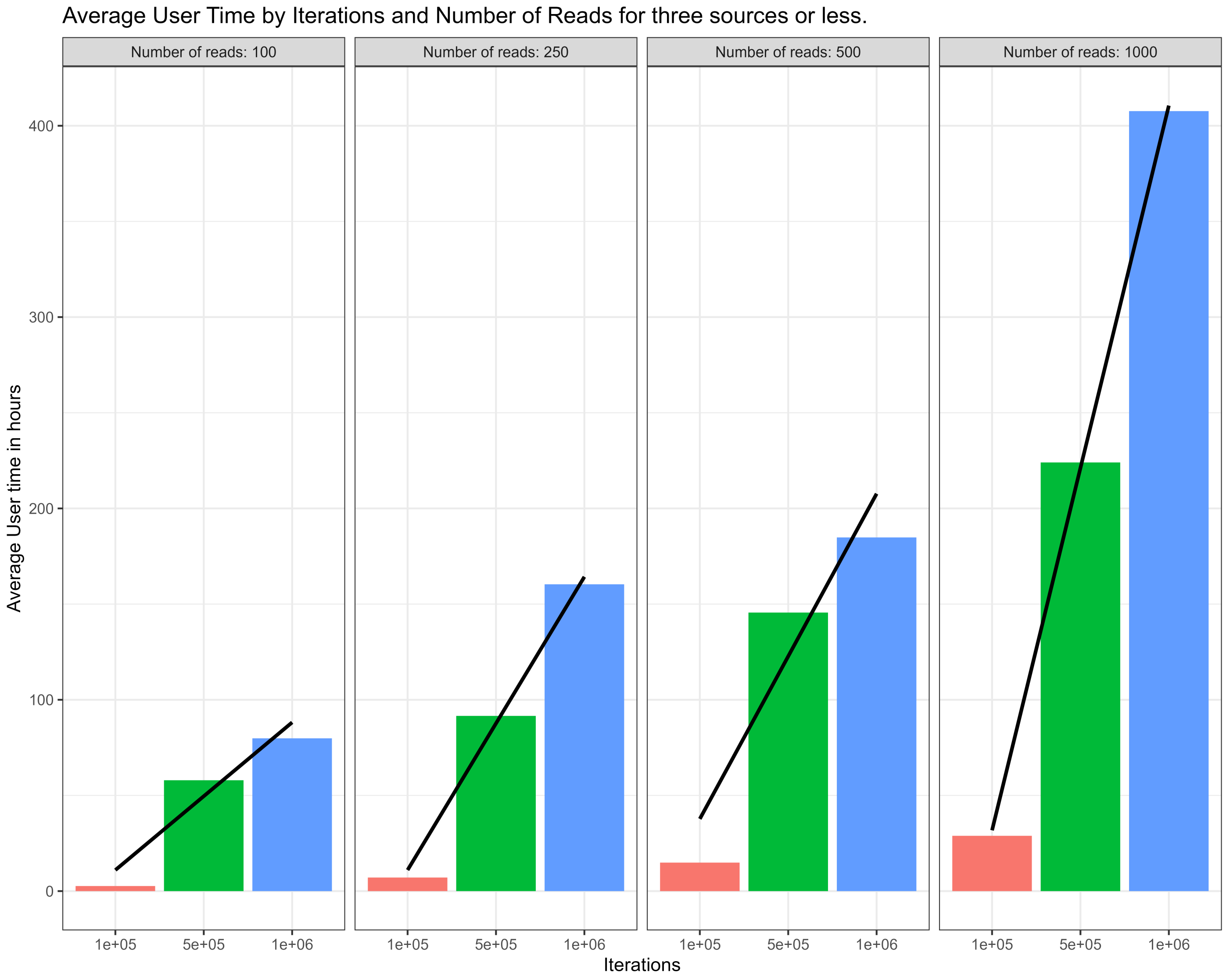

Figure 26: `soibean` user-time (hours) comparison for three different numbers of reads and three different numbers of iterations.

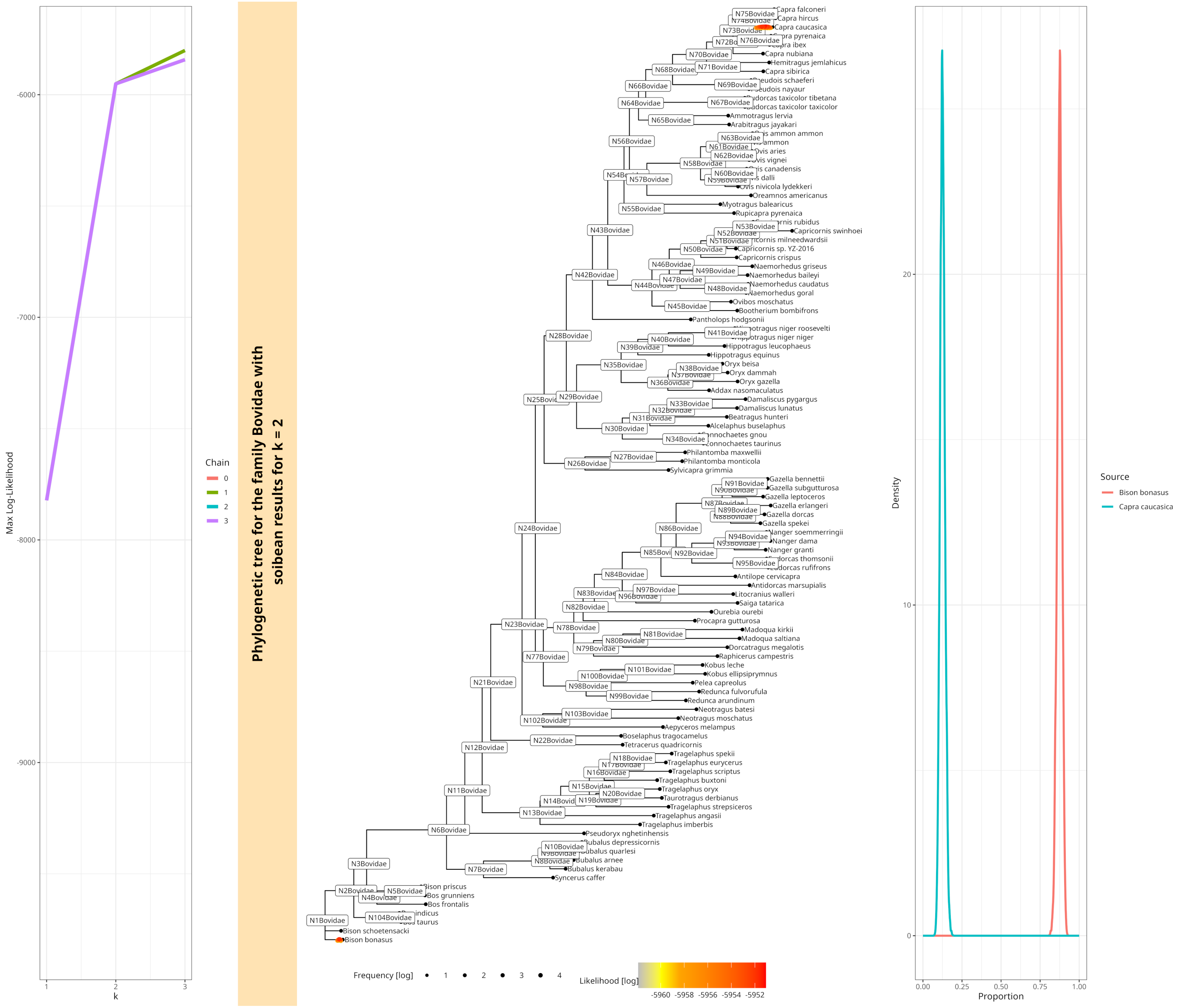

Figure 27: The results from `soibean` for the 25kya sediment sample from a Georgian cave. The k-curve highlights the presence of two contributing sources for the filtered Bovidae fragments. Analyzing the results, we can find the previously detected European Bison (*Bison bonasus*). Additionally, we can identify a low-coverage sample from the West Caucasian tur (*Capra caucasica*, with a proportion of about 10%, which was previously not reported.

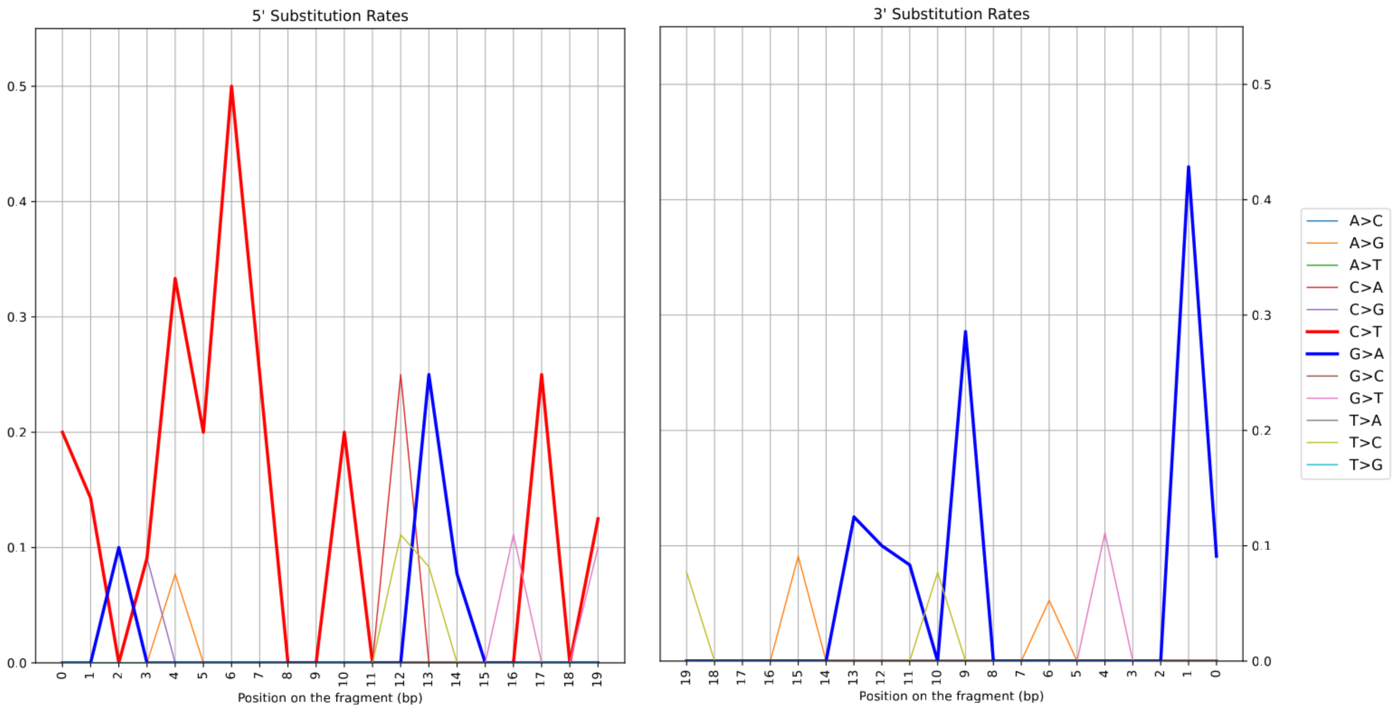

Figure 28: Deamination patterns for the alignments to the mitochondrial genome of *Capra caucasica*.

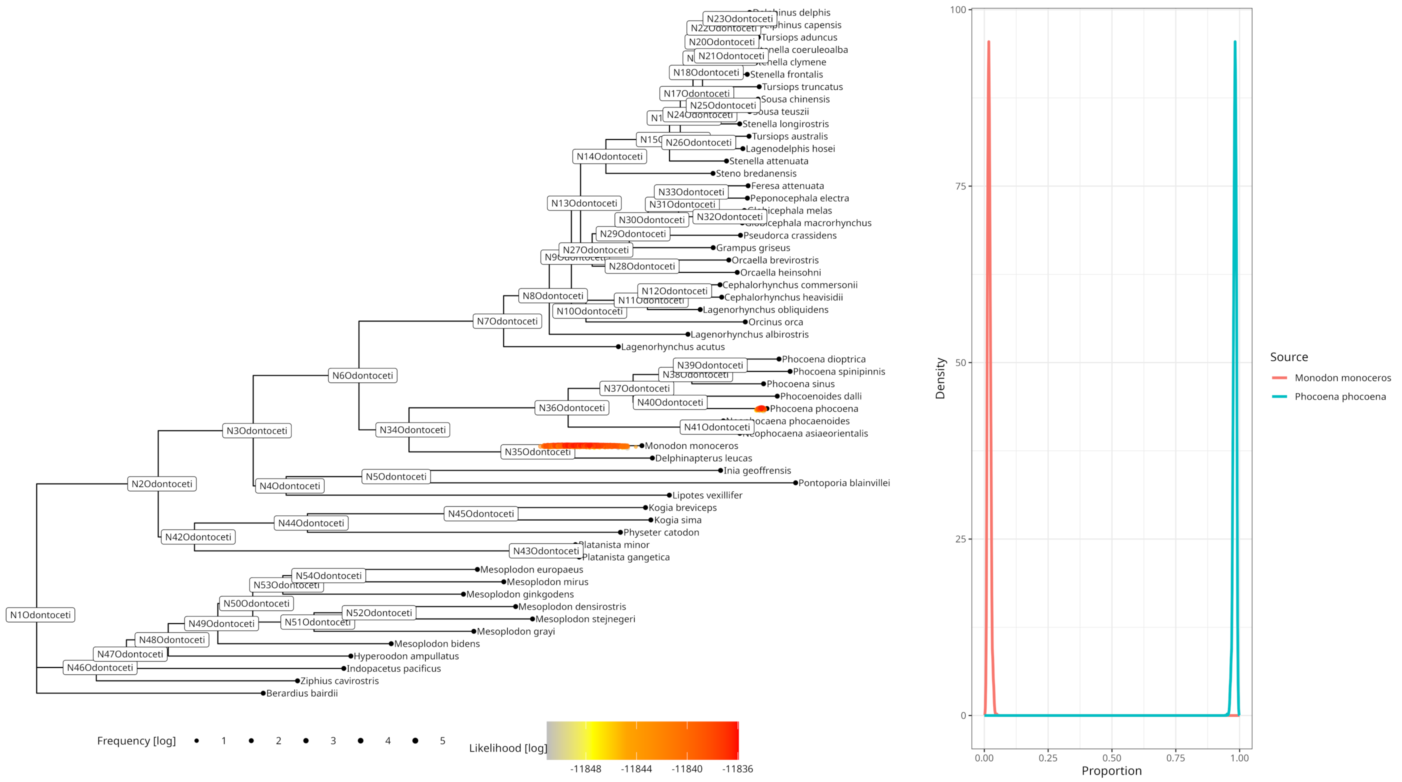

Figure 29: `soibean` results for the 9500-year-old empirical metagenomic sample from pitch pieces found in Huseby Klev on the northwestern coast of Sweden for $k = 3$. The harbour porpoise (*Phocoena phocoena*) is still the most likely source. `soibean` distributes one source at about 5% to an ancestral state of two Asiatic river dolphins. `soibean`'s branch and source proportion diagnostics showed an effective sample size below 200, indicating uncertainty for the second source.

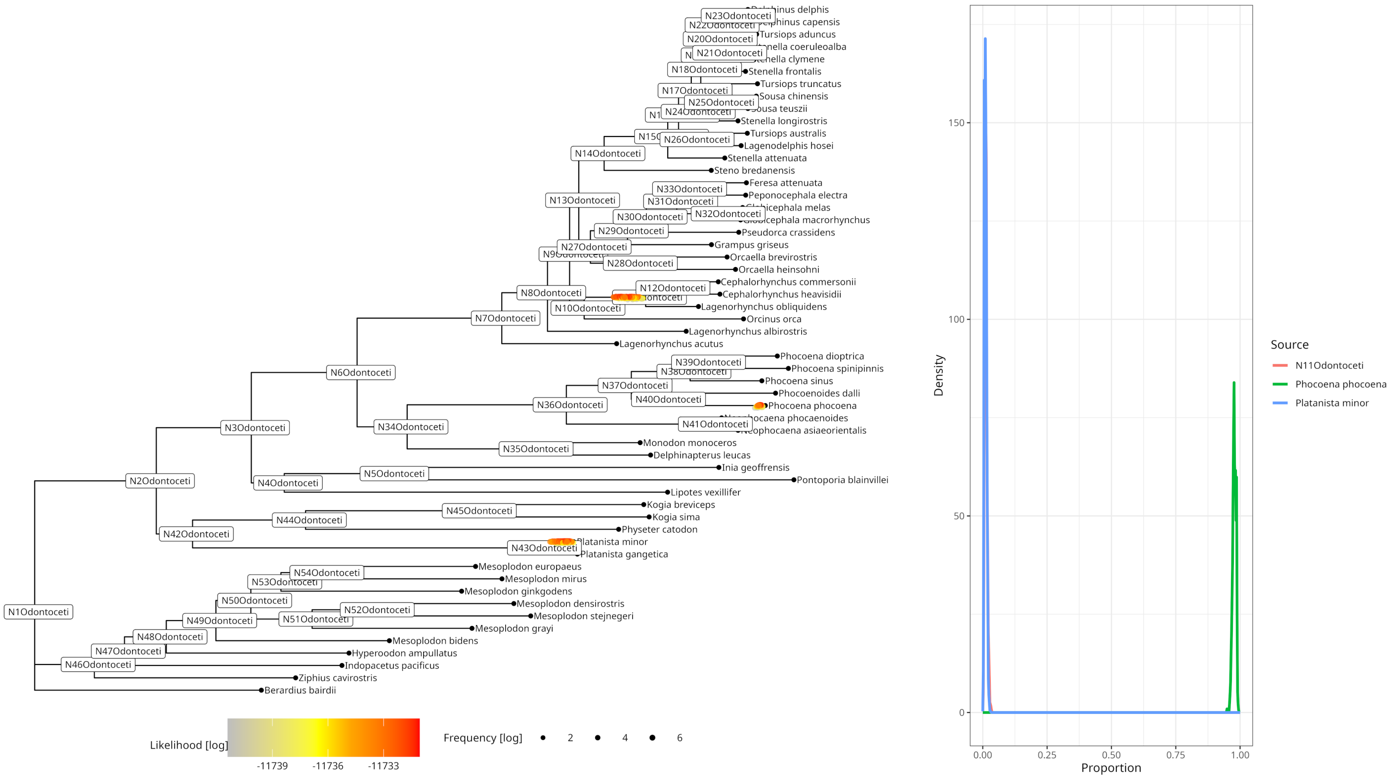

Figure 30: `soibean` results for the 9500-year-old empirical metagenomic sample from pitch pieces found in Huseby Klev on the northwestern coast of Sweden for $k = 3$. The harbour porpoise (*Phocoena phocoena*) is still the most likely source. `soibean` distributes two sources at about 2.5% to an ancestral state of two Asiatic river dolphins and a third dolphin species. Both additional sources have an effective sample size below 200 in `soibean`'s branch and source proportion diagnostics, making them unlikely contributing sources.

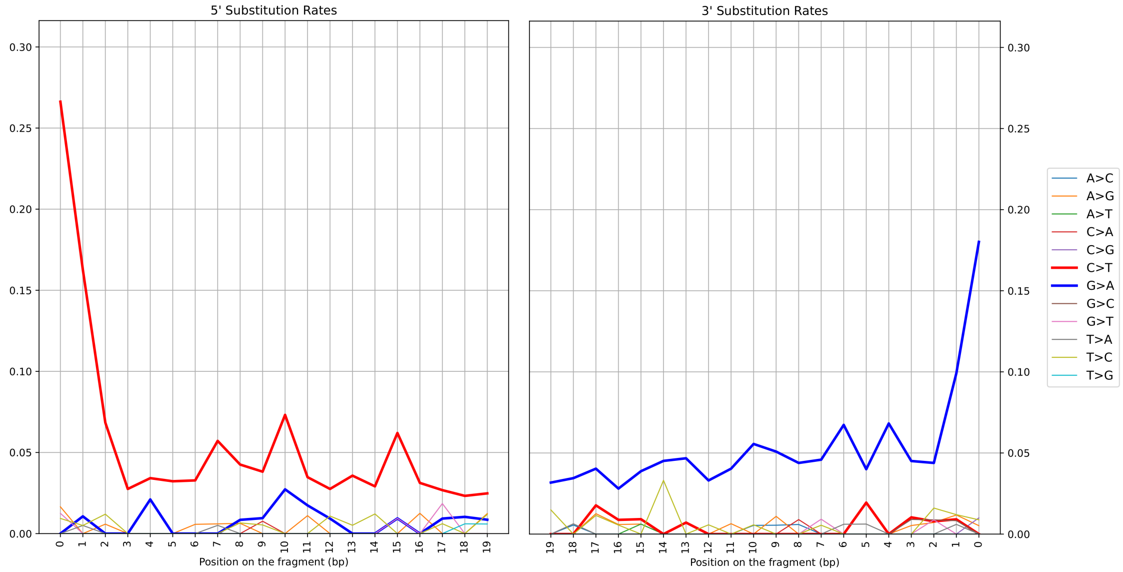

Figure 31: Complete deamination patterns for the alignments to the mitochondrial genome of *Phocoena phocoena*.
